# Phylogenetic and mutational analyses of human LEUTX, a homeobox gene implicated in embryogenesis

**DOI:** 10.1038/s41598-018-35547-5

**Published:** 2018-11-27

**Authors:** Shintaro Katayama, Vipin Ranga, Eeva-Mari Jouhilahti, Tomi T. Airenne, Mark S. Johnson, Krishanu Mukherjee, Thomas R. Bürglin, Juha Kere

**Affiliations:** 1 Department of Biosciences and Nutrition, Karolinska Institutet, Huddinge, Sweden; 2 Structural Bioinformatics Laboratory, Biochemistry, Faculty of Science and Engineering, Åbo Akademi University, Turku, Finland; 3 Department of Biomedicine, University of Basel, Basel, Switzerland; 4 Folkhälsan Institute of Genetics and Molecular Neurology Research Program, University of Helsinki, Helsinki, Finland; 5 School of Basic & Medical Biosciences, King’s College London, London, England; 6 Present Address: Research Programs Unit, Molecular Neurology and Biomedicum Stem Cell Centre, Faculty of Medicine, University of Helsinki, Helsinki, Finland; 7 Present Address: The Whitney Laboratory for Marine Bioscience, University of Florida, St. Augustine, USA

## Abstract

Recently, human PAIRED-LIKE homeobox transcription factor (TF) genes were discovered whose expression is limited to the period of embryo genome activation up to the 8-cell stage. One of these TFs is LEUTX, but its importance for human embryogenesis is still subject to debate. We confirmed that human LEUTX acts as a TAATCC-targeting transcriptional activator, like other K50-type PAIRED-LIKE TFs. Phylogenetic comparisons revealed that Leutx proteins are conserved across Placentalia and comprise two conserved domains, the homeodomain, and a Leutx-specific domain containing putative transcriptional activation motifs (9aaTAD). Examination of human genotype resources revealed 116 allelic variants in *LEUTX*. Twenty-four variants potentially affect function, but they occur only heterozygously at low frequency. One variant affects a DNA-specificity determining residue, mutationally reachable by a one-base transition. *In vitro* and *in silico* experiments showed that this LEUTX mutation (alanine to valine at position 54 in the homeodomain) results in a transactivational loss-of-function to a minimal TAATCC-containing promoter and a 36 bp motif enriched in genes involved in embryo genome activation. A compensatory change in residue 47 restores function. The results support the notion that human LEUTX functions as a transcriptional activator important for human embryogenesis.

## Introduction

Homeodomain transcription factors (TFs) contain mostly one or occasionally two or more homeodomains that bind DNA, and are often involved in regulating developmental processes of animals, fungi and plants^1–3^. Homeodomain proteins can be classified according to their amino acid sequences. The PAIRED (also known as PAX) and the PAIRED-LIKE (PRD-LIKE) classes are major classes of homeodomain proteins found in bilaterians. PRD-LIKE proteins lack the Paired domain found in PAIRED proteins, and although PAIRED and PRD-LIKE homeodomains are similar in amino-acid sequence, they are characterized by distinct residues at position 50 of the homeodomain^3^. Detailed experimental studies of homeodomain-DNA interactions have revealed important determinants that play key roles for specificity in various homeodomain classes, e.g.^4–10^. The structure of the homeodomain is comprised of three alpha helixes, of which the third “recognition” helix fits into the major groove of the DNA. Especially, position 50 in the third helix, which varies in PRD-LIKE TFs, plays a key role in determining divergent preferences for target DNA sequences^3,4,11–13^. For example, the target motif of human OTX2 (K50-type) is TAATCC, while that of PRRX1 (Q50-type) is TAATTR^14^. Likewise, when K50 in human LEUTX is mutated to an alanine residue, its transcriptional activity for a TATTCC-containing promoter is lost^15^.

Recent studies by our group and others have shown that many PRD-LIKE genes are transcribed in human reproductive tissues and during the preimplantation development; for example, *NOBOX* in ovary^16^, testis and oocytes^17^; and *ARGFX*, *CPHX1*, *CPHX2*, *DPRX*, *LEUTX, OTX2*, *TPRX1*, and *TPRX2* in oocytes to 8-cell stage blastomeres^16,18,19^. Among them, *LEUTX* is induced at the 4-cell stage^18^, and our further studies revealed that its expression is restricted to the 4-cell to 8-cell stage of the preimplantation embryo^15^. This period overlaps with human embryonic genome activation (EGA)^20^. Present evidence indicates that *LEUTX* is not expressed in any other cell types, including human embryonic stem cells (hESCs). Transfection and overexpression of human LEUTX in hESCs was able to activate the transcription of ~25% of the secondary genes induced at the 8-cell stage^15^, although hESCs are later descendants of the 8-cell blastomeres, by about 6–7 cell divisions. Interestingly, 36 bp DNA elements containing a TAATCC sequence motif, referred to as EEA motif (EGA-enriched Alu-motif), are over-represented in the promoters of genes activated in early embryos^18^, and also in genes upregulated by human LEUTX overexpression in hESCs^15^. Co-activation of this EEA-motif together with five TFs known for induction of pluripotent cells improved the reprogramming efficiency of primary skin fibroblasts^21^. Together, these data suggest an important contribution of human LEUTX for human embryogenesis.

A number of PRD-LIKE TFs (Argfx, Leutx, Dprx, Tprx, Leutx), which are expressed in early development^16,18^, have been evolving rapidly by gene duplication and diversification, and are thought to be derived from Crx, an Otx homeobox family member of the PRD-LIKE class^2,18,22^. Gene duplications are an essential source for evolutionary innovation, since one copy is free to diverge^23^, although in many instances the duplicated genes are likely to become pseudogenes^24^; Leutx genes have been reported to be absent in mouse or rat^15,25^. Moreover, the other Crx-derived PRD-LIKE TFs are also expressed in the same early embryonic period. Therefore, functional studies of the transcribed products as TFs are needed, and the implied importance of LEUTX for human embryogenesis needs further investigation to confirm its role, though this is a difficult task in human embryos.

In the present study, we address the role of LEUTX in human embryogenesis using various comparative genomics approaches. First, the function of LEUTX as a TF activating transcription was compared with other early human PRD-LIKE TFs using reporter assays with a minimal TAATCC-containing promoter; this showed that wild-type human *LEUTX* is not just a transcribed pseudogene. Then functionally critical regions and residues of Leutx proteins were defined by phylogenetic analyses. This revealed species variation among the specificity determining residues within the Leutx homeodomain. Further, conserved putative nine amino acid transcription activation domain (9aaTAD) motifs were identified in the C-terminus of Leutx. Next, we investigated the genetic variation of human *LEUTX* using population genetics, and did not find any putative deleterious homozygous mutations. Finally, we demonstrated that experimental point mutations in the homeodomain of human LEUTX lead to the loss of function as transcriptional activator towards a minimal TAATCC-containing promoter motif and an EEA-motif *in vitro*. Using homology modeling of the homeodomain structure we determined the likely mechanism of this loss-of-function. Our findings support the notion that human LEUTX acts as transcriptional activator with an important role for human embryogenesis.

## Results

### Human LEUTX activates a reporter containing TAATCC sequences in the promoter

We have previously shown that human LEUTX can activate a reporter construct containing the 36 bp EEA motif *in vitro*^15^. To focus only on PRD-LIKE TFs and to reduce the possibility of other TFs binding in this motif, we engineered a new luciferase reporter, containing only an 11 bp core region centered around the predicted PRD-LIKE binding site, TAATCC (see Materials and Methods). This consensus DNA-binding site has been defined in previous studies for DPRX, OTX2 and related PRD-LIKE TFs^14,26^.

**Figure 1:**
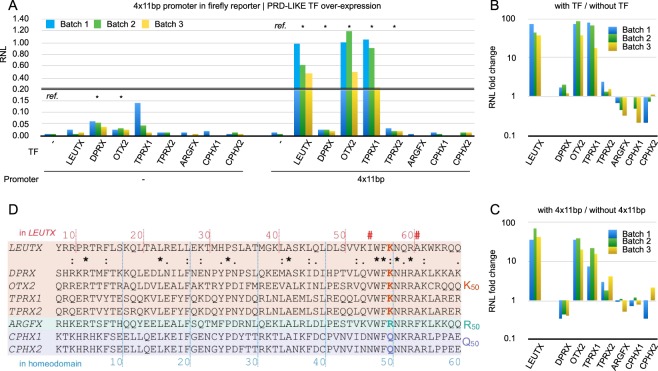
Transcriptional activation potential of PRD-LIKE TFs on the 11 bp TAATCC-containing promoter motif. (**A**) Renilla normalized luminescence (RNL) for control and 4 × 11 bp firefly reporter tested with different early PRD-LIKE TFs,. “-” at the bottom “Promoter” line denotes co-transfection with plain firefly reporter (pGL4.25), while “4 × 11 bp” denotes four copies of the 11 bp core motif (containing TAATCC) inserted into the promoter of the firefly reporter (pGL4.25-4 × 11 bp). “-” on the X-axis (TF) denotes no co-transfection of pFastBac-based TF over-expression vector, for co-transfection the TF names are indicated. Asterisks indicate statistically significant (p < 0.05, t-test) differences of RNL compared to the without TF control (labelled “ref.”) in each promoter. (**B**) RNL fold change calculated from the ratio of “with” and “without” TFs. Fold change values were calculated from (**A**), but only using experiments, where pGL4.25-4 × 11 bp was co-transfected. (**C**) RNL fold change calculated from the ratio of “with” and “without” 4 × 11 bp core promoter. Fold change values were calculated from (**A**), but only using experiments, where TF over-expression vectors were co-transfected. (**D**) Homeodomain amino acid sequences of the PRD-LIKE TFs. The numbering on the top is relative to the N-terminus of the full-length human LEUTX, and on the bottom is relative to the start of homeodomain. Period, colon, and asterisk indicate degree of conservation; the scorings are ≤0.5, >0.5 and <1, and =1 in the Gonnet PAM 250 matrix, respectively. “#” indicates position 47 and 54 of the homeodomain.

With this core 11 bp reporter we performed luciferase assays using DPRX, OTX2, TPRX1, TPRX2, ARGFX, CPHX1, CPHX2, and LEUTX (Fig. 1). LEUTX, OTX2, and TPRX1 activated the reporter strongly with statistical significance (p < 0.05 by t-test; Fig. 1A), while ARGFX, CPHX1, and CPHX2 induced no significant activation. The activation level of LEUTX is equivalent to that of OTX2, suggesting that it is not simply a transcribed pseudogene. DPRX and TPRX2 show weak, though statistical significant (p < 0.05 by t-test) activation when compared to the control lacking a TF vector (marked “ref”, Fig. 1A,B). However, when the transcriptional effect of DPRX is compared to the empty promoter vector without the 11 bp motif – similar to our previous publication^15^ – then DPRX shows a downregulation (Fig. 1A,C). The strength of the downregulation depends on the basal activity of the empty promoter vector (Fig. 1A). DPRX acting as a repressor is supported by the fact that a large set of genes in hESC is downregulated when DPRX is overexpressed^15,16^.

A comparison of the homeodomain sequences of the TFs explains some of the observed transactivation differences. DPRX, OTX2, TPRX1, TPRX2, and LEUTX are all PRD-LIKE TFs with a homeodomain of the K50 type, while ARGFX has R50, and CPHX1 and CPHX2 have Q50 (Fig. 1D). Thus, the latter three, because of the difference in the crucial specificity-determining residue 50, are not expected to bind to the TAATCC motif. The K50 type factors are all expected to bind to the motif, though only LEUTX, OTX2, and TPRX1 show strong activation. Previously we have shown that DPRX and TPRX2 act primarily as repressors when over-expressed in hESCs^15,16^. As we show below, LEUTX has conserved predicted transactivation domains in its C-terminus that are also present in ARGFX. These are not found in DPRX, which could explain why DPRX functions differently, i.e. as a repressor. How the different transactivation potentials between the related factors TPRX1 and TPRX2 arise needs further investigation. Thus, while the homeodomain binding-specificity determines which promoters are targeted, the transcriptional outcome as activator or repressor likely depends also on interactions with cofactors; these interactions are possibly mediated via motifs in N- or C-terminal flanking sequences.

### Phylogenetic distribution of Leutx genes and characterization of the Leutx domain

To examine the phylogenetic distribution and natural variation of residues and to identify evolutionarily conserved functional elements, we retrieved *Leutx* genes from Genbank, using blastp and tblastn (see Materials and Methods). Over 70% of the sequences retrieved and examined in depth required manual correction of their ORFs to optimally match the conserved domains as well as the canonical *Leutx* gene structure, which is comprised of three exons. One intron is located just upstream of the homeobox, while the second intron is located between codon 46 and 47 in the homeobox, a canonical splice site in PRD-LIKE TFs. *Leutx* sequences were retrieved from all four branches of Placentalia (Xenarthra, Afrotheria, Laurasiatheria, Euarchontoglires), but were not found in other animals (Additional file 1: Tables S1-S2), in agreement with research by Maeso *et al*.^22^. This suggested that *Leutx* has arisen in early Placentalia. A multiple sequence alignment (MSA) showed that Leutx proteins are comprised of two distinct domains, the homeodomain, and a conserved C-terminal region of about 110 amino acids, which we refer to as the Leutx domain (Additional File 1: Figs S1–S3).

In Glires, i.e. rodents and lagomorphs, numerous evolutionary changes are observed. On the one hand, several independent *Leutx* gene duplication events have given rise to tandem duplicated gene loci in several species (e.g., *O. cuniculus*, *C. porcellus*, *C. lanigera*) (Additional File 1: Table S1, Figs S1–S3 and S8–S11). On the other hand, intragenic repeats are seen between the homeodomain and the Leutx domain in some species. In hamsters (*C. griseus* and *M. auratus*) and desert wood rat (*N. lepida*) Leutx has a long repeat of 11 amino acids that separates the homeodomain from the Leutx domain, confirming the bipartite nature of Leutx. Two putative *Leutx* genes in prairie deer mouse (*P. maniculatus*) also encode repeats upstream of the Leutx domain, though the contigs are fragmentary and no homeobox was recovered (Additional file 1: Fig. S2). Rat and mouse, although reportedly lacking *Leutx*^22^, also have the 11 amino-acid repeat as well as the Leutx domain. However, no homeobox was recovered in the upstream region, although some repeats of the residues WFNQ, which are similar to WFQN in the homeodomain, were found (Additional File 1: Fig. S2). Further, exhaustive tblastn searches with several diverse Leutx homeodomains also failed to detect any Leutx-like homeodomains in mouse and rat. So far there is no evidence that either of these Muridae genes is transcribed, but the Leutx domain has been conserved for at least 7–12 Million years after the separation of rats and mice^27^. In Lagomorpha, the first part of the Leutx domain has apparently been lost: in rabbits the homeodomains are linked to the remainder of the Leutx domain via short polyproline repeats. In American pika, 5 repeats of about 40 residues in length link the homeodomain and the Leutx domain; a conserved PWAS sequence element of the Leutx domain is part of this repeat (Additional File 1: Fig. S2). Overall, the Leutx genomic region seems to be subject to instability in Glires, given the various gene duplications and intragenic repeats observed.

Few sequences were retrieved from Afrotheria at present. The sequence from cape elephant shrew (*E. edwardii*) is a pseudogene (Additional file 1: Table S1). In elephant, there are two *Leutx* genes as also noted earlier^22^. We note that one of these homeodomains is unusual, having an asparagine residue at position 50 of the homeodomain instead of a lysine (Additional File 1: Fig. S1).

### Functional elements in the Leutx domain

The C-terminal region of the Leutx domain is the most conserved section of that domain (Additional File 1: Fig. S3). Secondary structure prediction of the multiple aligned Leutx domains showed only two to three short regions predicted to be helical, while the remainder is mostly unstructured and solvent exposed (Additional File 1: Fig. S4). This suggests that the LEUTX domain overall is a flexible domain with limited globular structure. We noticed numerous proline, serine, threonine, and acidic residues in the Leutx domain. Motifs enriched in these residues are referred to as “PEST” sequences and are involved in rapid protein degradation^28,29^. Analysis of PEST sequences showed that they tend to be enriched in disordered protein regions, lacking secondary structure^30,31^, which agrees with our secondary structure predictions. We examined several LEUTX protein sequences using the computer program epestfind and found that there are usually three to four low scoring PEST regions in the Leutx domain, e.g., in human LEUTX (Additional File 1: Fig. S5).

Motifs containing acidic and hydrophobic residues have been shown to function as transcription activation domains. In particular, the 9aaTAD sequence has been well studied^32,33^. We tested a number of different Leutx protein sequences with the 9aaTAD web server (Additional File 1: Fig. S6). Two regions in the C-terminus of Leutx were consistently predicted to be potential 9aaTAD motifs. These regions lie in the most conserved part, where the two highly conserved tyrosine residues are located, and overlap with the regions predicted to be alpha helical.

Some weak similarity within the C-terminal region has also been observed with Argfx and Crx^22^. We independently noticed that Argfx proteins also have a conserved C-terminal region that shares sequence similarity with the Leutx domain (Additional File 1: Fig. S7). This similarity is highest in the region where the 9aaTAD motifs are predicted. Analysis of human ARGFX also reveals putative 9aaTAD motifs in this area (Additional File 1: Fig. S7). Furthermore, the ARGFX C-terminal region is also rich in PEST residues (data not shown). We conclude that the C-terminal regions of Leutx, Argfx, and possibly other protein families that have similar features (such as Crx, which we have not examined further) have been subject to evolutionary selection, most likely due to the putative transactivation domains. Furthermore, the presence of putative PEST motifs suggested that these proteins are rapidly degraded, which would be consistent with the early, transient expression observed in the human embryo.

### Phylogenetic analysis of Leutx sequences

**Figure 2:**
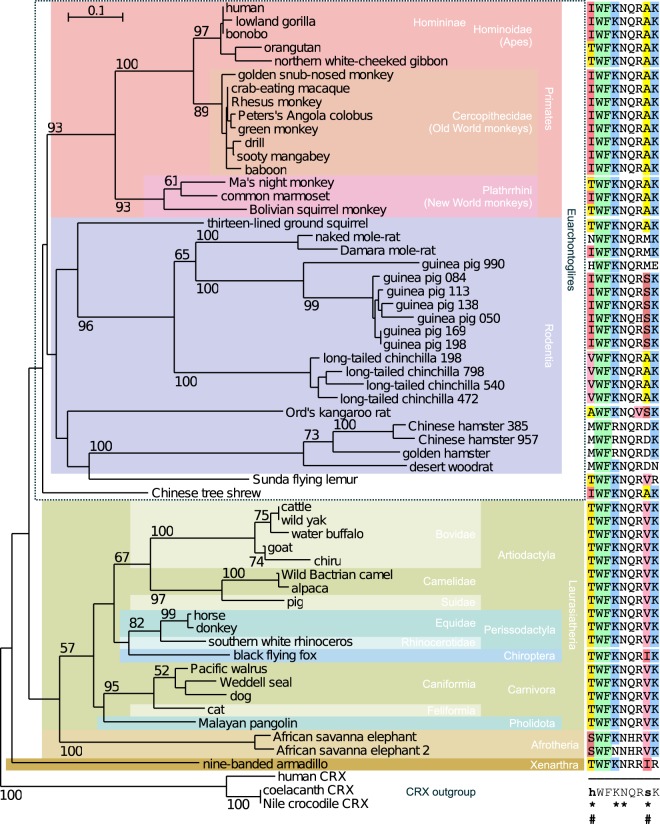
Phylogenetic tree of Leutx protein sequences of eutherian species, and variation of the specificity determining residues. The phylogenetic tree was constructed using Neighbour joining (see Materials and Methods). Three Crx homeodomain sequences (bottom) were used as outgroup, since the *Leutx* gene is likely derived from a *Crx gene*^22^. Right panel: Alignment of amino acid sequences in the recognition helix, spanning residues 47 to 55 of homeodomain (54 to 62 of human LEUTX). Consensus (bottom right) is set at the 90% level, and the notation is based on MView^83^ with “*” indicating the specificity determining residues. “#” indicates position 47 and 54 of the homeodomain (see also Fig. 1).

Phylogenetic analysis reveals that the Leutx homeodomain as well as the Leutx domain co-evolve, and that the cladogram reflects the placental systematics reasonably well (Fig. 2 and Additional File 1: Figs S8–S10). However, we noted that the branch lengths are much longer for Leutx homeodomains, than, for example, for Crx homeodomains. This higher level of divergence is also exemplified by the fact that Leutx homeodomains can be quite divergent from each other (Additional File 1: Table S3). For example, even within primates, the homeodomain of human LEUTX is only 67% identical to that of the Bolivian squirrel monkey (*S. boliviensis*), while the Crx homeodomain of Latimeria is 87% identical to that of human CRX. Within Laurasiatheria, homeodomain sequence identity can be as low as 55%, and within Glires even less than 40%. This indicates that *Leutx* genes are evolving much faster than most other homeobox genes, which are well conserved in evolution^3^.

In Laurasiatheria, the phylogenetic tree generated from the homeodomain shows a large overlap with recent phylogenetic studies (Fig. 2 and Additional File 1: Fig. S8)^34^. The Even-toed (Artiodactyla) and Odd-toed ungulates (Perissodactyla) as well as Carnivora are well resolved with good bootstrap support. Furthermore, bats (*P. alecto*, black flying fox) are found among Laurasiatherias, as the more recent phylogenetic studies showed^34,35^. This suggests, despite the limited information content of the 60 residues of a homeodomain, that Leutx evolution coincides with the phylogenetic evolution of Placentalia.

In Euarchontoglires the situation is more complex. In primates, we also observe well-separated clades for the new world monkeys (Platyrrhini), old world monkeys (Cercopithecidae) and Hominoidea (Fig. 2 and Additional file 1: Fig. S8). Recent results for the Chinese tree shrew suggested that it is more closely related to primates than to rodents^36^, but our results do not clearly resolve this issue (Fig. 2 and Additional File 1: Figs S9 and S10).

Within the Glires clade we notice a more dramatic sequence divergence. While there is still an overall agreement with rodent and lagomorph evolution^27,37^, branch lengths are longer (Fig. 2 and Additional File 1: Fig. S10). In Ord’s kangaroo rat (*D. ordii*) the spliced copy of *Leutx* shows noticeable changes in the MSA within the Leutx domain and long branch lengths within the phylogenetic tree (Additional File 1: Figs S1,S2, and Additional File 1: Fig. S10). In Caviida, we found multiple tandem gene duplications containing introns. Thus, one might expect most of them to be transcribed. It is also noteworthy that the gene duplications in guinea pig (*C. porcellus*) and chinchilla (*C. lanigera*) occurred independently in the two lineages (Additional File 1: Fig. S11). In the common ancestor of Cricetidae and Muridae^27^, the 11 amino acid repeat located between the homeodomain and the Leutx domain seems to have arisen for the first time. In the Muridae family, the homeodomain subsequently seems to have been lost. But also in the Cricetidae family the homeodomain changed, losing the basic arginine residues at position 2/3, and 5 of the homeodomain that contact the minor groove of the DNA. In the Cricetinae subfamily (hamsters), the homeodomain underwent a further critical change, with K50 changing to R50. Thus, in the Cricetidae family, the Leutx homeodomain has undergone substantial changes, suggesting a dramatic shift in specificity.

In summary, the evolutionary conservation of *Leutx* in many Placentalia combined with the early expression suggests a critical role in embryonic development. Furthermore, the presence of independent putative reverse-transcribed pseudogenes in several species – although we did not conduct an exhaustive search – also suggests that *Leutx* genes are active in the germ line or early embryo. While *Leutx* does evolve faster than other homeobox genes, it is well conserved in Laurasiatheria and Primates; the phylogeny of the latter is similar to the expansion of Alu elements in primate genomes^38,39^.

### Neither common nor homozygotic missense mutations are found in the recognition helix of human LEUTX

**Figure 3:**
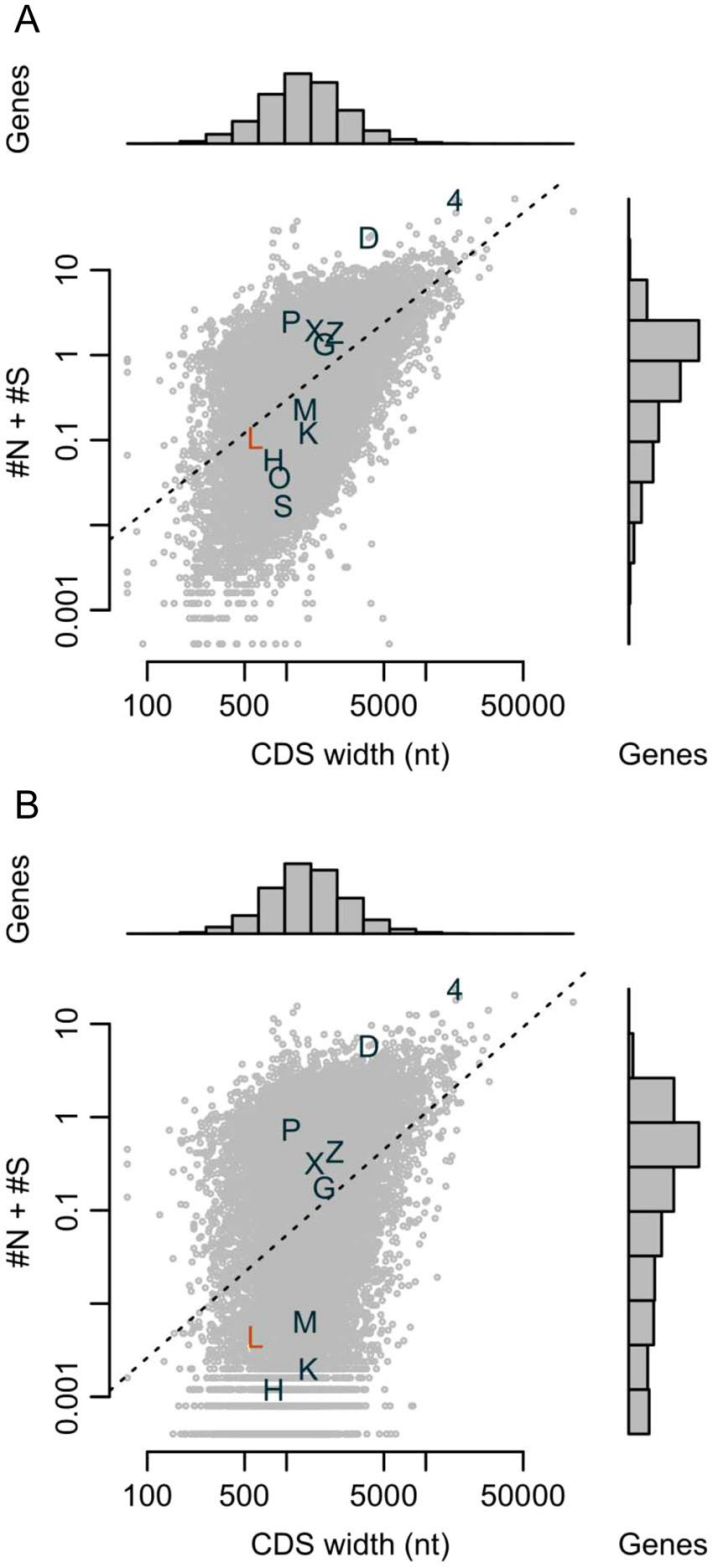
Genomic homogeneity of human *LEUTX* and other protein coding genes. Both scatter plots represent the average number of both, nonsynonymous (#N) and synonymous (#S), substitutions per individual (y-axis) in the phase 3 results of IGSR/1000G database^40^; (**A**) heterogeneous and homogenous mutations, and (**B**) homogenous mutations only. The dotted line is a regression between the CDS width (x-axis) and the average numbers of substitutions (y-axis). Label “L” is *LEUTX*. “4” is *MUC4*, which had the most non-synonymous mutations among #N > #S genes, and “D” is *DPSS*, which had the most synonymous mutations among #N < #S genes. The other labels G, H, K, M, O, P, S, X and Z are *GLIS1*, *HHEX*, *KLF4*, *MYC*, *OTX2*, *POU5F1*, *SOX2*, *HLX* and *ZNF366*, respectively.

Based on the 1000 Genome Samples^40^, one in every 10 individuals has one heterozygous mutation within the coding region of *LEUTX*; homozygotic mutations are even less frequent in human as a taxon (Fig. 3). Moreover, the mutation frequencies in *LEUTX* are lower than the average of all human protein coding genes, although the length of the coding region is short, suggesting that *LEUTX* is relatively constrained in human individuals. In contrast, the higher mutation rates of *LEUTX* compared to TFs conserved across the bilaterian divide, such as *OTX2* or *SOX2*, may be due to less mutational pressure in somatic cells, because *LEUTX* is expressed only during a short developmental time window unlike *OTX2* and *SOX2*; only germ line mutations in *LEUTX* can cause any changes in phenotype.

For further intra-species comparisons, we investigated seven available human genotype resources: phase 3 of 1000 Genome^40^, ExAC release 0.3^41^, NHLBI Exome Sequencing Project Genome of Netherlands^42^, The Human Genetic Variation Database (Japanese)^43^, Gnomad^41^, and the deCODE Icelanders database (made available to us by Dr. Kári Stefánsson and his colleagues). Altogether we found 116 variants in *LEUTX* (Additional file 1: Table S4). Four of the 116 variants (p.R9H, p.S93P, p.A116H and p.T177P) are common (~ max allele frequency >1%) missense variants in any cohort (Additional File 1, Fig. S1, blue arrows). Also, two of the 116 variants (p.R9H and p.T177P) are encountered as homozygotes in the Gnomad cohort. However, all mutated amino acids are evolutionarily accepted in the other species (Additional File 1, Fig. S1), suggesting permissive changes. The other variants are neither common, nor are there any rare allele homozygotes. We examined the 116 alleles considering phylogenetic conservation and possible effects on the homeodomain and the 9aaTAD. We expect that 24 of these variants (missense, splice donor, frameshift, and nonsense mutations) could impair the function of LEUTX (Additional file 1: Table S4, marked in yellow) and hence would not be expected to propagate, possibly explaining their low allelic frequency. One of these 24 mutations, an individual in the 1000 Genome data, possessed a heterozygotic missense mutation at a DNA specificity-determining residue within the recognition helix (p.A61V, which is at position 54 in the homeodomain; sample ID HG02597). As we show below experimentally, this change from alanine to valine leads to a loss of function. This rare allele has not been transmitted to the son (sample ID HG02599). Therefore, the father’s mutation might be somatic, or might not have been inherited, because it is deleterious.

We also examined the Neanderthal and Denisova genome sequences^44^, and found one change in the Leutx domain of the Denisova genome that results in an amino acid change (G149V; marked in green Additional File 1, Fig. S1). That position is not highly conserved, but no valine has been found there so far, so the functional consequences are unclear.

### I47, K50, N51, A54, and R58 of the LEUTX homeodomain are specificity determining residues

**Figure 4:**
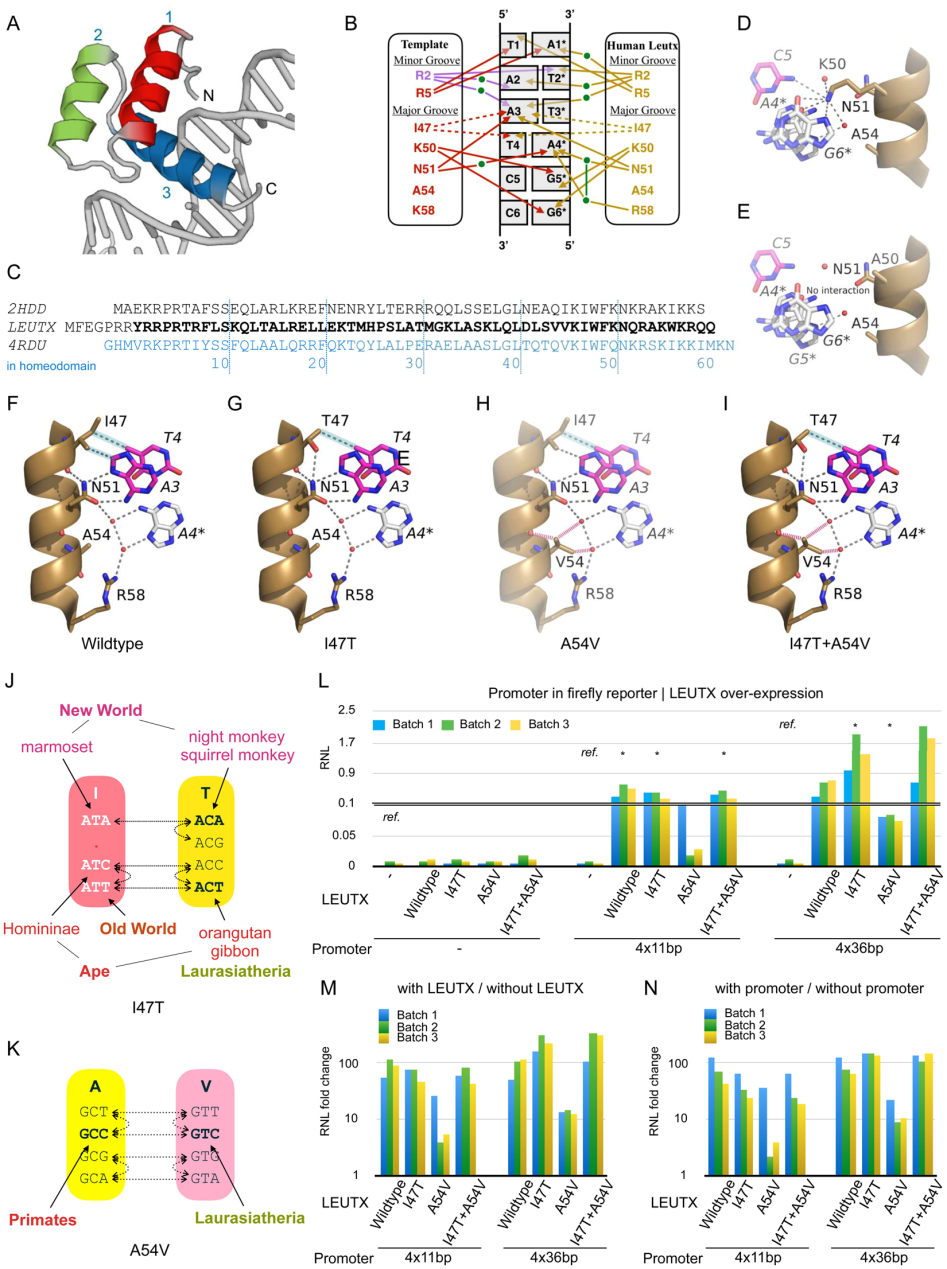
Modelling of homeodomain-DNA interactions in human LEUTX and transactivation potential of point mutations at positions 47 and 54. (**A**) Structure of the Q50K Engrailed homeodomain-DNA complex (PDB:2HDD, chain A), which was used as template for modelling the human LEUTX 3D structure. Alpha helices 1, 2 and 3 are coloured red, green, and blue (=recognition helix), respectively. (**B**) Scheme of protein-DNA interactions and relative positions in the sequences (see alignment). The left panel shows the homeodomain residues of Engrailed (red; 2HDD) and DLX5 (purple; 4RDU), while the right panel shows those of human LEUTX (yellow). Interactions with the DNA are marked with arrows - hydrogen bonds, solid; hydrophobic interactions, dashed - in the corresponding colours. Green dots represent water molecules. The nucleotides are numbered from 1 to 6 for the motif 5′-TAATCC-3′, and asterisks denote the antisense strand. (**C**) Amino acid sequences of human LEUTX and the templates used for the modelling. Due to missing structural data in the PDB entry 2HDD (Engrailed) at the N-terminus, the structure of human DLX5 (4RDU, chain D) was used for modelling residues 1–4 of the LEUTX homeodomain. (**D**–**I**) Structural models of wild-type and mutated human LEUTX homeodomains. Water molecules are shown as red spheres; hydrogen bonds as dotted grey lines; hydrophobic interactions as dotted grey lines highlighted in cyan; and non-favourable interactions as dashed red lines. (**D**) Close-up view around residue K50; lysine can form many possible interactions and assumes two conformations in the 2HDD X-ray structure. (**E**) Structural model with the K50A mutation; alanine is unable to interact with DNA like K50. (**F**) Wild type LEUTX homeodomain showing interactions of the specificity-determining residues I47, N51, A54, and R58. (**G**), Model of the I47T mutation (**H**), Model of the loss of function mutation A54V (**I**) Double mutation of I47T and A54V, which restores function. **(J**,**K**) Residue and codon usage comparison between primates and Laurasiatheria at position 47 (**J**) and 54 (**K**) of the LEUTX homeodomain. Dotted lines represent possible paths for transitions, which are more frequent than transversions, between two amino acids. (L-N) RNL for different promoter reporter constructs and different LEUTX protein expression vectors. “-”, “4 × 11 bp”, “4 × 36 bp”at the bottom “Promoter” line denote co-transfection of plain firefly reporter (pGL4.25), “4 × 11 bp” reporter, or “4 × 36 bp” reporter, respectively. X-axis labels (marked LEUTX) indicate the different LEUTX TFs used: “-”: no co-transfection of pFastBac-based TF over-expression vector; “Wild type”: co-transfection of wild-type human LEUTX; I47T, A54V, and I47T + A54V: cotransfection of mutant LEUTX proteins. Asterisks indicate statistically significant (p < 0.05, t-test) differences of RNL compared to the control without TF (“-”, labelled also by “ref.”) for each promoter construct. Batch 1 to 3 represent the three experimental replicates. (**M**) RNL fold change derived from “with” and “without” LEUTX protein expression constructs. Fold change values were calculated from (**L**), where either 4 × 11 bp or 4 × 36 bp was co-transfected. (**N**) RNL fold change derived from “with” and “without” promoter reporter constructs. Fold change values were calculated from (**L**), where TF over-expression vector was co-transfected.

To identify critical residues for protein-DNA interactions we wanted to examine the molecular structure of human LEUTX. Since no experimental structures are available yet, we made a 3D structural model of its homeodomain using the engrailed homeodomain of *Drosophila melanogaster* (PDB ID: 2HDD, chain A^13^) as template (Fig. 4A). Because the template was missing coordinates for the first four residues of the homeodomain, the N-terminal region of the LEUTX model was built using the X-ray structure of the human DLX5 homeodomain (PDB ID: 4RDU, chain D; Fig. 4B,C; see also Materials and Methods). The high sequence similarity with the binding residues present in the template structure allowed us to evaluate the possible interactions that may take place in the LEUTX homeodomain-TAATCC complex and are likely to determine the promoter sequence specificity (Fig. 4B).

In the modeled homeodomain of human LEUTX (Fig. 4B,D and F), the residues I47, K50, N51, and A54 in the third alpha helix (residues 42–58) are conserved in the template X-ray structure of the engrailed homeodomain, and residue R58 shares similar physicochemical properties with K58 of the template structure; all these residues bind to the TAATCC motif. In human LEUTX, K50 would be hydrogen-bonded to guanine G5* and G6*, complementary to the cytosines of the TAATCC motif. We recently reported^16^ that the K50A mutation (K57A in full-length human LEUTX) abolished the reporter activity in a luciferase reporter assay, in agreement with the loss of hydrogen bonds mediated by the K50 residue of the native protein (Fig. 4E).

### Conserved N-terminal arginines at position 2/3 and 5 recognize the DNA minor groove

Although the third helix contains several residues giving specificity to DNA recognition, structural data also suggested that other residues play a role in DNA-binding. The guanidinium group of R5 is required for the recognition of thymine^5^ and in the human LEUTX homeodomain R5 is positioned to form hydrogen bonds with the first thymine base in the TAATCC motif and via a water molecule with adenine on the opposite strand that base pairs with the thymine base itself (Fig. 4B). Another nearby arginine residue, R2 of LEUTX, matching R138 of the 4RDU structure template (chain D), would form hydrogen bonds possibly via water molecules – as seen in the 4RDU template structure – to the two adenine bases in the TAATCC motif and interact with thymine of the opposite strand that base pairs with the first adenine base in the motif (Fig. 4B). Arginine residues at position 2, 3 and 5 are well conserved – 84%, 77% and 93% respectively (Additional File 1: Figs S1,S3) – as in homeodomains in general^3^. In the case of the *Drosophila* Hox protein Sex combs reduced (Scr) two arginine residues make contact in the minor groove, and the shape of the DNA, i.e. the width of the minor groove, plays a role^45^. More specifically, only two arginines – either R2 or R3, and always R5 – can simultaneously be involved in contacts with the minor groove of DNA. For example, in the human PAIRED homeodomain protein PAX3 (PDB ID: 3CMY, chain A^46^) and the *D. melanogaster* paired protein (PDB ID: 1FJL; chain A^47^) the residues in contact with the minor groove are R2 and R5, whereas in the *D. melanogaster* aristaless homeodomain (PDB ID: 3A01, chain B; PDB ID: 3LNQ, chain A^48^) and the even-skipped homeodomain (PDB ID: 1JGG, chain A^49^), R3 and R5 are the reported contact residues. Consistently, the RefSeq LEUTX sequence (GenBank: NP_001137304.1), which lacks the N-terminal part of the homeodomain, had less transcriptional activity than full-length LEUTX^15^.

### Mutation of A54 into V in human LEUTX abolishes TF function while a second compensatory mutation in I47 restores function

Detailed inspection of the recognition helix residues in the Leutx phylogeny revealed that two of the five specificity-determining residues in the third helix exhibited differences, especially between primates and laurasiatherians. One key residue is isoleucine at position 47 (Fig. 2); while most examined primates have I47, in several instances (orangutan, gibbon, night monkey, and squirrel monkey) threonine is found at that position. In laurasiatherians the residue is threonine instead of I47. The second variant key residue is at position 54; it is conserved as alanine among primates, however the residue is predominantly valine in laurasiatherians (Fig. 2).

By making intra-species and inter-species comparisons of the LEUTX recognition helix, we noticed that there is no I47-V54 residue combination (Figs 2 and 4F–I), although each of these residues can easily be changed by one transition mutation; position 47 has more variability than 54 among primates, but both are highly constrained in Laurasiatheria (Fig. 4J,K). Why does the I47-V54 mutant combination not appear, although it is readily reachable evolutionarily? We hypothesized that a I47-A54V combination might be lethal or deleterious, because DNA-binding would be compromised. To confirm this hypothesis, we studied the functionalities of the four variants (2 variants in 2 positions) by using the luciferase reporter assay (Fig. 4L–N). As expected, the single mutation of A54V in the presence of I47, which is not encountered evolutionarily, strongly reduced the activating function on the TAATCC-containing 4 × 11 bp promoter (Fig. 4M; p < 0.05 by t-test, RNL fold change compared to wild type). Although the single mutation of A54V activated the 36 bp EEA-motif promoter also, the activity was less than the other mutants as well as the 4 × 11 bp promoter. The single mutation I47T and the double mutation I47T-A54V showed significant activity on the 4 × 11 bp promoter, indicating that primate Leutx with I47T and laurasiatherian Leutx can still bind TAATCC. The increased activity of the I47T and the I47T-A54V variants on the EEA-motif promoter might be due to increased contributions of other TFs, which are expressed in the HEK293 host cells.

We then investigated the alterations based on the structural models. The mutation of I47 (found in some non-human primates, Fig. 4G) to a more compact but polar T47 residue provides extra potential for making stabilizing hydrogen bonds. I47 makes two weak hydrophobic interactions with nucleotides T4 and A3. In the model of human LEUTX, where the I47T mutation was introduced, the methyl group of T47 would be able to interact with the methyl group of T4, maintaining one of the hydrophobic interactions seen in the wild-type complex. The side-chain hydroxyl oxygen atom of T47 is ideally positioned to form a strong hydrogen bond to the amide group of the N51 side chain and thus help to stabilize and rigidify this region and its interactions with the TAATCC motif and with two water molecules that are key to base recognition as seen in the 2HDD structure.

The A54V mutation (Fig. 4H) leads to a bulkier hydrophobic side chain that significantly reduces binding to the DNA motif. In the modelled complex with V54, the bulkier side chain of valine would likely interfere with the location of water molecules that form the network of hydrogen bonds linking N51 to R58 and that are critical for binding the adenine base A4* of the antisense strand of the TAATCC motif. Interference with this water mediated hydrogen-bonding network is the most obvious cause of the reduced binding of the A54V mutant.

In the double mutant (I47T and A54V; Fig. 4I; found in laurasiatherians), experimental binding to the motif is restored and is similar to that seen in the I47T mutation alone. This may be explained by the key role of the side chain of N51 coupled with its enhanced stabilization due to the hydrogen bond from T47. This stabilization of N51 may be sufficient in itself to compensate for any disruption in the water-mediated hydrogen bonding network due to the A54V mutation. It is likely that this stabilization of N51 by T47 also allows the water mediated network to persist despite the presence of the disruptive influence of the A54V mutation, maintaining these indirect interactions between the homeodomain and the adenine base A4*.

## Discussion

We applied several comparative genomics and experimental approaches to assess the importance of human *LEUTX* for developmental processes. To examine the evolution of the *Leutx* gene family, we retrieved *Leutx* sequences from Genbank. The presence of *Leutx* in Xenarthra (armadillo), Afrotheria (elephant, and pseudogenes in Cape elephant shrew), Laurasiatheria, and Euarchontoglires, but their lack in Marsupialia and Monotremata, indicates that *Leutx* originated after the divergence of Placentalia from Marsupialia as also suggested by^22^. We observed that Leutx proteins are conserved among most placental clades, but do exhibit evolutionary variation in the rodent and lagomorph lineages, including loss of the homeodomain in rats and mice, which clearly represents a secondary loss. Within human individuals (as intra-species comparison) *LEUTX* is relatively uniformly conserved, and deleterious homozygotic mutations, in particular also missense mutations within the recognition helix of the homeodomain were completely lacking. Potential deleterious alleles are only found heterozygously at low frequency.

The evolutionary and structural comparisons allowed us to identify potentially important DNA-binding residue combinations. Although the importance of positions 47 and 54 for DNA-binding and activity using mutational analysis has already previously been established^6–10^, those studies were not conducted with PRD-LIKE TFs. We found that the mutation I47T in the homeodomain of human LEUTX has little effect on LEUTX activity – T47 is also found in several primate sequences –, while the mutation A54V abolishes activity. In contrast, V54 is commonly found in Laurasiatheria, indicating it is functional, but there it is rigidly accompanied by a threonine at position 47. Our experiments here show that a compensatory change in human LEUTX (I47T, in combination with A54V) restores activity again in a luciferase assay (Fig. 4L–N). Chu *et al*.^6^ showed already that there is a correlation of residues 47 and 54 with respect to binding a specific base. Our experiments and structural models confirm this important interplay between these two positions for function. Interdependence and interference between residues 50 and 54 for DNA-binding has also been observed, e.g., the A54M mutation in combination with K50 in Goosecoid is not DNA-binding^50^. However, we do note that mole rats and a guinea pig sequence (Fig. 2), as well as the third homeodomain of the *C. elegans* CEH-91^51^ have this combination. Although the effects on DNA affinity and specificity are not known, the evolutionary conservation of K50 M54 in mole rats at least suggests it is functional.

Quite a number of amino acid changes have occurred in the homeodomain of primate Leutx during evolution, but residues critical for structure and DNA-binding have been conserved. For example, A54V has been avoided, despite the fact that this position can easily be mutated by a single transition. If another gene could complement for the missing function of *LEUTX*, or if *LEUTX* did not have an important role in preimplantation development, then such an A54V loss-of-function variant might have arisen during the evolution of primate species or in human populations. However, such a variant is presently not observed, supporting the notion that *LEUTX* plays an important role in the developmental processes of the early embryo as previously suggested^15^. It is perhaps counterintuitive why an apparently essential gene can be lost in some species. However, empirical observation indicates that while the mid-embryonic phylotypic period is most conserved in evolution, early embryogenesis and late developmental stages are subject to more evolutionary flexibility^52–54^. A prime example for early divergence in evolution is the fly gene *bicoid*, which was derived from a Hox cluster gene and “inserted” itself into the regulatory network of the zygote, where it is setting up the fundamental antero-posterior axis^55–57^. More dramatic losses of “essential” genes have been observed in *C. elegans*, where the Hox cluster has degenerated^58^, and where the hedgehog signalling gene has been lost^59,60^.

Embryonic implantation differs among mammals, being either superficial or interstitial. The basic mode of implantation is superficial, and the interstitial mode developed multiple times independently in mammalian lineages, including Muridae and Humanoids^61^. Hence, it does not correlate with our observed pattern of *Leutx* sequence conservation in primates and laurasiatherians, versus the dramatic evolutionary changes of *Leutx* in Glires. Nevertheless, given that interstitial implantation has developed several times de novo, it cannot be excluded that part of the regulatory cascade involved in the implantation mode involved the change or loss of *Leutx* in the Glires lineage.

A much more obvious correlation of the phylogenetic pattern of Leutx sequence conservation is with gestation times. It is noteworthy that species, where the homeodomain has undergone substantial changes, or was completely lost, have very fast gestation times, often less than 25 days (Additional file 1: Table S2). These species have apparently been under strong evolutionary selection to develop short gestation times. This may have resulted in necessary adaptive changes in the molecular regulatory networks during early embryonic development, including *Leutx*. Other early PRD-LIKE homeobox genes, such as *Argfx*, *Dprx*, and *Pargfx* have also been lost in the mouse lineage^22^, indicating that not just a single gene, but a network was modified or lost. Indeed, we previously observed that human LEUTX was able to activate only about 25% of human EGA genes^15^, an indication that other TFs play a role in activating the complete set of EGA genes. Support for such a shift in regulatory networks comes from a recent analysis, which shows that the Obox and Croxs homeobox genes have substantially expanded in mouse and seem to have replaced the function of the missing Argfx^62^. Further studies are needed though to understand how combinations of PRD-LIKE and other TFs as well as their cofactors regulate embryonic events, including gestation periods.

One worst-case scenario from the evolutionary perspective is infertile interspecies offspring (such as mule), resulting in the spending of parental resources toward no next-generation reproduction. The earlier in development an illegitimate embryo is eliminated, the better for reproductive fitness. The early elimination of cross-species embryos would be advantageous for both species, promoting rapid evolution of critical EGA mechanisms. *Leutx* is evolving much faster than other homeobox genes, most of which are well conserved in evolution across Bilateria^3^. Intriguingly, the Leutx phylogeny correlates with the expansion of Alu elements in primate genomes^38,39^, and, the 36 bp DNA element containing the TAATCC motif displays similarity to Alu elements^18^. It is tempting to speculate that any essential gene for EGA might be rapidly evolving in order to build strong inter-species barriers between closely related species. In this context, we note that another rapidly evolving PRD-LIKE homeobox gene, *odysseus*, has been shown to be involved in speciation in *Drosophila*^63^.

Taken together, we have applied a combination of inter-species comparative analysis, intra-species human genome resources, structural predictions, and experimental testing of critical amino acid substitutions to address the hypothesis that *LEUTX* plays a role for human embryogenesis as suggested earlier^15^. All evidence supports this notion; among a vast number of human genomes assessed, only one individual with a heterozygous variant was noted. Further, in Placentalia Leutx is in general well conserved, only in Glires do we observe rapid changes and degeneration of the homeodomain. This could be explained by special evolutionary pressure for rapid gestation times. We conclude that human *LEUTX* is highly constrained and is likely to play an important role in human embryogenesis, but further studies are needed to confirm the notion.

## Methods

### Construction of expression vectors and mutated *LEUTX* expression constructs

In order to overexpress ARGFX, CPHX1, CPHX2, DPRX, LEUTX, OTX2, TPRX1 and TPRX2 protein in human cells, the respective ORFs were cloned into a modified pFastBac expression vector CMVe.EF1α.eGFP-WPRE as described in^15,16,18^. Accession numbers of the sequences are given in Additional file 1: Table S5.

In order to mutate two key amino acids (I47T and A54V) of human *LEUTX* in the pFastBac vector a QuikChange II site-directed mutagenesis kit (Agilent, Santa Clara, CA) was used according to manufacturer’s instructions with primers described in Additional file 1: Table S6. To mutate both residues simultaneously, I47T and A54V, the construct with mutation I47T was further mutated using primers for the mutation A54V. All constructs were verified by Sanger sequencing.

### Construction of luciferase reporter vectors and luciferase reporter assay

In order to study the effect of the PRD-LIKE TFs on the PRD-LIKE binding site contained in the 36 bp EEA-motif^18^, two reporter constructs were designed: a 216 bp construct comprising four EEA-motifs [CAGCCTCCCAAAGTGCTGGGATTACAGGCATGAGCC] in tandem with intervening restriction sites, and a PCR-amplified 131 bp construct comprising four repeats of a shorter 11 bp core motif [CTGGGATTACA] in tandem with intervening restriction sites.

The “36 bp” construct was fully synthesized (Eurofins, Ebersberg, Germany) as described in^15^, and the “11 bp” construct (Additional File 1: Fig. S12) was generated by PCR amplification using primers 11_bp_Fw and 11_bp_Rv (Additional file 1: Table S6) as follows: each primer contained two copies of the 11 bp motif with intervening restriction sites and an SfiI restriction site at one end enabling the ligation to the vector backbone, and a BsaI restriction site at the other end enabling the ligation of the two amplified fragments. The two fragments were amplified using primers 11_bp_Fw and pGL4_F, or 11_bp_Rv and pGL4_Rv, respectively (Additional file 1: Table S6), using Phusion DNA polymerase (Thermo Scientific) according to the manufacturer’s instructions. pGL4.25 [luc2CP/minP] firefly luciferase reporter vector (Promega, Madison, WI) was used as a template. The products were amplified using PCR as follows: 98 °C for 3 min; 35 cycles of 98 °C for 30 s, 65 °C for 30 s, 72 °C for 30 s; final extension 72 °C for 10 min. The amplified fragments were digested with SfiI and BsaI, and the pGL4.25 vector was correspondingly digested with SfiI. The digested fragments were purified from an agarose gel using NucleoSpin Gel & PCR Clean-up kit (Macherey-Nagel) and ligated using T4 DNA ligase (Thermo Scientific) according to the manufacturer’s protocol.

The reporter constructs or empty renilla luciferase vector pGL4.74 were co-transfected with expression vectors for each TF one-at-a-time into HEK293 human embryonic kidney cells (ATCC, Middlesex, UK). The cells were seeded on 48-well plates in Dulbecco’s modified Eagle medium containing 4.5 g l^−1^ glucose, sodium pyruvate and sodium bicarbonate (Sigma-Aldrich) and supplemented with 10% FBS and 1 x GlutaMAX (both from Gibco). Cells were grown overnight at 37 °C in 5% CO_2_ and subsequently transfected with different combinations of luciferase vector constructs, pFastBac vector constructs and Renilla luciferase vector pGL4.74. The concentrations of single constructs were as follows: Luciferase vector 100 ng per well, pFastBac vector 100 ng per well and Renilla luciferase vector 5 ng per well. To estimate the optimal pFastBac concentration for TF expression a titration was performed, see Additional File 1: Fig. S13. The transfections were performed using FuGENE HD Transfection reagent (Promega) using 1 µL per well according to the manufacturer’s instructions. Cells were incubated at 37 °C in 5% CO_2_, harvested 24 h after transfection and subjected to Dual luciferase assay (Promega) according to the manufacturer’s protocol; the luciferase reporter assays were performed in 3 biological replicates. Luciferase signals were measured using a FLUOstar Omega microplate reader (BMG Labtech, Ortenberg, Germany). Renilla normalized luminescence (RNL) is firefly luminescence level divided by renilla luminescence level.

### Bioinformatic and phylogenetic analyses

LEUTX sequences were retrieved from NCBI using blastp of the non-redundant databases, and, for more selective searches, using tblastn of specific WGS, EST or REFSEQ databases^64^. Retrieved records were stored in a database, and then manually curated. Retrieved sequences with their accession numbers are shown in Additional file 1: Table S1. Gene identifiers were automatically generated from extracted database information. Species abbreviations and prefixes used in the figures are shown in Additional file 1: Table S2. MSAs were carried out using MUSCLE^65^ and SEAVIEW (Clustalo option)^66^. Phylogenetic analyses were carried out using the Neighbor joining variant BioNJ^67^ and the Maximum Likelihood version PhyML^68^ as implemented in SEAVIEW (BioNJ option, and PhyML, model LG default option)^66^. Clustal_X was also used for MSA handling^69^. Based on the MSAs numerous sequences contained obvious errors in their ORF predictions. Corrections were applied using sequence similarity (the homeodomain and the Leutx domain) as well as genomic synteny between orthologs. The results show that the canonical gene structure for *Leutx* follows that found in humans: a short first exon encoding often only 2 residues with a splice donor site in phase 1; an intron of several kb, a second exon that starts 14 nucleotides upstream of the homeobox, and encodes the majority of the homeodomain; a second, shorter intron (often around 1 kb) between codons for residues 46 and 47 of the homeobox in phase 0, which is a typical splice position for PRD-LIKE homeobox genes; the third exon, encoding the reminder of the homeodomain and the Leutx domain up to the C-terminus^15^. Additional tools used to aid in correct ORF identification were the Human Splicing Finder^70^, Fgenesh+(http://www.softberry.com)^71^, and the Java software “Sequence Analysis” (http://informagen.com/SA/). In some instances, closely related sequences were compared at the genomic sequence level to identify orthologous exons, using the dot matrix utilities dotlet (http://myhits.isb-sib.ch/cgi-bin/dotlet)^72^, and Gepard^73^. While the best efforts were made to obtain the most likely ORFs, sequencing errors, assembly errors, the fragmentary nature of contigs, or missing sequencing information obviously affect the predictions. Pseudogene predictions were based on either the lack of introns (a retrotransposed gene), or on multiple errors (stop codons, and/or frame shifts) that affect the most likely ORF, the most likely ORF being defined as the one having the best sequence similarity to orthologous sequences in other species. Prediction of the short N-terminal exons are subject to more uncertainties.

The Pairwise distance matrix was generated using MegAlign Pro in DNASTAR (DNASTAR, Inc.). The Protein Logo was generated using LogoBar^74^. Secondary structure prediction was carried out using PredictProtein (PROFphd, www.predictprotein.org)^75^. The computer program epestfind, as implemented in EMBOSS (http://emboss.bioinformatics.nl/cgi-bin/emboss/epestfind)^76^, originally written by Rogers and Rechstein, was used to predict putative PEST motifs. The 9aaTAD server was used to predict 9aaTAD motifs^77^.

Neanderthal and Denisovan genomes were searched at www.eva.mpg.de/denisova/index.html44. The Exome Variant Server was accessed at NHLBI GO Exome Sequencing Project (ESP), Seattle, WA (URL: http://evs.gs.washington.edu/EVS/).

### Homology modelling of LEUTX-DNA interaction

Homeodomain-DNA complexes were identified in the Nucleic Acid Database (NDB)^78^. The X-ray structures of relevant complexes were downloaded from the Protein Data Bank (PDB)^79^; the complexes were filtered based on the specific homeodomain binding motif TAATCC nucleotide sequence recognized by LEUTX. The sequences of the LEUTX homeodomain were searched against the PDB using blastp at NCBI^64^ in order to identify the best matching PDB entries. The engrailed homeodomain from *Drosophila melanogaster* at 1.9 Å resolution (PDB ID: 2HDD^13^; the quality of the initial model is shown in Additional File 1: Fig. S14) shares 40% sequence identity with LEUTX over the homeodomain and was chosen as the template protein for modelling *LEUTX* using the Homodge package in BODIL^80^ and MODELLER^81^. The structure-based sequence alignment and optimization of the matching of the query and template protein sequences were done using Vertaa and Malign in BODIL^80^. Four N-terminal residues not seen in the template structure were modelled in LEUTX using the 1.85 Å resolution structure of human DLX5 (PDB ID: 4RDU, chain D; Joint Center for Structural Genomics (JCSG), Partnership for Stem Cell Biology (STEMCELL)). The model structures were visualized and side-chain rotamers were checked for clashes and altered where necessary using the rotamer utility in BODIL^80^. Next, the models were energy minimized using the OPLS-2005 force field in the Maestro protein preparation wizard panel (Maestro version-10.3.015 Schrödinger suite). Structural features of the models were examined for standard acceptable values (e.g., for acceptable torsion angles and atom-atom contacts) using the MolProbity web server^82^. The model was then examined in detail, mutations of specific residues were introduced, and figures were prepared using PyMOL (The PyMOL Molecular Graphics System, Version 1.6 Schrödinger, LLC.) and Inkscape (www.inkscape.org).

## Electronic supplementary material

Supplementary Information

## Data Availability

Sequence data are provided in the Supplementary Material, 3D models are available upon request.
