# Supplementary material for: Phylogenetic and mutational analyses of human LEUTX, a homeobox gene implicated in embryogenesis
Source: Sci Rep. 2018 Nov 27;8:17421. doi: 10.1038/s41598-018-35547-5 (PMC6258689; doi:10.1038/s41598-018-35547-5)
Supplement: Supplementary file 1 — Supplementary Information [file 41598_2018_35547_MOESM1_ESM.pdf]

Supplementary Information

Phylogenetic and mutational analyses of human LEUTX, a homeobox gene implicated in embryogenesis

Shintaro Katayama[1], Vipin Ranga[2], Eeva-Mari Jouhilahti[1,6], Tomi T. Airenne[2], Mark S. Johnson[2], Krishanu Mukherjee[1,7], Thomas R. Bürglin[3¶]*, Juha Kere[1,4,5¶]*

[1] Department of Biosciences and Nutrition, Karolinska Institutet, Huddinge, Sweden

[2] Structural Bioinformatics Laboratory, Biochemistry, Faculty of Science and Engineering, Åbo Akademi University, Turku, Finland

[3] Department of Biomedicine, University of Basel, Basel, Switzerland

[4] Folkhälsan Institute of Genetics and Molecular Neurology Research Program, University of Helsinki, Helsinki, Finland

[5] School of Basic & Medical Biosciences, King's College London, London, England

[6] present address: Research Programs Unit, Molecular Neurology and Biomedicum Stem Cell Centre, Faculty of Medicine, University of Helsinki, Helsinki, Finland

[7] present address: The Whitney Laboratory for Marine Bioscience, University of Florida, St. Augustine, USA

[¶] These authors contributed equally to this work.

[*] Corresponding authors:

E-mail: thomas.buerglin@unibas.ch and juha.kere@ki.se

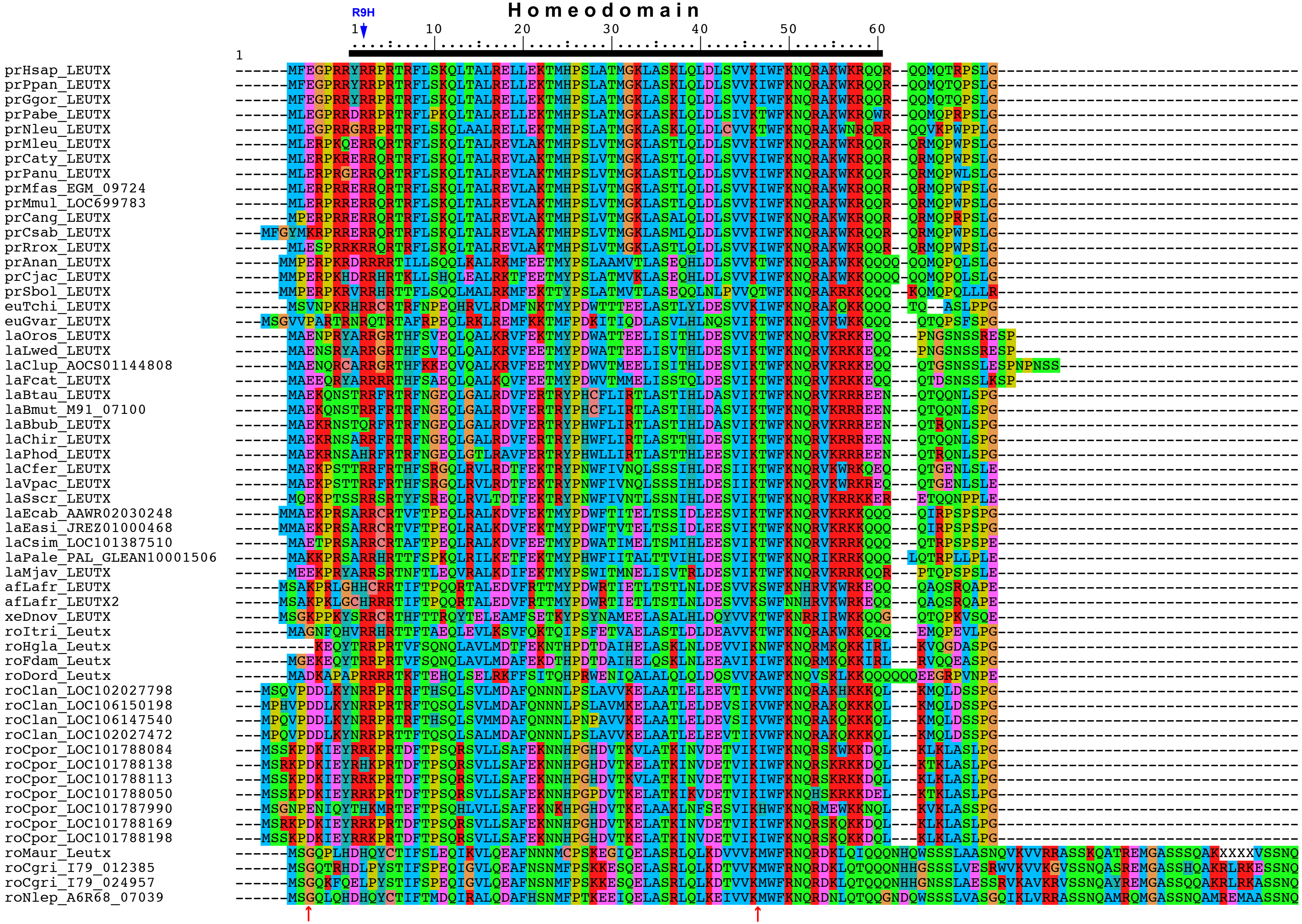
Homeodomain
R9H
1
10
20
30
40
50
60
1
prHsap_LEUTX
prPpan_LEUTX
prGgor_LEUTX
prPabe_LEUTX
prNleu_LEUTX
prMleu_LEUTX
prCaty_LEUTX
prPanu_LEUTX
prMfas_EGM_09724
prMmul_LOC699783
prCang_LEUTX
prCsab_LEUTX
prRrox_LEUTX
prAnan_LEUTX
prCjac_LEUTX
prSbol_LEUTX
euTchi_LEUTX
euGvar_LEUTX
laOros_LEUTX
laLwed_LEUTX
laClup_AOCS01144808
laFcat_LEUTX
laBtau_LEUTX
laBmut_M91_07100
laBbub_LEUTX
laChir_LEUTX
laPhod_LEUTX
laCfer_LEUTX
laVpac_LEUTX
laSscr_LEUTX
laEcab_AAWR02030248
laEasi_JREZ01000468
laCsim_LOC101387510
laPale_PAL_GLEAN10001506
laMjav_LEUTX
afLafr_LEUTX
afLafr_LEUTX2
xeDnov_LEUTX
roItri_Leutx
roHgla_Leutx
roFdam_Leutx
roDord_Leutx
roClan_LOC102027798
roClan_LOC106150198
roClan_LOC106147540
roClan_LOC102027472
roCpor_LOC101788084
roCpor_LOC101788138
roCpor_LOC101788113
roCpor_LOC101788050
roCpor_LOC101787990
roCpor_LOC101788169
roCpor_LOC101788198
roMaur_Leutx
roCgri_I79_012385
roCgri_I79_024957
roNlep_A6R68_07039

```
                              841
prHsap_LEUTX                  -----------------------------------------------------------------------------------------------------------------PANQTTS
prPpan_LEUTX                  -----------------------------------------------------------------------------------------------------------------PANQTIS
prGgor_LEUTX                  -----------------------------------------------------------------------------------------------------------------PANQTIS
prPabe_LEUTX                  -----------------------------------------------------------------------------------------------------------------PANQTIS
prNleu_LEUTX                  -----------------------------------------------------------------------------------------------------------------PANQTIS
prMleu_LEUTX                  -----------------------------------------------------------------------------------------------------------------PSNQTLS
prCaty_LEUTX                  -----------------------------------------------------------------------------------------------------------------PSNQTLS
prPanu_LEUTX                  -----------------------------------------------------------------------------------------------------------------PSNQTLS
prMfas_EGM_09724              -----------------------------------------------------------------------------------------------------------------PSNQTLS
prMmul_LOC699783              -----------------------------------------------------------------------------------------------------------------PSNQTLS
prCang_LEUTX                  -----------------------------------------------------------------------------------------------------------------PSNQTLS
prCsab_LEUTX                  -----------------------------------------------------------------------------------------------------------------PSNQTLS
prRrox_LEUTX                  -----------------------------------------------------------------------------------------------------------------PSNQTLS
prAnan_LEUTX                  -----------------------------------------------------------------------------------------------------------------ASSQTIS
prCjac_LEUTX                  -----------------------------------------------------------------------------------------------------------------ASSRTIS
prSbol_LEUTX                  -----------------------------------------------------------------------------------------------------------------ASRQTIS
euTchi_LEUTX                  ----------------------------------------------------------------------------------------------------------------------AP
euGvar_LEUTX                  -----------------------------------------------------------------------------------------------------------------ISNQTFS
laOros_LEUTX                  -------------------------------------------------------------------------------------------------------------------NPTIP
laLwed_LEUTX                  -------------------------------------------------------------------------------------------------------------------NPTVP
laClup_AOCS01144808           ---------------------------------------------------------------------------------------------------------------LESSNPTIS
laFcat_LEUTX                  -------------------------------------------------------------------------------------------------------------------NQTIS
laBtau_LEUTX                  -----------------------------------------------------------------------------------------------------------------DPRRVVS
laBmut_M91_07100              -----------------------------------------------------------------------------------------------------------------DPRRVVS
laBbub_LEUTX                  -----------------------------------------------------------------------------------------------------------------DPRQVVS
laChir_LEUTX                  -----------------------------------------------------------------------------------------------------------------DPCQVVS
laPhod_LEUTX                  -----------------------------------------------------------------------------------------------------------------DPCQVVS
laCfer_LEUTX                  -----------------------------------------------------------------------------------------------------------------DLQQVSS
laVpac_LEUTX                  -----------------------------------------------------------------------------------------------------------------DPQQVSS
laSscr_LEUTX                  -----------------------------------------------------------------------------------------------------------------DTPQVSP
laEcab_AAWR02030248           -----------------------------------------------------------------------------------------------------------------GPNQTTS
laEasi_JREZ01000468           -----------------------------------------------------------------------------------------------------------------GPNQTTS
laCsim_LOC101387510           -----------------------------------------------------------------------------------------------------------------APNQTTS
laPale_PAL_GLEAN10001506      -----------------------------------------------------------------------------------------------------------------APNQTIS
laMjav_LEUTX                  -----------------------------------------------------------------------------------------------------------------APYKTAS
afLafr_LEUTX                  -----------------------------------------------------------------------------------------------------------------EPNHVAP
afLafr_LEUTX2                 -----------------------------------------------------------------------------------------------------------------EPNQVAP
xeDnov_LEUTX                  -----------------------------------------------------------------------------------------------------------------APTKTVS
roItri_Leutx                  -----------------------------------------------------------------------------------------------------------------PSTQNTA
roHgla_Leutx                  -----------------------------------------------------------------------------------------------------------------TSTQQVL
roFdam_Leutx                  -----------------------------------------------------------------------------------------------------------------TSTQHVL
roDord_Leutx                  -----------------------------------------------------------------------------------------------------------------APQDQAN
roClan_LOC102027798           -----------------------------------------------------------------------------------------------------------------TSMKHVL
roClan_LOC106150198           -----------------------------------------------------------------------------------------------------------------TSMQHVF
roClan_LOC106147540           -----------------------------------------------------------------------------------------------------------------TSTRHVF
roClan_LOC102027472           -----------------------------------------------------------------------------------------------------------------TSTQHVL
roCpor_LOC101788084           -----------------------------------------------------------------------------------------------------------------TSMQTSS
roCpor_LOC101788138           -----------------------------------------------------------------------------------------------------------------TSTQTSS
roCpor_LOC101788113           -----------------------------------------------------------------------------------------------------------------TSTQTSS
roCpor_LOC101788050           -----------------------------------------------------------------------------------------------------------------TSTQTSS
roCpor_LOC101787990           -----------------------------------------------------------------------------------------------------------------TSTQPSL
roCpor_LOC101788169           -----------------------------------------------------------------------------------------------------------------TSTQTSS
roCpor_LOC101788198           -----------------------------------------------------------------------------------------------------------------TSTQTSS
roMaur_Leutx                  -----------------------------------------------------------------------------------------------------ASSNQAKKVRRASSKQVKS
roCgri_I79_012385             RSLSSTQDKRVKKASSNLAKIDRDAFSSPAPREIGAPSNQAPRKKGASSNQGKTVRKQSSNDAKGVKRVSSKQAMKE-----------RDASSNQTPREMGAPSNQDKTVRGVASMQEKS
roCgri_I79_024957             GALSNQA-PREMDVSSNQALREMGSSSNKASSEMVAQSNQEPREKGALCNEASRDMCTLSNQALRTMDASSNQALREMDASWIQELRDMSVLLKDASKEMDASINQAETVRGAPSKQMIS
roNlep_A6R68_07039            RRASSNQRTIEMGASSNQETLEMDASSNQGTTEMGASSNQETLEMDASPNQGTIEMGASSNQETIEMDALSNQGTIEMDASSNQETLEMDASSNQATRDMGASSNQAKPIRGAVCKQVNF
```

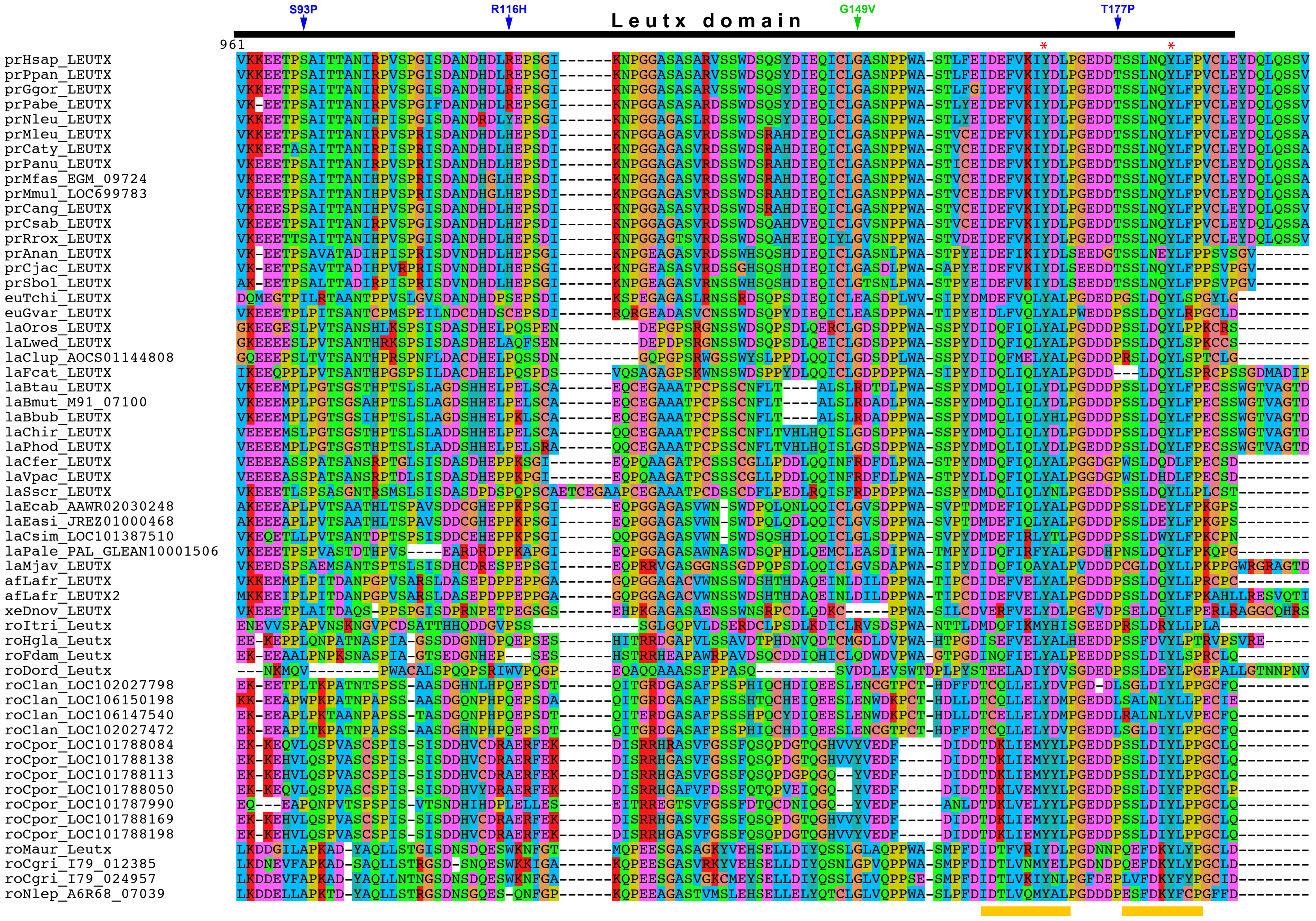
S93P
R116H
G149V
T177P
Leutx domain
961
prHsap_LEUTX
prPpan_LEUTX
prGgor_LEUTX
prPabe_LEUTX
prNleu_LEUTX
prMleu_LEUTX
prCaty_LEUTX
prPanu_LEUTX
prMfas_EGM_09724
prMmul_LOC699783
prCang_LEUTX
prCsab_LEUTX
prRrox_LEUTX
prAnan_LEUTX
prCjac_LEUTX
prSbol_LEUTX
euTchi_LEUTX
euGvar_LEUTX
laOros_LEUTX
laLwed_LEUTX
laClup_AOCS01144808
laFcat_LEUTX
laBtau_LEUTX
laBmut_M91_07100
laBbub_LEUTX
laChir_LEUTX
laPhod_LEUTX
laCfer_LEUTX
laVpac_LEUTX
laSscr_LEUTX
laEcab_AAWR02030248
laEasi_JREZ01000468
laCsim_LOC101387510
laPale_PAL_GLEAN10001506
laMjav_LEUTX
afLafr_LEUTX
afLafr_LEUTX2
xeDnov_LEUTX
roItri_Leutx
roHgla_Leutx
roFdam_Leutx
roDord_Leutx
roClan_LOC102027798
roClan_LOC106150198
roClan_LOC106147540
roClan_LOC102027472
roCpor_LOC101788084
roCpor_LOC101788138
roCpor_LOC101788113
roCpor_LOC101788050
roCpor_LOC101787990
roCpor_LOC101788169
roCpor_LOC101788198
roMaur_Leutx
roCgri_I79_012385
roCgri_I79_024957
roNlep_A6R68_07039

```
                              1081
prHsap_LEUTX                  ----------------------------
prPpan_LEUTX                  ----------------------------
prGgor_LEUTX                  ----------------------------
prPabe_LEUTX                  ----------------------------
prNleu_LEUTX                  ----------------------------
prMleu_LEUTX                  ----------------------------
prCaty_LEUTX                  ----------------------------
prPanu_LEUTX                  ----------------------------
prMfas_EGM_09724              ----------------------------
prMmul_LOC699783              ----------------------------
prCang_LEUTX                  ----------------------------
prCsab_LEUTX                  ----------------------------
prRrox_LEUTX                  ----------------------------
prAnan_LEUTX                  ----------------------------
prCjac_LEUTX                  ----------------------------
prSbol_LEUTX                  ----------------------------
euTchi_LEUTX                  ----------------------------
euGvar_LEUTX                  ----------------------------
laOros_LEUTX                  ----------------------------
laLwed_LEUTX                  ----------------------------
laClup_AOCS01144808           ----------------------------
laFcat_LEUTX                  TLIEKDL---------------------
laBtau_LEUTX                  HHREDEHSYNPEKVP-------------
laBmut_M91_07100              HHREDEHSYNPEKVP-------------
laBbub_LEUTX                  HHRDGEHSYNPEKVP-------------
laChir_LEUTX                  HHRDGEHSFNPEKVP-------------
laPhod_LEUTX                  HHRDGEHSFNPEKVP-------------
laCfer_LEUTX                  ----------------------------
laVpac_LEUTX                  ----------------------------
laSscr_LEUTX                  ----------------------------
laEcab_AAWR02030248           ----------------------------
laEasi_JREZ01000468           ----------------------------
laCsim_LOC101387510           ----------------------------
laPale_PAL_GLEAN10001506      ----------------------------
laMjav_LEUTX                  LQCDG-----------------------
afLafr_LEUTX                  ----------------------------
afLafr_LEUTX2                 NSS-------------------------
xeDnov_LEUTX                  SGGLLTSTAPENDL--------------
roItri_Leutx                  ----------------------------
roHgla_Leutx                  ----------------------------
roFdam_Leutx                  ----------------------------
roDord_Leutx                  SPDSNISQAWERWTLHTHCWPSVPTKSK
roClan_LOC102027798           ----------------------------
roClan_LOC106150198           ----------------------------
roClan_LOC106147540           ----------------------------
roClan_LOC102027472           ----------------------------
roCpor_LOC101788084           ----------------------------
roCpor_LOC101788138           ----------------------------
roCpor_LOC101788113           ----------------------------
roCpor_LOC101788050           ----------------------------
roCpor_LOC101787990           ----------------------------
roCpor_LOC101788169           ----------------------------
roCpor_LOC101788198           ----------------------------
roMaur_Leutx                  ----------------------------
roCgri_I79_012385             ----------------------------
roCgri_I79_024957             ----------------------------
roNlep_A6R68_07039            ----------------------------
```

Fig. S1. Multiple sequence alignment (MSA) of Leutx proteins. Homeodomain (including numbering) and Leutx domain are marked. Putative pseudogenes, as well as ORFs not containing a homeodomain have been omitted. The extensive linker region between the homeodomain and the Leutx domain, which is present in only a few sequences, has also been removed here. X mark gaps/unknown residues. For sequence identifiers, see supplementary table S1. In the case of prCsab_LEUTX the syntenic position of the first exon contained a point mutation at the putative start Met location with no upstream Met; hence we left the first exon as originally predicted in the Genbank record. In euGvar_LEUTX the first splice acceptor site is moved by three nucleotides towards the homeobox, and the syntenic first exon encodes a somewhat longer ORF. For roHgla_Leutx no syntenic first exon was found on the short contig. The canonical splices sites are indicated underneath the MSA with red arrows. Four positions with variations in human LEUTX are marked with blue arrows above the MSA. One position changed in Denisovan is marked with a green arrow. The two red asteriks mark highly conserved tyrosine residues. The two orange bars underneath the C-terminal region of the Leutx domain mark the most likely position of 9aaTAD motifs.

```
                             1
roItri_Leutx                 ------MAGNFQHVRRHRTTFTAEQLEVLKSVFQKTQIPSFETVAELASTLDLDEAVVKTWFKNQRAKWKKQQQEMQPEVLPGP------------------------------------
roHgla_Leutx                 ---------KEQYTRRPRTVFSQNQLAVLMDTFEKNTHPDTDAIHELASKLNLDEVVIKNWFKNQRMKQKKIRLKVQGDASPGT------------------------------------
roFdam_Leutx                 ------MGEKEQYTRRPRTVFSQNQLAVLMDAFEKDTHPDTDAIHELQSKLNLEEAVIKIWFKNQRMKQKKIRLRVQQEASPGT------------------------------------
roCcan_Leutx                 ------MSERPHHARRQRTRFSEGQLRVLRDVFNETKRPDWDTVQALAARLQLDVAVIKTWFKNNLVRWKKQQQEKEPNPKPGG------------------------------------
roDord_Leutx                 ------MADKAPAPRRRRTKFTEHQLSELRKFFSITQHPRWENIQALALQLQLDQSVVKAWFKNQVSKLKKQQQQQQEEGRPVN------------------------------------
roDord_LOC105990859p         ------MAEQLRAHRKSRTRFTQHQLQELRAAFKHTQHPKWDEVQELASRLQLDEIEVKVWFKNQKAKLKKQQQA---GKPPVC------------------------------------
roDord_LOC105990857p         ------MAEQLRAHRKSRTRFTQHQLQELRAAFKHTQHPKWDEVQELASRLQLDEIEVKVWFKNQKAKLKKQQQA---GKPPVC------------------------------------
roClan_LOC102027798          ---MSQVPDDLKYNRRPRTRFTHSQLSVLMDAFQNNNLPSLAVVKELAATLELEEVTIKVWFKNQRAKHKKKQLKMQLDSSPGT------------------------------------
roClan_LOC106150198          ---MPHVPDDLKYNRRPRTRFTQSQLSVLMDAFQNNNLPSLAVMKELAATLELDEVSIKVWFKNQRAKQKKKQLKMQLDSSPGT------------------------------------
roClan_LOC106147540          ---MPQVPDDLKYNRRPRTRFTHSQLSVMMDAFQNNNLPNPAVVKELAATLELDEVSIKVWFKNQRAKQKKKQLKMQLDSSPGT------------------------------------
roClan_LOC102027472          ---MPQVPDDLKYNRRPRTTFTQSQLSALMDAFQNNNLPSLAVVKELAATLELEEVTIKVWFKNQRAKQKKKQLKMQLDSSPGT------------------------------------
roCpor_LOC101788084          ---MSSKPDKIEYRRKPRTDFTPSQRSVLLSAFEKNNHPGHDVTKVLATKINVDETVIKIWFKNQRSKWKKDQLKLKLASLPGT------------------------------------
roCpor_LOC101788138          ---MSRKPDKIEYRHKPRTDFTPSQRSVLLSAFEKNNHPGHDVTKELATKINVDETVIKIWFKNQRSKRKKDQLKTKLASLPGT------------------------------------
roCpor_LOC101788113          ---MSSKPDKIEYRRKPRTDFTPSQRSVLLSAFEKNNHPGHDVTKVLATKINVDETVIKIWFKNQRSKRKKDQLKLKLASLPGT------------------------------------
roCpor_LOC101788050          ---MSSKPDKIEYRRKPRTDFTPSQRSVLLSAFEKNNHPGPDVTKELATKIKVDETVIKIWFKNQHSKRKKDELKTKLASLPGT------------------------------------
roCpor_LOC101787990          ---MSGNPENIQYTHKMRTEFTPSQHLVLLSAFEKNKHPGHDVTKELAAKLNFSESVIKHWFKNQRMEWKKNQLKVKLASSPGT------------------------------------
roCpor_LOC101788169          ---MSRKPDKIEYRRKPRTDFTPSQRSVLLSAFEKNNHPGHDVTKELATKINVDETVIKIWFKNQRSKQKKDQLKLKLASLPGT------------------------------------
roCpor_LOC101788198          ---MSSKPDKIEYRRKPRTDFTPSQRSVLLSAFEKNNHPGHDVTKELATKINVDETVIKIWFKNQRSKQKKDQLKLKLASLPGT------------------------------------
roCpor_LOC101788018p         ---------KIEYRRKPRTDFTPSQRSVLLSAFENNNHPGHDVTKELATKINVDETVIKIWFKKQCSKRKKDQLKMKLASLPGT------------------------------------
roMaur_Leutx                 ------MSGQPLHDHQYCTIFSLEQIKVLQEAFNNNMCPSKEGIQELASRLQLKDVVVKMWFRNQRDKLQIQQQNHQWSSSLAASNQVKVVRRASSKQATREMGASSSQAKXXXXVSSNQ
roCgri_I79_012385            ------MSGQTRHDLPYSTIFSPEQIGVLQEAFNSNMFPSKKESQELASRLQLKDTVVKMWFRNQRDKLQTQQQNHHGSSSLVESRWVKVVKGVSSNQASREMGASSHQAKRLRKESSNQ
roCgri_I79_024957            ------MSGQKFQELPYSTIFSPEQIGVLQEAFNSNMFPSKKESQELASRLQLKDTVVKMWFRNQRDKLQTQQQNHHGNSSLAESSRVKAVKRVSSNQAYREMGASSQQAKRLRKASSNQ
roNlep_A6R68_07039           ------MSGQLQHDHQYCTIFTMDQIRALQDAFHSNMFPTKEEIQELASRLQLKEIVVKMWFKNQRDNLQTQQGNDQWSSSLVASGQIKLVRRASSNQAMRQMG----------------
lagOpri_AAYZ01197513         ---------SGNAARRPRTGFKPQQLGVLKAAFEKTPCPDLDTLESLAMELNLEMSVVKTWFKNKRGNLSRHQQKQKQEGLPKRLAPV-AW--DTSSHAMQEKSLSDSNLNPPKIVPFHL
lagOcun_LEUTX                MSSEQVAEAAPRCARRSRTAFTPDQLGMLKAVFETTPCPDWEKIQELAAKLCLDECVIKTWFKNQRAQQRKLQRKGQPRQPGES------------------------------------
lagOcun_LOC103348417         MSSEQVAEAVPRSSRRSRTAFTPDQLGMLQAVLETSRCPDWSRVQELAPELHLDVYVIKTWFKNQRAQQRKLQPKGQPRQPSES------------------------------------
lagOcun_LOC103348415         ----------PRSSRRSRTAFTPDQLGMLKAVLETSRCPDWSQKEELSSELHLDAYGIKTWFKNQRAKQRKLQPTGQPRQPSES------------------------------------
lagOcun_LOC103347612         -----------------------------------------------------------TWFKNQRAKQRKLQRKGQPRQPSES------------------------------------
lagOcun_LOC103348423         ----------PRCARRSRMAFTPDQLGMLKAVFETTPCPDWEKIQELAAKLCLDECVIKTWFKNQRAQQRKLQPKGQPRQPGES------------------------------------
lagOcun_AAGW02026615         ------------------------------------------------------------------------------------------------------------------------
roPman_AYHN01138932          ------------------------------------------------------------------------------------------------------------------------
roPman_Leutx                 ------------------------------------------------------------------------------------------------------------------------
roMmus_Leutxp                ---------SHASDGAWFNQATPVN----GASMNQ-AMPVNR------ALMNQATPVSRAWFNQTTP---------------VSRAWFN--------QATPVIG----------------
roRnor_Leutxp                ------------------------------------------------------------------------------------------------------------------------
prHsap_LEUTX                 ------MFEGPRRYRRPRTRFLSKQLTALRELLEKTMHPSLATMGKLASKLQLDLSVVKIWFKNQRAKWKRQQRQQMQTRPSLG------------------------------------
```

```
                         121
roItri_Leutx             ------------------------------------------------------------------------------------------------------------------------
roHgla_Leutx             ------------------------------------------------------------------------------------------------------------------------
roFdam_Leutx             ------------------------------------------------------------------------------------------------------------------------
roCcan_Leutx             ------------------------------------------------------------------------------------------------------------------------
roDord_Leutx             ------------------------------------------------------------------------------------------------------------------------
roDord_LOC105990859p     ------------------------------------------------------------------------------------------------------------------------
roDord_LOC105990857p     ------------------------------------------------------------------------------------------------------------------------
roClan_LOC102027798      ------------------------------------------------------------------------------------------------------------------------
roClan_LOC106150198      ------------------------------------------------------------------------------------------------------------------------
roClan_LOC106147540      ------------------------------------------------------------------------------------------------------------------------
roClan_LOC102027472      ------------------------------------------------------------------------------------------------------------------------
roCpor_LOC101788084      ------------------------------------------------------------------------------------------------------------------------
roCpor_LOC101788138      ------------------------------------------------------------------------------------------------------------------------
roCpor_LOC101788113      ------------------------------------------------------------------------------------------------------------------------
roCpor_LOC101788050      ------------------------------------------------------------------------------------------------------------------------
roCpor_LOC101787990      ------------------------------------------------------------------------------------------------------------------------
roCpor_LOC101788169      ------------------------------------------------------------------------------------------------------------------------
roCpor_LOC101788198      ------------------------------------------------------------------------------------------------------------------------
roCpor_LOC101788018p     ------------------------------------------------------------------------------------------------------------------------
roMaur_Leutx             A-----------------------------------------------------------------------------------------------------------------------
roCgri_I79_012385        GSREMDASSNQGQRRMGPSSNEGPDGMIALCNQSPREMAASSNQPPREMDESSNQAKRVRKASSNHIPREKGPPSNLALREMDASSNQPPRQMGASSNQPPREMDASSNQPPREMDASSN
roCgri_I79_024957        VPRNMKTLSNQSSRQMHISANQLPRQMDALFKQASR--------------------------------------------EMGASSNQGPRVMGPSFNQAPSEMCPSSNQVPRDMDAVF-
roNlep_A6R68_07039       ------------------------------------------------------------------------------------------------------------------------
lagOpri_AAYZ01197513     QPLPPQ------------------------------------------------------------------------------------------------------------------
lagOcun_LEUTX            ------------------------------------------------------------------------------------------------------------------------
lagOcun_LOC103348417     ------------------------------------------------------------------------------------------------------------------------
lagOcun_LOC103348415     ------------------------------------------------------------------------------------------------------------------------
lagOcun_LOC103347612     ------------------------------------------------------------------------------------------------------------------------
lagOcun_LOC103348423     ------------------------------------------------------------------------------------------------------------------------
lagOcun_AAGW02026615     ------------------------------------------------------------------------------------------------------------------------
roPman_AYHN01138932      ------------------------------------------------------------------------------------------------------------------------
roPman_Leutx             ------------------------------------------------------------------------------------------------------------------------
roMmus_Leutxp            -----------------------------------------------------------------------------------TSSNQSTPVHGASMNQATPVNG---------------
roRnor_Leutxp            ------------------------------------------------------------------------------------------------------------------------
prHsap_LEUTX             ------------------------------------------------------------------------------------------------------------------------
```

```
                          241
roItri_Leutx              ------------------------------------------------------------------------------------------------------------------------
roHgla_Leutx              ------------------------------------------------------------------------------------------------------------------------
roFdam_Leutx              ------------------------------------------------------------------------------------------------------------------------
roCcan_Leutx              ------------------------------------------------------------------------------------------------------------------------
roDord_Leutx              ------------------------------------------------------------------------------------------------------------------------
roDord_LOC105990859p      ------------------------------------------------------------------------------------------------------------------------
roDord_LOC105990857p      ------------------------------------------------------------------------------------------------------------------------
roClan_LOC102027798       ------------------------------------------------------------------------------------------------------------------------
roClan_LOC106150198       ------------------------------------------------------------------------------------------------------------------------
roClan_LOC106147540       ------------------------------------------------------------------------------------------------------------------------
roClan_LOC102027472       ------------------------------------------------------------------------------------------------------------------------
roCpor_LOC101788084       ------------------------------------------------------------------------------------------------------------------------
roCpor_LOC101788138       ------------------------------------------------------------------------------------------------------------------------
roCpor_LOC101788113       ------------------------------------------------------------------------------------------------------------------------
roCpor_LOC101788050       ------------------------------------------------------------------------------------------------------------------------
roCpor_LOC101787990       ------------------------------------------------------------------------------------------------------------------------
roCpor_LOC101788169       ------------------------------------------------------------------------------------------------------------------------
roCpor_LOC101788198       ------------------------------------------------------------------------------------------------------------------------
roCpor_LOC101788018p      ------------------------------------------------------------------------------------------------------------------------
roMaur_Leutx              ------------------------------------------------------------------------------------------------------------------------
roCgri_I79_012385         QAKRVRKASSNQAPREMDAPSNQNKRVKKASSNQRPREMGPSSNQPPRQTGASSNQALREMGPSSNQPPREMDASSIQAKRVRKASSNHMPREKGPPTNQALTEMGVSSNQLPREMIALF
roCgri_I79_024957         --------------------------------NQEPREMGASSNQ--------------------------------------------GPRVMGPSFNQAPSEMCPSSNQVPRDMDAVF
roNlep_A6R68_07039        ----------------------------------------------------------------------------------------------------------ASSNQAMREMAASS
lagOpri_AAYZ01197513      ------------------------------------------------------------------------------------------------------------------------
lagOcun_LEUTX             ------------------------------------------------------------------------------------------------------------------------
lagOcun_LOC103348417      ------------------------------------------------------------------------------------------------------------------------
lagOcun_LOC103348415      ------------------------------------------------------------------------------------------------------------------------
lagOcun_LOC103347612      ------------------------------------------------------------------------------------------------------------------------
lagOcun_LOC103348423      ------------------------------------------------------------------------------------------------------------------------
lagOcun_AAGW02026615      ------------------------------------------------------------------------------------------------------------------------
roPman_AYHN01138932       ------------------------------------------------------------------------------------------------------------------------
roPman_Leutx              ------------------------------------------------------------------------------------------------------------------------
roMmus_Leutxp             ------------------ALMNQTTPVSRAWFNQATPVIGTSSNQSTPVHGASMNQATPVNRASMNQAMPVNGALMNQATPVNGALMNQATPVNGALMNQAMPGIGTSSNQAMPGSEAWF
roRnor_Leutxp             ------------------------------------------------------------------------------------------------------------------------
prHsap_LEUTX              ------------------------------------------------------------------------------------------------------------------------
```

```
                         361
roItri_Leutx             ------------------------------------------------------------------------------------------------------------------------
roHgla_Leutx             ------------------------------------------------------------------------------------------------------------------------
roFdam_Leutx             ------------------------------------------------------------------------------------------------------------------------
roCcan_Leutx             ------------------------------------------------------------------------------------------------------------------------
roDord_Leutx             ------------------------------------------------------------------------------------------------------------------------
roDord_LOC105990859p     ------------------------------------------------------------------------------------------------------------------------
roDord_LOC105990857p     ------------------------------------------------------------------------------------------------------------------------
roClan_LOC102027798      ------------------------------------------------------------------------------------------------------------------------
roClan_LOC106150198      ------------------------------------------------------------------------------------------------------------------------
roClan_LOC106147540      ------------------------------------------------------------------------------------------------------------------------
roClan_LOC102027472      ------------------------------------------------------------------------------------------------------------------------
roCpor_LOC101788084      ------------------------------------------------------------------------------------------------------------------------
roCpor_LOC101788138      ------------------------------------------------------------------------------------------------------------------------
roCpor_LOC101788113      ------------------------------------------------------------------------------------------------------------------------
roCpor_LOC101788050      ------------------------------------------------------------------------------------------------------------------------
roCpor_LOC101787990      ------------------------------------------------------------------------------------------------------------------------
roCpor_LOC101788169      ------------------------------------------------------------------------------------------------------------------------
roCpor_LOC101788198      ------------------------------------------------------------------------------------------------------------------------
roCpor_LOC101788018p     ------------------------------------------------------------------------------------------------------------------------
roMaur_Leutx             ----------------------------------------------------------PREMGASSSQS---------------------------------------------------
roCgri_I79_012385        NQAPREMAASSKQGTREMDALFNHALREIGASSNHAKRQRNAPSNQPPRQMGASSNQPPREMDASSNQAKRVRKASSNHAPREKIPPSNQAPREIAASSNQGPREMDVLFNQALREMGAS
roCgri_I79_024957        NQEPREMGSSSNQPQRKIDAFFNQASKGMGASSNQGP-----------REIYALSDKASREMGTSSNQA---------------------------------------------------
roNlep_A6R68_07039       NQAMREMGASSNQVMVGLASSYSQATIEMAASSNQAKRVRRASSNKAKRVRGASSNKAKRVRG----------------------------------------------
lagOpri_AAYZ01197513     ------------------------------------------------------------------------------------------------------------------------
lagOcun_LEUTX            ------------------------------------------------------------------------------------------------------------------------
lagOcun_LOC103348417     ------------------------------------------------------------------------------------------------------------------------
lagOcun_LOC103348415     ------------------------------------------------------------------------------------------------------------------------
lagOcun_LOC103347612     ------------------------------------------------------------------------------------------------------------------------
lagOcun_LOC103348423     ------------------------------------------------------------------------------------------------------------------------
lagOcun_AAGW02026615     -----------------MEGTQLLEPSLLTPWVSINKKVKIEADQGLEPKHSHMGCCTKHLPKRFYLKYIHCSTTLFTNNVSSTSVQTATLYSLIRTYHN--------------------
roPman_AYHN01138932      ------------------------------------------------------------------------------------------------------------------------
roPman_Leutx             ------------------------------------------------------------------------------------------------------------------------
roMmus_Leutxp            NQAMPVREPLSNQATSVRRTLFNQAMPEIGTSSNQAMPVSEASINQAMPVSEASINQAMPVSGASINQAMPVSEASSNQAM----------------------PVSGALINQAMPVSKAS
roRnor_Leutxp            ------------------------------------------------------------------------------------------------------------------------
prHsap_LEUTX             ------------------------------------------------------------------------------------------------------------------------
```

```
                                481
roItri_Leutx                    ------------------------------------------------------------------------------------------------------------------------
roHgla_Leutx                    ------------------------------------------------------------------------------------------------------------------------
roFdam_Leutx                    ------------------------------------------------------------------------------------------------------------------------
roCcan_Leutx                    ------------------------------------------------------------------------------------------------------------------------
roDord_Leutx                    ------------------------------------------------------------------------------------------------------------------------
roDord_LOC105990859p            ------------------------------------------------------------------------------------------------------------------------
roDord_LOC105990857p            ------------------------------------------------------------------------------------------------------------------------
roClan_LOC102027798             ------------------------------------------------------------------------------------------------------------------------
roClan_LOC106150198             ------------------------------------------------------------------------------------------------------------------------
roClan_LOC106147540             ------------------------------------------------------------------------------------------------------------------------
roClan_LOC102027472             ------------------------------------------------------------------------------------------------------------------------
roCpor_LOC101788084             ------------------------------------------------------------------------------------------------------------------------
roCpor_LOC101788138             ------------------------------------------------------------------------------------------------------------------------
roCpor_LOC101788113             ------------------------------------------------------------------------------------------------------------------------
roCpor_LOC101788050             ------------------------------------------------------------------------------------------------------------------------
roCpor_LOC101787990             ------------------------------------------------------------------------------------------------------------------------
roCpor_LOC101788169             ------------------------------------------------------------------------------------------------------------------------
roCpor_LOC101788198             ------------------------------------------------------------------------------------------------------------------------
roCpor_LOC101788018p            ------------------------------------------------------------------------------------------------------------------------
roMaur_Leutx                    -------------------------------------------------------------------------------------------KRVRK------------------------
roCgri_I79_012385               SNHAKRLRNETPSQPLRNMDALSNQPPRQMGASSNQPPREMDASSXXXREMDASSNQKPRVIATSSNQAKSEMGALSKQASREMWASPNQAKTVRKVSSNQAPREMGPSFNKAPREIRPK
roCgri_I79_024957               -------------------------------------------------------------------------------------------KRVRKASSNQAHREMGPSSN---------
roNlep_A6R68_07039              ------------------------------------------------------------------------------------------------------------------------
lagOpri_AAYZ01197513            ------------------------------------------------------------------------------------------------------------------------
lagOcun_LEUTX                   ------------------------------------------------------------------------------------------------------------------------
lagOcun_LOC103348417            ------------------------------------------------------------------------------------------------------------------------
lagOcun_LOC103348415            ------------------------------------------------------------------------------------------------------------------------
lagOcun_LOC103347612            ------------------------------------------------------------------------------------------------------------------------
lagOcun_LOC103348423            ------------------------------------------------------------------------------------------------------------------------
lagOcun_AAGW02026615            ------------------------------------------------------------------------------------------------------------------------
roPman_AYHN01138932             ------------------------------------------------------------------------------------------------------------------------
roPman_Leutx                    ------------------------------------------------------------------------------------------------------------------------
roMmus_Leutxp                   I-----------SQAMPVSGALINQA--------------------------------MPVSKASISQAMPVSGILINQAMPVSQASVNQVKLKRRASSNQAMPVSKALINQAMPVSEAS
roRnor_Leutxp                   ------------------------------------------------------------------------------------------------------------------------
prHsap_LEUTX                    ------------------------------------------------------------------------------------------------------------------------
```

```
                         601
roItri_Leutx           ------------------------------------------------------------------------------------------------------------------------
roHgla_Leutx           ------------------------------------------------------------------------------------------------------------------------
roFdam_Leutx           ------------------------------------------------------------------------------------------------------------------------
roCcan_Leutx           ------------------------------------------------------------------------------------------------------------------------
roDord_Leutx           ------------------------------------------------------------------------------------------------------------------------
roDord_LOC105990859p   ------------------------------------------------------------------------------------------------------------------------
roDord_LOC105990857p   ------------------------------------------------------------------------------------------------------------------------
roClan_LOC102027798    ------------------------------------------------------------------------------------------------------------------------
roClan_LOC106150198    ------------------------------------------------------------------------------------------------------------------------
roClan_LOC106147540    ------------------------------------------------------------------------------------------------------------------------
roClan_LOC102027472    ------------------------------------------------------------------------------------------------------------------------
roCpor_LOC101788084    ------------------------------------------------------------------------------------------------------------------------
roCpor_LOC101788138    ------------------------------------------------------------------------------------------------------------------------
roCpor_LOC101788113    ------------------------------------------------------------------------------------------------------------------------
roCpor_LOC101788050    ------------------------------------------------------------------------------------------------------------------------
roCpor_LOC101787990    ------------------------------------------------------------------------------------------------------------------------
roCpor_LOC101788169    ------------------------------------------------------------------------------------------------------------------------
roCpor_LOC101788198    ------------------------------------------------------------------------------------------------------------------------
roCpor_LOC101788018p   ------------------------------------------------------------------------------------------------------------------------
roMaur_Leutx           ------------------------------------------------------------------------------------------------------------------------
roCgri_I79_012385      VSSSQGPRELCPSSNQPLSKIDASSSQALKDMEALLDQALRERGASSNQPSREIDALSKLASKEM-----------DASTNPKPTEMGASSNQPAREMDALSKQASREMCASTNQPPREM
roCgri_I79_024957      ------------------------------------------------------------------------------------------------------------------------
roNlep_A6R68_07039     ----------------------------------------------------------------------------------------APSKQAMREMTASSNQRTIEMGASPNHAVREM
lagOpri_AAYZ01197513   ------------------------------------------------------------------------------------------------------------------------
lagOcun_LEUTX          ------------------------------------------------------------------------------------------------------------------------
lagOcun_LOC103348417   ------------------------------------------------------------------------------------------------------------------------
lagOcun_LOC103348415   ------------------------------------------------------------------------------------------------------------------------
lagOcun_LOC103347612   ------------------------------------------------------------------------------------------------------------------------
lagOcun_LOC103348423   ------------------------------------------------------------------------------------------------------------------------
lagOcun_AAGW02026615   ------------------------------------------------------------------------------------------------------------------------
roPman_AYHN01138932    --------------------------------------------------------------------------------------RRAASNQAKTVRSTSSSQAMREMCASSNQAMREM
roPman_Leutx           ------------------------------------------------------------------------------------------------------------------------
roMmus_Leutxp          INQAMPVSE--ASINQAMPVSGALINQAMPVSEALINQAMPVSEASSNQAMPVSGALINQAMPVSEASINQAMPVSEASSNQAMPVSGASINQAMPVSEASSNQAMPVSKASINQAMPVS
roRnor_Leutxp          --------------------------------------------------------------------------------------------------------GTSSREAWFNQAMPVS
prHsap_LEUTX           ------------------------------------------------------------------------------------------------------------------------
```

```
                         721
roItri_Leutx             ------------------------------------------------------------------------------------------------------------------------
roHgla_Leutx             ------------------------------------------------------------------------------------------------------------------------
roFdam_Leutx             ------------------------------------------------------------------------------------------------------------------------
roCcan_Leutx             ------------------------------------------------------------------------------------------------------------------------
roDord_Leutx             ------------------------------------------------------------------------------------------------------------------------
roDord_LOC105990859p     ------------------------------------------------------------------------------------------------------------------------
roDord_LOC105990857p     ------------------------------------------------------------------------------------------------------------------------
roClan_LOC102027798      ------------------------------------------------------------------------------------------------------------------------
roClan_LOC106150198      ------------------------------------------------------------------------------------------------------------------------
roClan_LOC106147540      ------------------------------------------------------------------------------------------------------------------------
roClan_LOC102027472      ------------------------------------------------------------------------------------------------------------------------
roCpor_LOC101788084      ------------------------------------------------------------------------------------------------------------------------
roCpor_LOC101788138      ------------------------------------------------------------------------------------------------------------------------
roCpor_LOC101788113      ------------------------------------------------------------------------------------------------------------------------
roCpor_LOC101788050      ------------------------------------------------------------------------------------------------------------------------
roCpor_LOC101787990      ------------------------------------------------------------------------------------------------------------------------
roCpor_LOC101788169      ------------------------------------------------------------------------------------------------------------------------
roCpor_LOC101788198      ------------------------------------------------------------------------------------------------------------------------
roCpor_LOC101788018p     ------------------------------------------------------------------------------------------------------------------------
roMaur_Leutx             ------------------------------------------------------------------------------------------------------------------------
roCgri_I79_012385        CASPNQPPSEIDASSSQALREMEALLDQALKEIGETSNQASREMDASSNKAPREVHASSNQASRETDASSYQVPREMAVSTSHALAELDELVNQLLREMNASSNQASREMD---------
roCgri_I79_024957        -----QAVSEMDALCRQALREMEALLNQTLREIGESSNQASREMDSSSNQAPREVDASSNPAPREMDALSNQVPREMGVSSSQALTELDALLNQLLREMNESSNQSSREMG---------
roNlep_A6R68_07039       GASSNQAITELAASSNLATLEMAVSSNQAMREMGASSNQVMVKLASSSSQATIEVAASSNKAKRVRGAPSKQAMREMTTSSNQRTIEMGASSNQAKKVRRALSNKAKRVRGAPSKQAMRE
lagOpri_AAYZ01197513     ----------------------------------------------------------------------PQSPSQAPTATSPQAPPASGHNSQHGSRPSHLDHLVLDPSAPWAFLPEHS
lagOcun_LEUTX            ------------------------------------------------------------------------------------------------------------------------
lagOcun_LOC103348417     ------------------------------------------------------------------------------------------------------------------------
lagOcun_LOC103348415     ------------------------------------------------------------------------------------------------------------------------
lagOcun_LOC103347612     ------------------------------------------------------------------------------------------------------------------------
lagOcun_LOC103348423     ------------------------------------------------------------------------------------------------------------------------
lagOcun_AAGW02026615     ------------------------------------------------------------------------------------------------------------------------
roPman_AYHN01138932      NVTSKQVMIKLPSSSSQAPMEMAVSSNQRTTEMGESSNQAKTVRRAASN----------------------QVNRVRR------------AASKQTIIEAASSNQATLEVA---------
roPman_Leutx             ------------------------------------------------------------------------------------------------------------------------
roMmus_Leutxp            GASINQVMPVSGALINQAMPVSGILINQAMPVSQASTNQVELKRRASSNQNPPVRKATFNQAKQR-----------RRASSNQAMPLSKALINQAMPVCEASINQAMPVSEASINQAMPV
roRnor_Leutxp            GALINEVMPARGALINQDTPMSGALINQATTV-----------------------------------------------------------------------------SGASINQAMAV
prHsap_LEUTX             ------------------------------------------------------------------------------------------------------------------------
```

```
                        841
roItri_Leutx            ------------------------------------------------------------------------------------------------------------------------
roHgla_Leutx            ------------------------------------------------------------------------------------------------------------------------
roFdam_Leutx            ------------------------------------------------------------------------------------------------------------------------
roCcan_Leutx            ------------------------------------------------------------------------------------------------------------------------
roDord_Leutx            ------------------------------------------------------------------------------------------------------------------------
roDord_LOC105990859p    ------------------------------------------------------------------------------------------------------------------------
roDord_LOC105990857p    ------------------------------------------------------------------------------------------------------------------------
roClan_LOC102027798     ------------------------------------------------------------------------------------------------------------------------
roClan_LOC106150198     ------------------------------------------------------------------------------------------------------------------------
roClan_LOC106147540     ------------------------------------------------------------------------------------------------------------------------
roClan_LOC102027472     ------------------------------------------------------------------------------------------------------------------------
roCpor_LOC101788084     ------------------------------------------------------------------------------------------------------------------------
roCpor_LOC101788138     ------------------------------------------------------------------------------------------------------------------------
roCpor_LOC101788113     ------------------------------------------------------------------------------------------------------------------------
roCpor_LOC101788050     ------------------------------------------------------------------------------------------------------------------------
roCpor_LOC101787990     ------------------------------------------------------------------------------------------------------------------------
roCpor_LOC101788169     ------------------------------------------------------------------------------------------------------------------------
roCpor_LOC101788198     ------------------------------------------------------------------------------------------------------------------------
roCpor_LOC101788018p    ------------------------------------------------------------------------------------------------------------------------
roMaur_Leutx            ------------------------------------------------------------------------------------------------------------------------
roCgri_I79_012385       --TSSNQA-----------KEVRKLSSKQAKQVRSLSSTQDKRVKKASSNLAKIDRDAFSSPAPREIGAPSNQAPRKKGASSNQGKTVRKQSSNDAKGVKRVSSKQ--AMKE--------
roCgri_I79_024957       --SLSNQD-----------SREMGPSE-TSREIGALSNQA-PREMDVSSNQALREMGSSSNKASSEMVAQSNQEPREKGALCNEASRDMCTLSNQALRTMDASSNQ--ALREMDASWIQE
roNlep_A6R68_07039      MTASSNQAIIELAASSNQVKKVRRASSNQRTIEMGASSNQETLEMDASSNQGTTEMGASSNQETLEMDASPNQGTIEMGASSNQETIEMDALSNQGTIEMDASSNQE-------------
lagOpri_AAYZ01197513    NMQSLLPQPQPHYQLPRYTPLDAPPASGNTSQQGSCPIQQELLCLDPLAPWASLPAHSHLQPLPEPPPQPHYQALTSTSQAPPASGHTSQHGSTPTHLEHLCLEAADSWASLSAHSHLQP
lagOcun_LEUTX           ------------------------------------------------------------------------------------------------------------------------
lagOcun_LOC103348417    ------------------------------------------------------------------------------------------------------------------------
lagOcun_LOC103348415    ------------------------------------------------------------------------------------------------------------------------
lagOcun_LOC103347612    ------------------------------------------------------------------------------------------------------------------------
lagOcun_LOC103348423    ------------------------------------------------------------------------------------------------------------------------
lagOcun_AAGW02026615    ------------------------------------------------------------------------------------------------------------------------
roPman_AYHN01138932     --ASSNQA-----------KRVRGASSKQSKRVGGASSNQETMEMAATSNQAKRVRGASSKHSKRVRGASSNQAKRVRGASSNQETMEMAATSNQGTMELAATSIQAKPVRGVPCKQVKS
roPman_Leutx            ---------------------------------------------------------------------------------SKQGTMEMA------------------------------
roMmus_Leutxp           SEASSNQAMPVSEASINQAMPVSGALINQAMPVSEALINQAMPVSEASSNQAMPVSGALINQAMPVSEASINQAMPVSGASSNQAMPVSGSSSNQAMPVSGASSNPAKQR----------
roRnor_Leutxp           SGALINQDTPRSRGLINQAMPVSGALINEAMPVRGVSVNQAMAMIRALFNQDTPMRGALI-QAMSVRGALINQAMPVNKALINEVMPRRKALPNHALPVREA------------------
prHsap_LEUTX            ------------------------------------------------------------------------------------------------------------------------
```

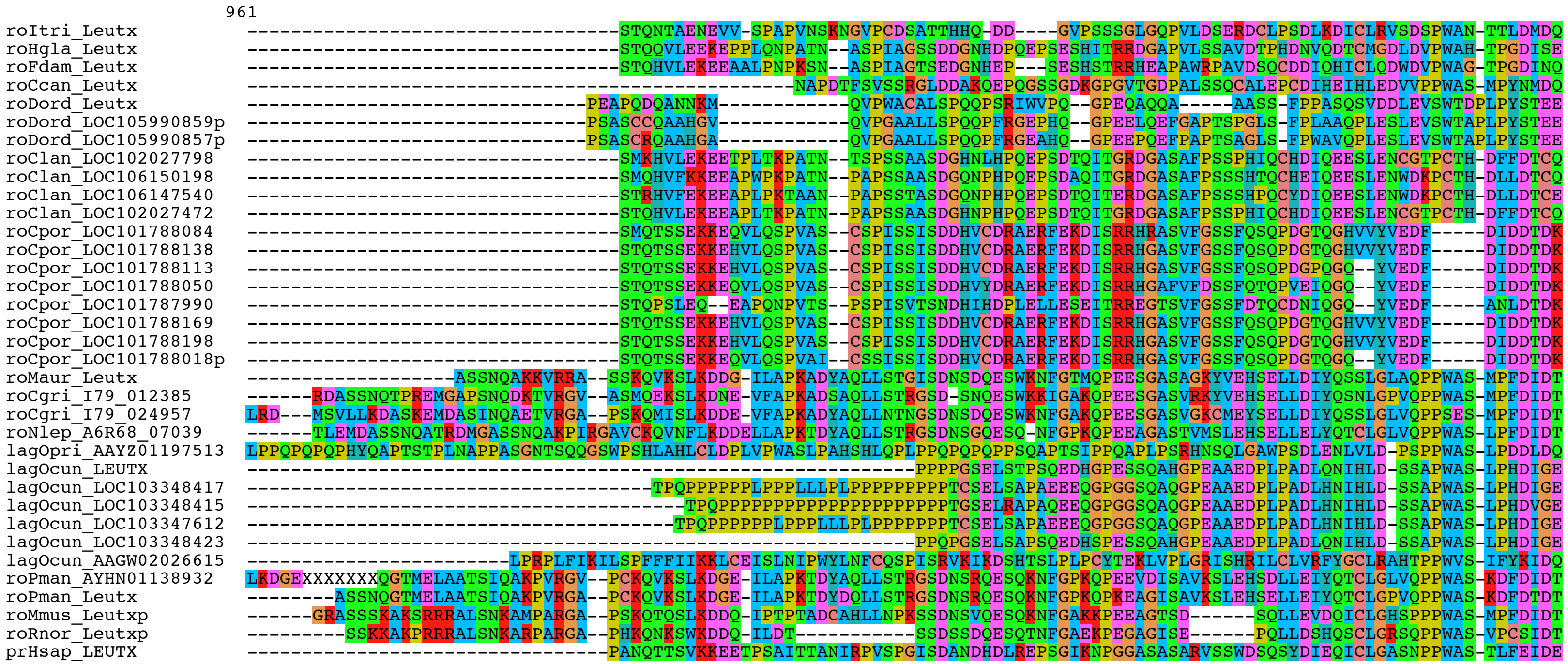
961
roItri_Leutx
roHgla_Leutx
roFdam_Leutx
roCcan_Leutx
roDord_Leutx
roDord_LOC105990859p
roDord_LOC105990857p
roClan_LOC102027798
roClan_LOC106150198
roClan_LOC106147540
roClan_LOC102027472
roCpor_LOC101788084
roCpor_LOC101788138
roCpor_LOC101788113
roCpor_LOC101788050
roCpor_LOC101787990
roCpor_LOC101788169
roCpor_LOC101788198
roCpor_LOC101788018p
roMaur_Leutx
roCgri_I79_012385
roCgri_I79_024957
roNlep_A6R68_07039
lagOpri_AAYZ01197513
lagOcun_LEUTX
lagOcun_LOC103348417
lagOcun_LOC103348415
lagOcun_LOC103347612
lagOcun_LOC103348423
lagOcun_AAGW02026615
roPman_AYHN01138932
roPman_Leutx
roMmus_Leutxp
roRnor_Leutxp
prHsap_LEUTX

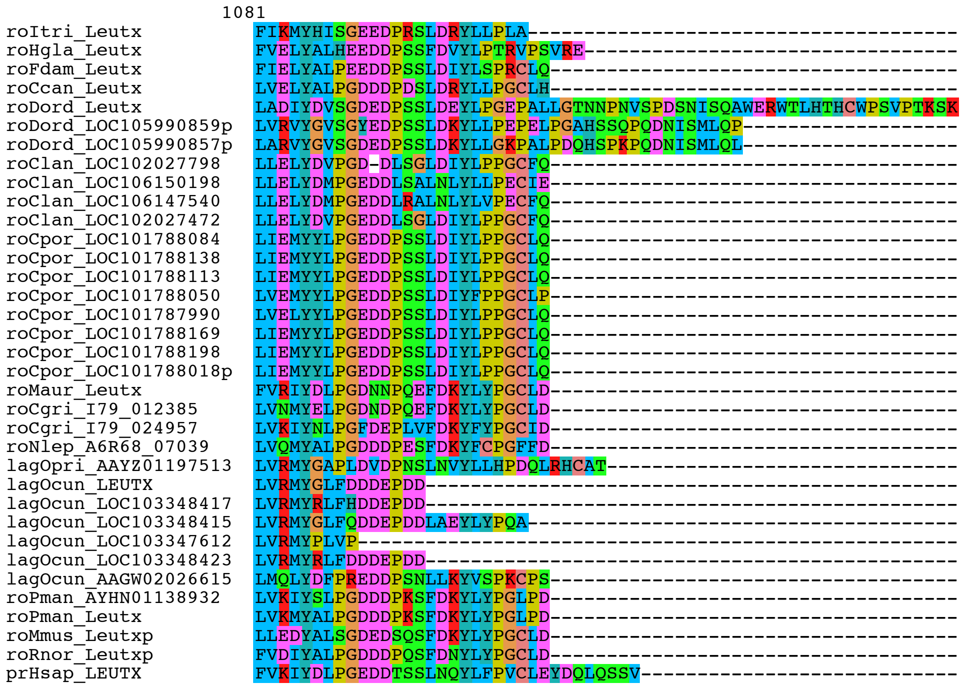

Fig. S2. Multiple sequence alignment of rodent and lagomorph Leutx proteins and human LEUTX. Several pseudogenes (postfix “p”) are included in this alignment, including rat and mouse. Guinea pig roCpor_LOC101788018p is located within a tandem repeated Leutx gene locus, containing 8 Leutx genes, which are located right at the edge of the contig; while its homeobox has the two typical splice sites, the first upstream exon could not be located within the sequenced region to the next Leutx ORF based on synteny, hence the designation of LOC101788018p as pseudogene. In the case of the prairie deer mouse Pman sequences, the contigs ended in the repetitive sequence region upstream of the Leutx domain, no Leutx homeoboxes were recovered, though they may be located in the missing upstream regions. Three of the chinchilla ORFs are in a tandem duplicated locus. Four of the rabbit ORFs are also in a tandem duplicated locus. For several not all exons have been retrieved. AAGW02026615 seems to encode only the C-terminal part of the Leutx domain, no splice and homeobox could be detected in the upstream region, it is possibly a pseudogene. The American pika ORF has 5 repeats of about 40 residues between the homeodomain and the Leutx domain.

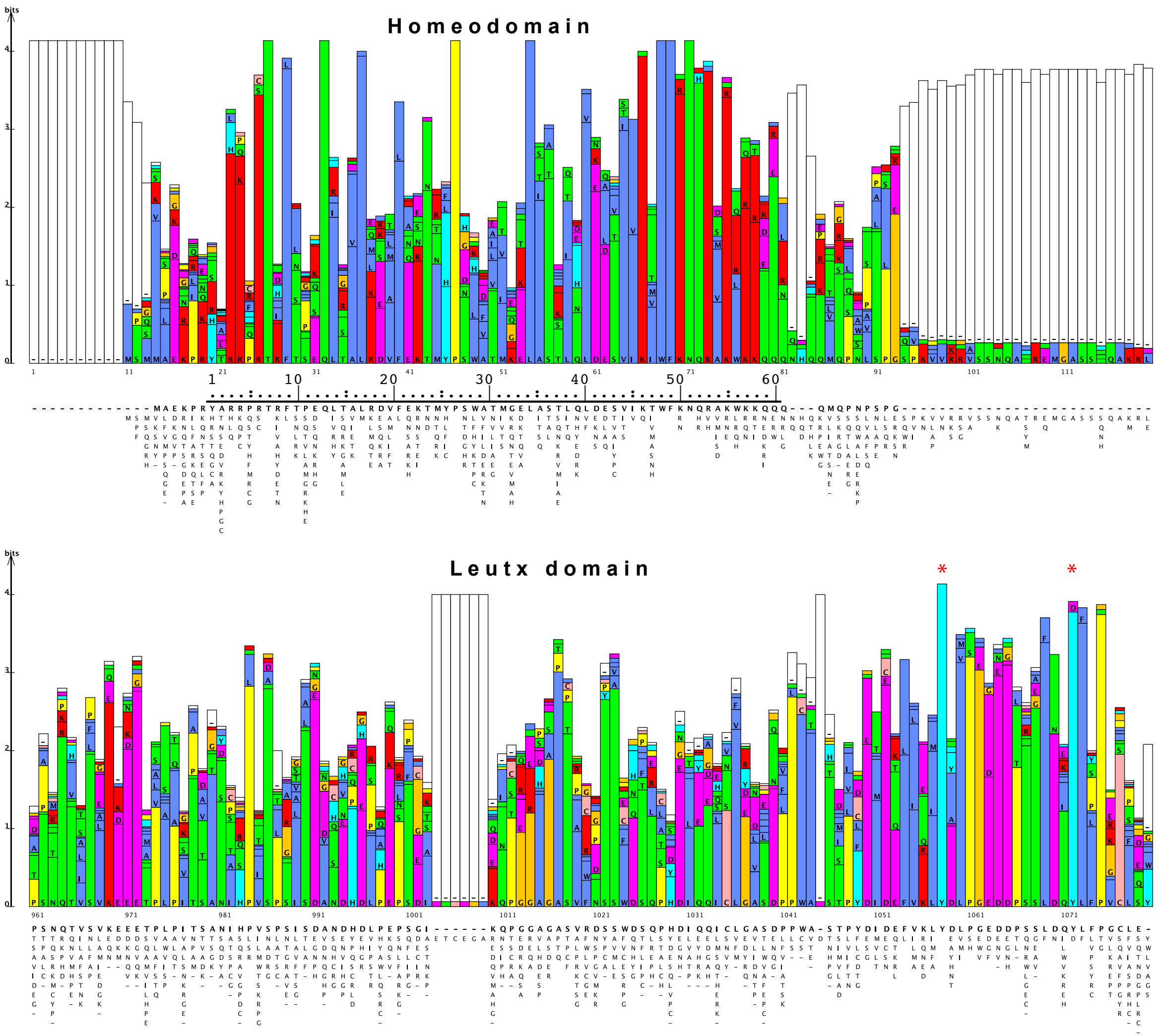

Fig. S3. Protein logo of the Leutx homeodomain and the Leutx domain. The homeodomain is indicated. Red asterisks mark highly conserved tyrosine residues.

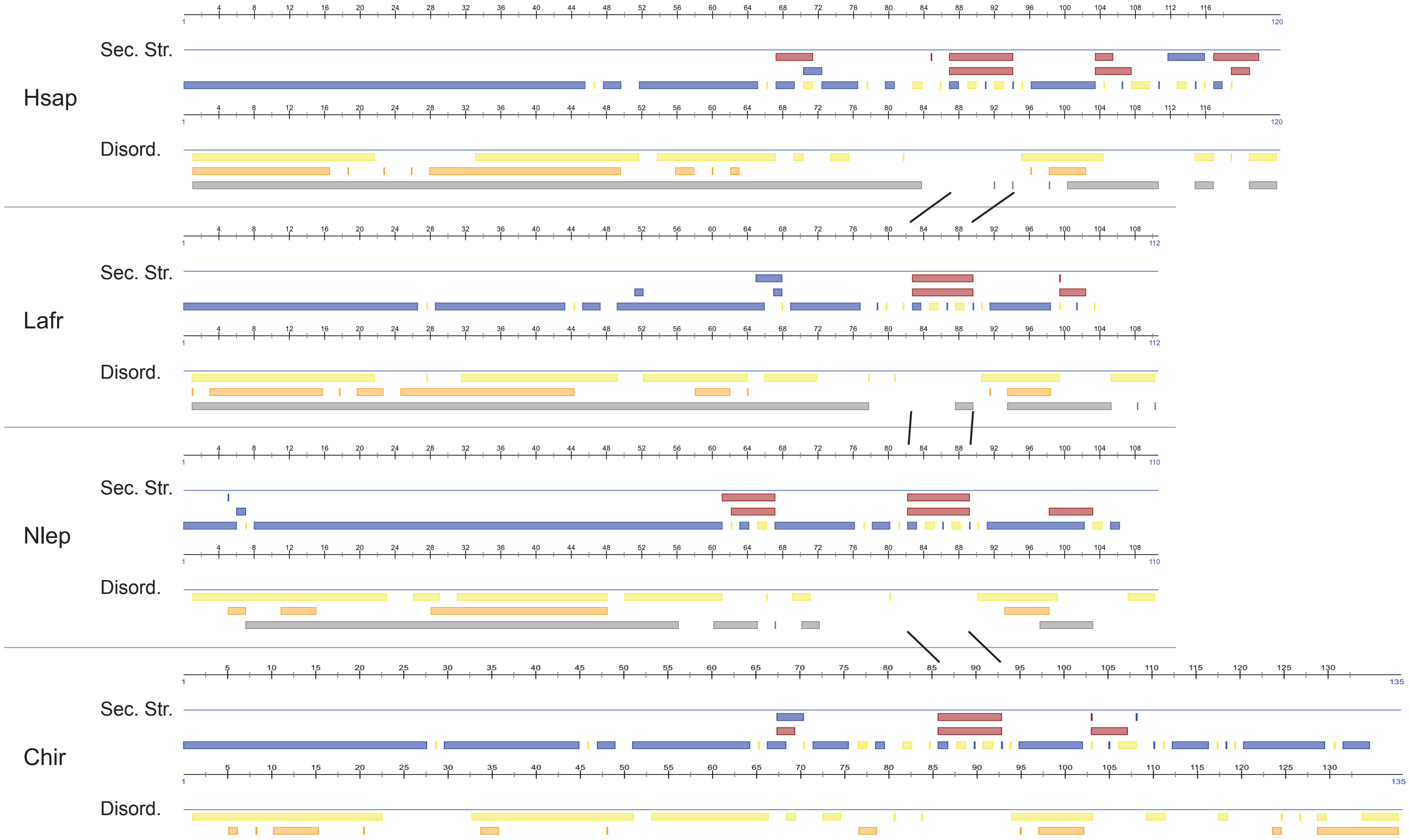

Fig. S4. Predictions for secondary structure and disorder. Four different LEUTX protein sequences (indicated on the left) were used to initiate the prediction at the PredictProtein server. Although a multiple sequence alignment is generated, the results were slightly different for the four sequences, probably due to different alignments produced. Secondary structure prediction (Sec. Str.), top line: REPROFSec, second line: PROFsec, (red = helical, blue = strand), third line: PROFAcc (accessability: blue = exposed, yellow = buried). Protein disorder prediction: top line: PROFbval (yellow = disordered), second line: Ucon (orange = disordered), third line: NORSnet (disordered = grey). Note most of the Leutx domain is predicted to be disordered and exposed, while only one region is consistently predicted to be helical (human 162 - 169, here around residues 85 - 90).

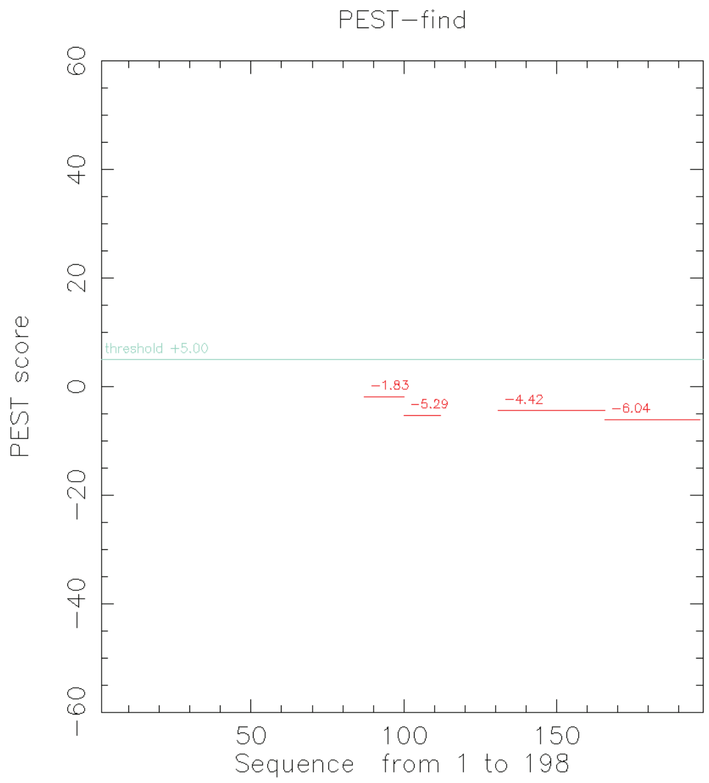

Fig. S5. PEST sequence prediction using epestfinder of the human LEUTX protein. Four putative PEST region were predicted, although with low scores below the threshold. LEUTX sequences from other species gave similar results, in some cases with signficant scores.

```
                             961                                                                                        *               *
prHsap_LEUTX                   PANQTTSVKKEETPSAITTANIRPVSPGISDANDHDLREPSGI------KNPGGASASARVSSWDSQSYDIEQICLGASNPPWA-STLFEIDEFVKIYDLPGEDDTSSLNQYLFPVCLEY
prPpan_LEUTX                   PANQTISVKKEETPSAITTANIRPVSPGISDANDHDLREPSGI------KNPGGASASARVSSWDSQSYDIEQICLGASNPPWA-STLFEIDEFVKIYDLPGEDDTSSLNQYLFPVCLEY
prGgor_LEUTX                   PANQTISVKKEETPSAITTANIRPVSPGISDANDHDLREPSGI------KNPGGASASARVSSWDSQSYDIEQICLGASNPPWA-STLFGIDEFVKIYDLPGEDDTSSLNQYLFPVCLEY
prPabe_LEUTX                   PANQTISVK-EETPSAITTANIRPVSPGIFDANDHDLREPSGI------KNPGGASASARDSSWDSQSYDIEQICLGASNPPWA-STLYEIDEFVKIYDLPGEDDTSSLNQYLFPVCLEY
prNleu_LEUTX                   PANQTISVKKEETPSAITTANIHPISPGISDANDRDLYEPSGI------KNPGGAGASLRDSSWDSQSYDIEQLCLGASNPPWA-STLYEIDEFVKIYDLPGEDDTSSLNQYLFPVCLEY
prMleu_LEUTX                   PSNQTLSVKKEETPSAITTANIRPVSPRISDANDHDLHEPSDI------KNPGGAGASVRDSSWDSRAHDIEQICLGASNPPWA-STVCEIDEFVKIYDLPGEDDTSSLNQYLFPVCLEY
prCaty_LEUTX                   PSNQTLSVKKEETASAITTANIRPISPRISDANDHDLHEPSDI------KNPGGAGASVRDSSWDSRAHDIEQICLGASNPPWA-STVCEIDEFVKIYDLPGEDDTSSLNQYLFPVCLEY
prPanu_LEUTX                   PSNQTLSVKEEETPSAITTANIRPVSPRISDANDHDLHEPSDI------KNPGGAGASVRDSSWDSRAHDIEQICLGASNPPWA-STICEIDEFVKIYDLPGEDDTSSLNQYLFPVCLEY
prMfas_EGM_09724               PSNQTLSVKEEETPSAITTANIHPVSPRISDANDHGLHEPSDI------KNPGGAGASVRDSSWDSRAHDIEQICLGASNPPWA-STVCEIDEFVKIYDLPGEDDTSSLNQYLFPVCLEY
prMmul_LOC699783               PSNQTLSVKEEETPSAITTANIHPVSPRISDANDHGLHEPSDI------KNPGGAGASVRDSSWDSRAHDIEQICLGASNPPWA-STVCEIDEFVKIYDLPGEDDTSSLNQYLFPVCLEY
prCang_LEUTX                   PSNQTLSVKEEESPSAITTANIHPVSPGISDANDHDLHEPSDI------KNPGGASASVRDSSWDSRAHDIEQICLGASNPPWA-STVCEIDEFVKIYDLPGEDDTSSLNQYLFPVCLEY
prCsab_LEUTX                   PSNQTLSVKEEETPSAITTANIRPVSPGISDANDHDLHEPSDI------KNPGGAGASVRDSSWDSQAHDVEQICLGVSNPPWA-STVCEIDEFVKIYDLPGEDDTSSLNQYLFPVCLEY
prRrox_LEUTX                   PSNQTLSVKEEETTSAITTANIHPVSPGISDANDHDLHEPSDI------KNPGGAGTSVRDSSWDSQAHEIEQIYLGVSNPPWA-STVDEIDEFVKIYDLPGEDDTSSLNQYLFPVCLEY
prAnan_LEUTX                   ASSQTISVK-EETPSAVATADIHPISPRISDVNDHDLHEPSGI------KNPGEAGASVRDSSWHSQSHDIEQICLGASNLPWA-STPYEIDEFIKIYDLSEEDGTSSLNEYLFPPSVSG
prCjac_LEUTX                   ASSRTISVK-EETPSAVTTADIHPVRPRISDVNDHDLHEPSGI------KNPGEASASVRDSSGHSQSHDIEQICLGASDLPWA-SAPYEIDEFVKIYDLSEEDDTSSLNQYLFPPSVPG
prSbol_LEUTX                   ASRQTISAK-EETPSALTTADIRPISPRISDVNDHDLHEPSGI------KNPGEAGASVRNSSWHSQSHDIEQICLGTSNLPWA-STPYEIDEFVKIYDLSEEDDTSSLNQYLFPPSVPG
euTchi_LEUTX                   -----APDQMEGTPILRTAANTPPVSLGVSDANDHDPSEPSDI------KSPEGAGASLRNSSRDSQPSDIEQICLEASDPLWV-SIPYDMDEFVQLYALPGDEDPGSLDQYLSPGYLG-
euGvar_LEUTX                   ISNQTFSVKEEETPLPITSANTCPMSPEILNDCDHDSCEPSDI------RQRGEADASVCNSSWDSQPYDIEQICLEASDPPWA-TIPYEIDLFVQLYALPWEDDPSSLDQYLRPGCLD-
laOros_LEUTX                   --NPTIPGKEEGESLPVTSANSHLKSPSISDASDHELPQSPEN---------DEPGPSRGNSSWDSQPSDLQERCLGDSDPPWA-SSPYDIDQFIQLYALPGDDDPSSLDQYLPPKCRS-
laLwed_LEUTX                   --NPTVPGKEEEESLPVTSANTHRKSPSISDASDHELAQFSEN---------DEPDPSRGNSSWDSQPSDLQEICLGDSDPPWA-SSPYDIDQFIQLYALPGDDDPSSLDQYLSPKCCS-
laClup_AOCS01144808            SSNPTISGQEEEPSLTVTSANTHPRSPNFLDACDHELPQSSDN---------GQPGPSRWGSSWYSLPPDLQQICLGDSDPLWA-SSPYDIDQFMELYALPGDDDPRSLDQYLSPTCLG-
laFcat_LEUTX                   --NQTISIKEEQPPLPVTSANTHPGSPSILDACDHELPQSPDS------VQSAGAGPSKWNSSWDSPPYDLQQICLGDPDPPWA-SIPYDIDQLIQLYALPGDDD---LDQYLSPRCPSS
laBtau_LEUTX                   DPRRVVSVKEEEMPLPGTSGSTHPTSLSLAGDSHHELPELSCA------EQCEGAAATPCPSSCNFLT----ALSLRDTDLPWA-SSPYDMDQLIQLYDLPGDDDPSSLDQYLFPECSSW
laBmut_M91_07100               DPRRVVSVKEEEMPLPGTSGSAHPTSLSLAGDSHHELPELSCA------EQCEGAAATPCPSSCNFLT----ALSLRDADLPWA-SSPYDMDQLIQLYDLPGDDDPSSLDQYLFPECSSW
laBbub_LEUTX                   DPRQVVSVKEEEMPLPGTSGSIHPTSLSLAGDSHHELPKLSCA------EQCEGAAATPCPSSCNFLT----ALSLRDADPPWA-SSPYDMDQLIQLYHLPGDDDPSSLDQYLFPECSSW
laChir_LEUTX                   DPCQVVSVEEEEMSLPGTSGSTHPTSLSLADDSHHELPELSCA------QQCEGAAATPCPSSCNFLTVHLHQISLGDSDPPWA-SSPYDMDQLIQLYDLPGDDDPSSLDQYLFPECSSW
laPhod_LEUTX                   DPCQVVSVEEEEMPLPGTSGSTHPTSLSLADDSHHELPELSRA------QQCEGAAATPCPSSCNFLTVHLHQISLGDSDPPWA-SSPYDMDQLIQLYDLPGEDDPSSLDQYLFPECSSW
laCfer_LEUTX                   DLQQVSSVEEEEASSPATSANSRPTGLSISDASDHEPPKSGI-------EQPQAAGATPCSSSCGLLPDDLQQINFRDFDLPWA-STPYDMDQFIQLYALPGGDGPWSLDQDLFPECSD-
laVpac_LEUTX                   DPQQVSSVEEEEASSPATSANSRPTDLSISDASDHEPPKPGI-------EQPQAAGATPCSSSCGLLPDDLQQINFRDFDLPWA-STPYDLDQFIQLYALPGGDGPWSLDHDLFPECSD-
laSscr_LEUTX                   DTPQVSPVKEEETLSPSASGNTRSMSLSISDASDPDSPQPSCAETCEGAAPCEGAAATPCDSSCDFLPEDLRQISFRDPDPPWA-SSPYDMDQLIQLYNLPGEDDPSSLDQYLLPLCST-
laEcab_AAWR02030248            GPNQTTSAKEEEAPLPVTSAATHLTSPAVSDDCGHEPPKPSGI------EQPGGAGASVWN-SWDPQLQNLQQICLGVSDPPWA-SVPTDMDEFIQLYALPGDDDPSSLDEYLFPKGPS-
laEasi_JREZ01000468            GPNQTTSAKEEEAPLPVTSAATHLTSPAVSDDCGHEPPKPSGI------EQPGGAGASVWN-SWDPQLQDLQQICLGVSDPPWA-SVPTDMDEFIQLYALPGDDDPSSLDEYLFPKGPS-
laCsim_LOC101387510            APNQTTSVKEQETLLPVTSANTDPTSPSISDDCEHEPPKPSGI------QQPGGAGASVWN-SWDSQSHDLQQICLGLSDPPWA-SVPYDMDEFIRLYTLPGDDDPSSLDWYLFPKCPN-
laPale_PAL_GLEAN10001506       APNQTISVKEEETPSPVASTDTHPVS----EARDRDPPKAPGI------EQPGGAGASAWNASWDSQPHDLQEMCLEASDIPWA-TMPYDIDQFIRFYALPGDDHPNSLDQYLFPKQPG-
laMjav_LEUTX                   APYKTASVKEEDSPSAEMSANTSPTSLSISDHCDRESPEPSGI------EQPRRVGASGGNSSGDPQPSDLQQICLGVSDAPWA-SIPYDIDQFIQAYALPVDDDPCGLDQYLLPKPPGW
afLafr_LEUTX                   EPNHVAPVKKEEMPLPITDANPGPVSARSLDASEPDPPEPPGA------GQPGGAGACVWNSSWDSHTHDAQEINLDILDPPWA-TIPCDIDEFVELYALPGDDDPSSLDQYLLPRCPC-
afLafr_LEUTX2                  EPNQVAPMKKEEIPLPITDANPGPVSARSLDASEPDPPEPPGA------GQPGGAGACVWNSSWDSHTHDAQEINLDILDPPWA-TIPCDIDEFVELYALPGDDDPSSLDQYLFPKAHLL
xeDnov_LEUTX                   APTKTVSVKEEETPLAITDAQS-PPSPGISDPRNPETPEGSGS------EHPKGAGASAENSSWNSRPCDLQDKC-----PPWA-SILCDVERFVELYDLPGEVDPSELDQYLFPERLRA
roItri_Leutx                   PSTQNTAENEVVSPAPVNSKNGVPCDSATTHHQDDGVPSS------------SGLGQPVLDSERDCLPSDLKDICLRVSDSPWA-NTTLDMDQFIKMYHISGEEDPRSLDRYLLPLA---
roHgla_Leutx                   TSTQQVLEE-KEPPLQNPATNASPIA-GSSDDGNHDPQEPSES------HITRRDGAPVLSSAVDTPHDNVQDTCMGDLDVPWA-HTPGDISEFVELYALHEEDDPSSFDVYLPTRVPSV
roFdam_Leutx                   TSTQHVLEK-EEAALPNPKSNASPIA-GTSEDGNHEP---SES------HSTRRHEAPAWRPAVDSQCDDIQHICLQDWDVPWA-GTPGDINQFIELYALPEEDDPSSLDIYLSPRCLQ-
roDord_Leutx                   APQDQAN---NKMQV--------PWACALSPQQPSRIWVPQGP------EQAQQAAASSFPPASQ---------SVDDLEVSWTDPLPYSTEELADIYDVSGDEDPSSLDEYLPGEPALL
roClan_LOC102027798            TSMKHVLEK-EETPLTKPATNTSPSS-AASDGHNLHPQEPSDT------QITGRDGASAFPSSPHIQCHDIQEESLENCGTPCT-HDFFDTCQLLELYDVPGD-DLSGLDIYLPPGCFQ-
roClan_LOC106150198            TSMQHVFKK-EEAPWPKPATNPAPSS-AASDGQNPHPQEPSDA------QITGRDGASAFPSSSHTQCHEIQEESLENWDKPCT-HDLLDTCQLLELYDMPGEDDLSALNLYLLPECIE-
roClan_LOC106147540            TSTRHVFEK-EEAPLPKTAANPAPSS-TASDGQNPHPQEPSDT------QITERDGASAFPSSSHPQCYDIQEESLENWDKPCT-HDLLDTCELLELYDMPGEDDLRALNLYLVPECFQ-
roClan_LOC102027472            TSTQHVLEK-EEAPLTKPATNPAPSS-AASDGHNPHPQEPSDT------QITGRDGASAFPSSPHIQCHDIQEESLENCGTPCT-HDFFDTCQLLELYDVPGEDDLSGLDIYLPPGCFQ-
roCpor_LOC101788084            TSMQTSSEK-KEQVLQSPVASCSPIS-SISDDHVCDRAERFEK------DISRRHRASVFGSSFQSQPDGTQGHVVYVEDF-----DIDDTDKLIEMYYLPGEDDPSSLDIYLPPGCLQ-
roCpor_LOC101788138            TSTQTSSEK-KEHVLQSPVASCSPIS-SISDDHVCDRAERFEK------DISRRHGASVFGSSFQSQPDGTQGHVVYVEDF-----DIDDTDKLIEMYYLPGEDDPSSLDIYLPPGCLQ-
roCpor_LOC101788113            TSTQTSSEK-KEHVLQSPVASCSPIS-SISDDHVCDRAERFEK------DISRRHGASVFGSSFQSQPDGPQGQ--YVEDF-----DIDDTDKLIEMYYLPGEDDPSSLDIYLPPGCLQ-
roCpor_LOC101788050            TSTQTSSEK-KEQVLQSPVASCSPIS-SISDDHVYDRAERFEK------DISRRHGAFVFDSSFQTQPVEIQGQ--YVEDF-----DIDDTDKLVEMYYLPGEDDPSSLDIYFPPGCLP-
roCpor_LOC101787990            TSTQPSLEQ---EAPQNPVTSPSPIS-VTSNDHIHDPLELLES------EITRREGTSVFGSSFDTQCDNIQGQ--YVEDF-----ANLDTDKLVELYYLPGEDDPSSLDIYLPPGCLQ-
roCpor_LOC101788169            TSTQTSSEK-KEHVLQSPVASCSPIS-SISDDHVCDRAERFEK------DISRRHGASVFGSSFQSQPDGTQGHVVYVEDF-----DIDDTDKLIEMYYLPGEDDPSSLDIYLPPGCLQ-
roCpor_LOC101788198            TSTQTSSEK-KEHVLQSPVASCSPIS-SISDDHVCDRAERFEK------DISRRHGASVFGSSFQSQPDGTQGHVVYVEDF-----DIDDTDKLIEMYYLPGEDDPSSLDIYLPPGCLQ-
roMaur_Leutx                   SSKQVKSLKDDGILAPKAD-YAQLLSTGISDNSDQESWKNFGT------MQPEESGASAGKYVEHSELLDIYQSSLGLAQPPWA-SMPFDIDTFVRIYDLPGDNNPQEFDKYLYPGCLD-
roCgri_I79_012385              ASMQEKSLKDNEVFAPKAD-SAQLLSTRGSD-SNQESWKKIGA------KQPEESGASVRKYVEHSELLDIYQSNLGPVQPPWA-SMPFDIDTLVNMYELPGDNDPQEFDKYLYPGCLD-
roCgri_I79_024957              PSKQMISLKDDEVFAPKAD-YAQLLNTNGSDNSDQESWKNFGA------KQPEESGASVGKCMEYSELLDIYQSSLGLVQPPSE-SMPFDIDTLVKIYNLPGFDEPLVFDKYFYPGCID-
roNlep_A6R68_07039             VCKQVNFLKDDELLAPKTD-YAQLLSTRGSDNSGQES-QNFGP------KQPEEAGASTVMSLEHSELLELYQTCLGLVQPPWA-SLPFDIDTLVQMYALPGDDDPESFDKYFCPGFFD-
roMmus_Leutxp                  PSKQTQSLKDDQIPTPTAD-CAHLLNPKSSDNSVQESQKNFGA------KKPEEAGTSD------SQLLEVDQICLGHSPPPWA-SMPFDIDTLLEDYALSGDEDSQSFDKYLYPGCLD-
roRnor_Leutxp                  PHKQNKSWKDDQILDT------------SSDSSDQESQTNFGA------EKPEGAGISE------PQLLDSHQSCLGRSQPPWA-SVPCSIDTFVDIYALPGDDDPQSFDNYLYPGCLD-
```

Fig. S6. Prediction of 9aaTAD transactivation domains. Sequences marked blue were analysed at the 9aaTAD prediction tool server. Regions highlighted show matches to the 9aaTAD motif. Red: most stringent match, orange: moderately stringent match, yellow: less stringent matches. Underneath the sequences are predicted alpha helixes (Figure S3). Dark blue marks a consistently identified helix, light blue less consistently identified helixes. The magenta asterisks indicate highly conserved tyrosine residues.

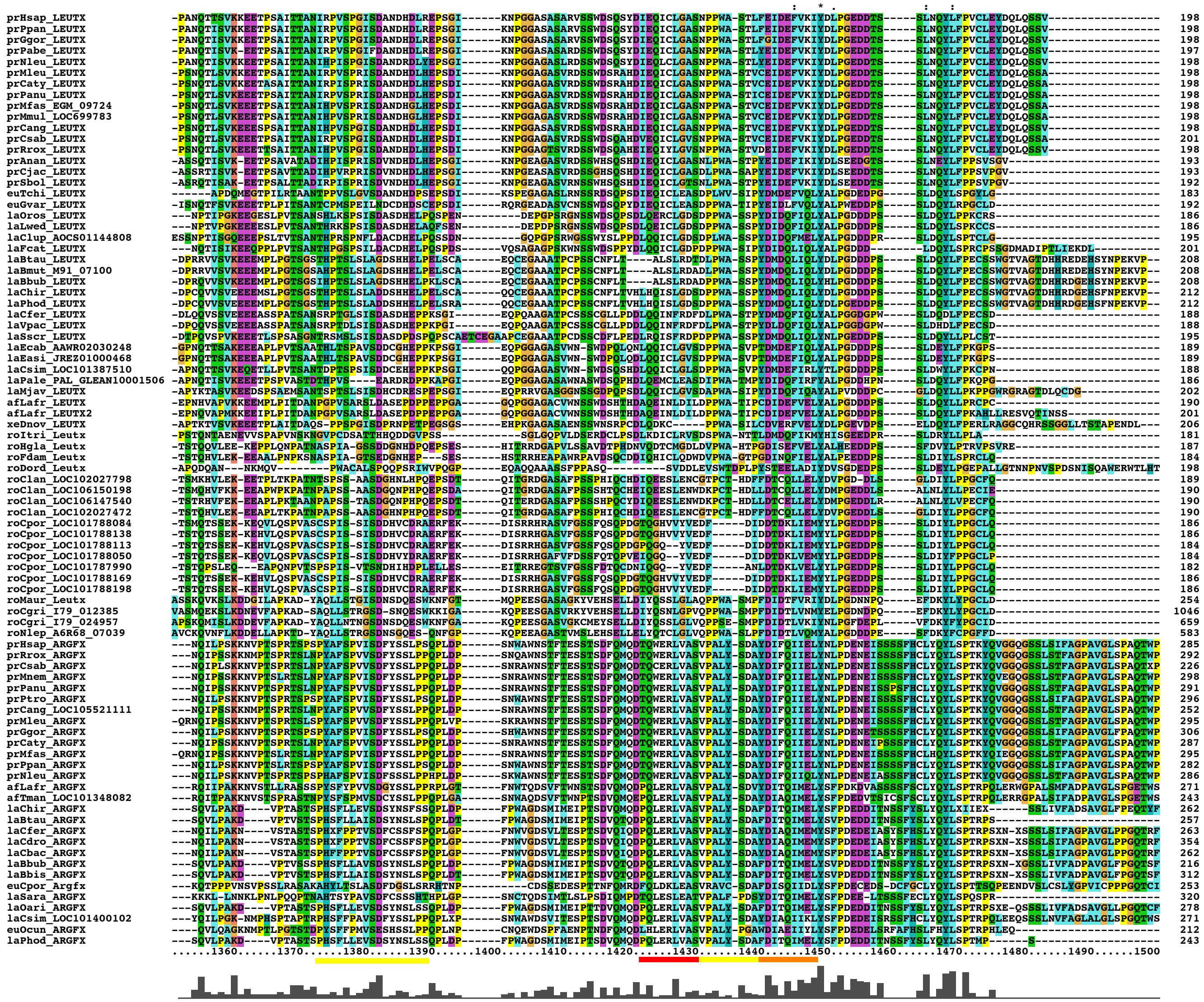

Fig. S7. Sequence alignment of the carboxy-terminal region of Argfx proteins and Leutx domains. 9aaTAD prediction for human ARGFX is shown underneath the alignment (color coded as in Figure S6).

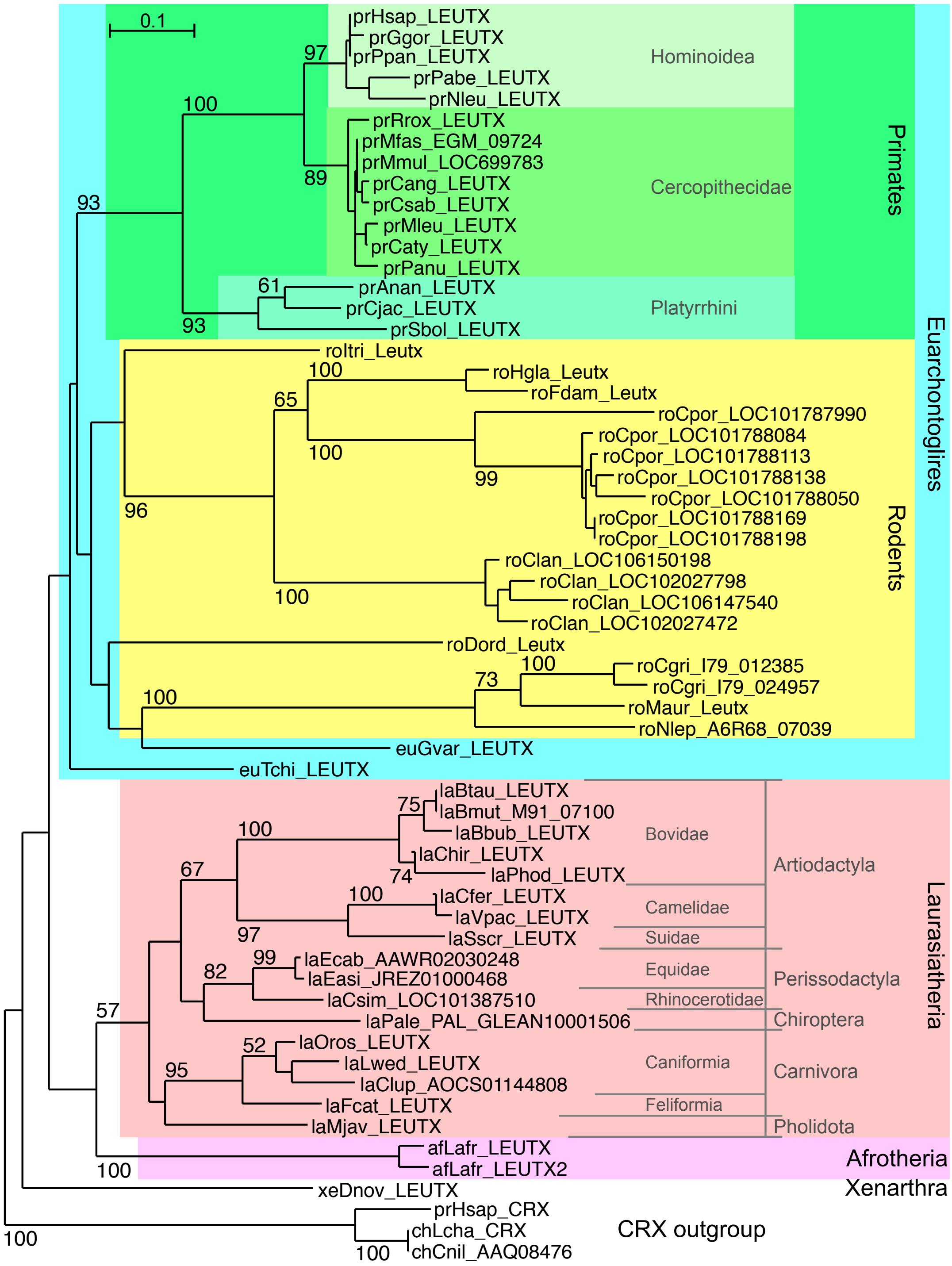

Fig. S8. Neigbour joining tree of the homeodomain region as in Figure 3. Locus identifiers (Table S2) were used here instead of species names to allow for easier comparison with trees in Figures S9 - S11.

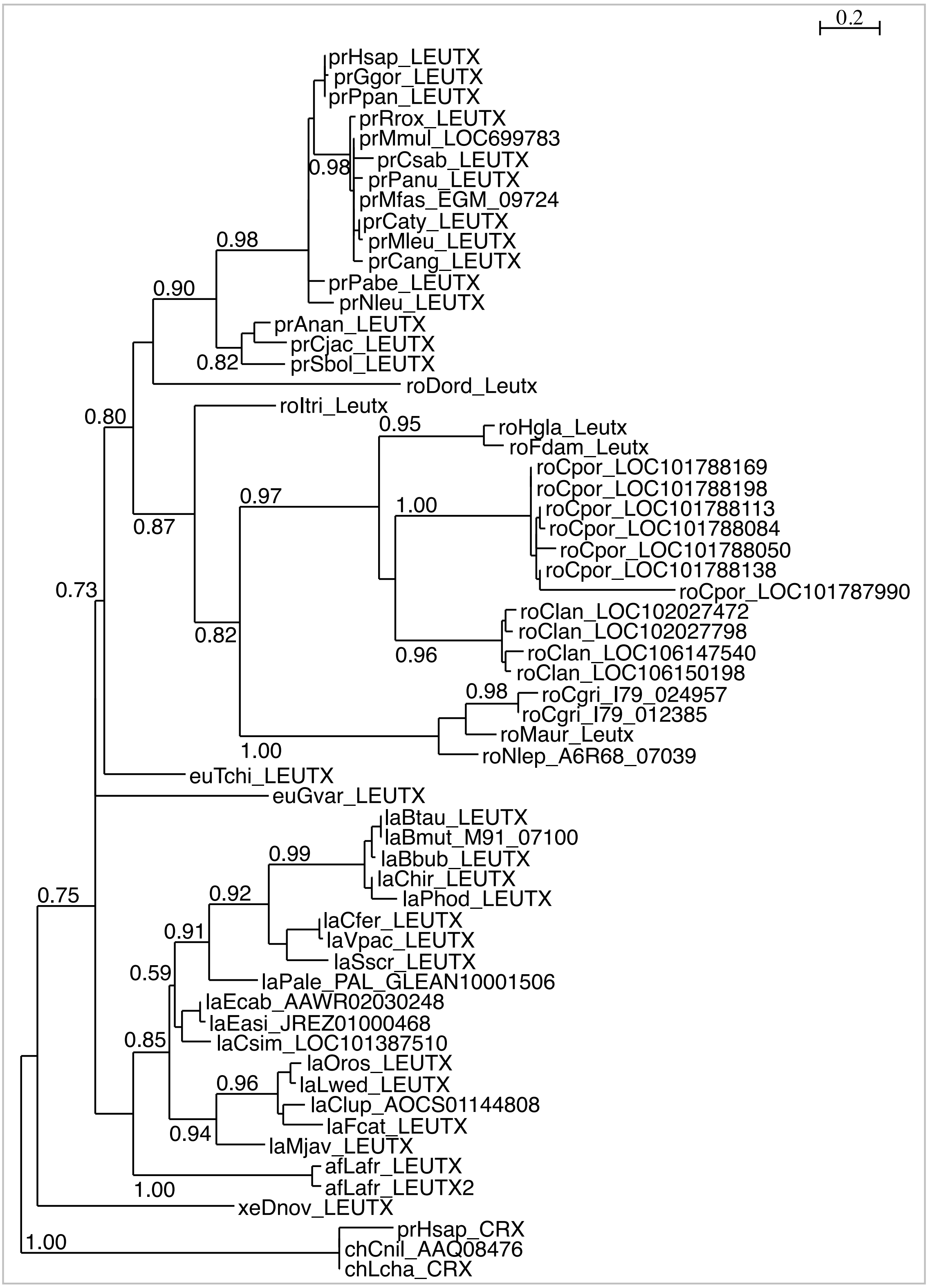

Fig. S9. Maximum likelihood phylogenetic tree (PhyML) of the homeodomain region. aLRT branch support is shown, with values less than 0.5 and near end nodes omitted.

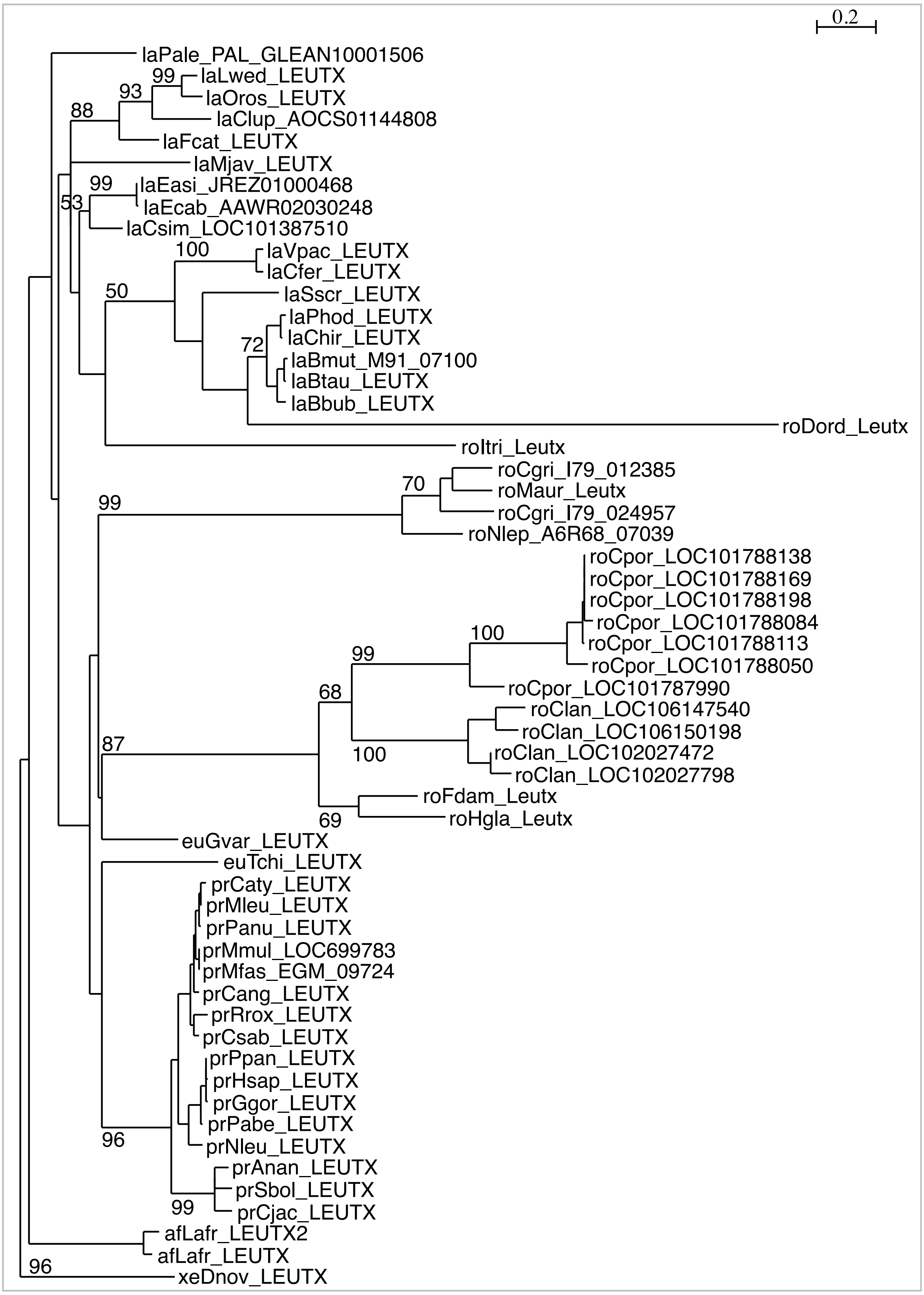

Fig. S10. Maximum likelihood tree (PhyML) of the Leutx domain. *D. novemcinctus* was used as outgroup. Bootstrap values (100 trials) less than 50 and near end nodes have been omitted.

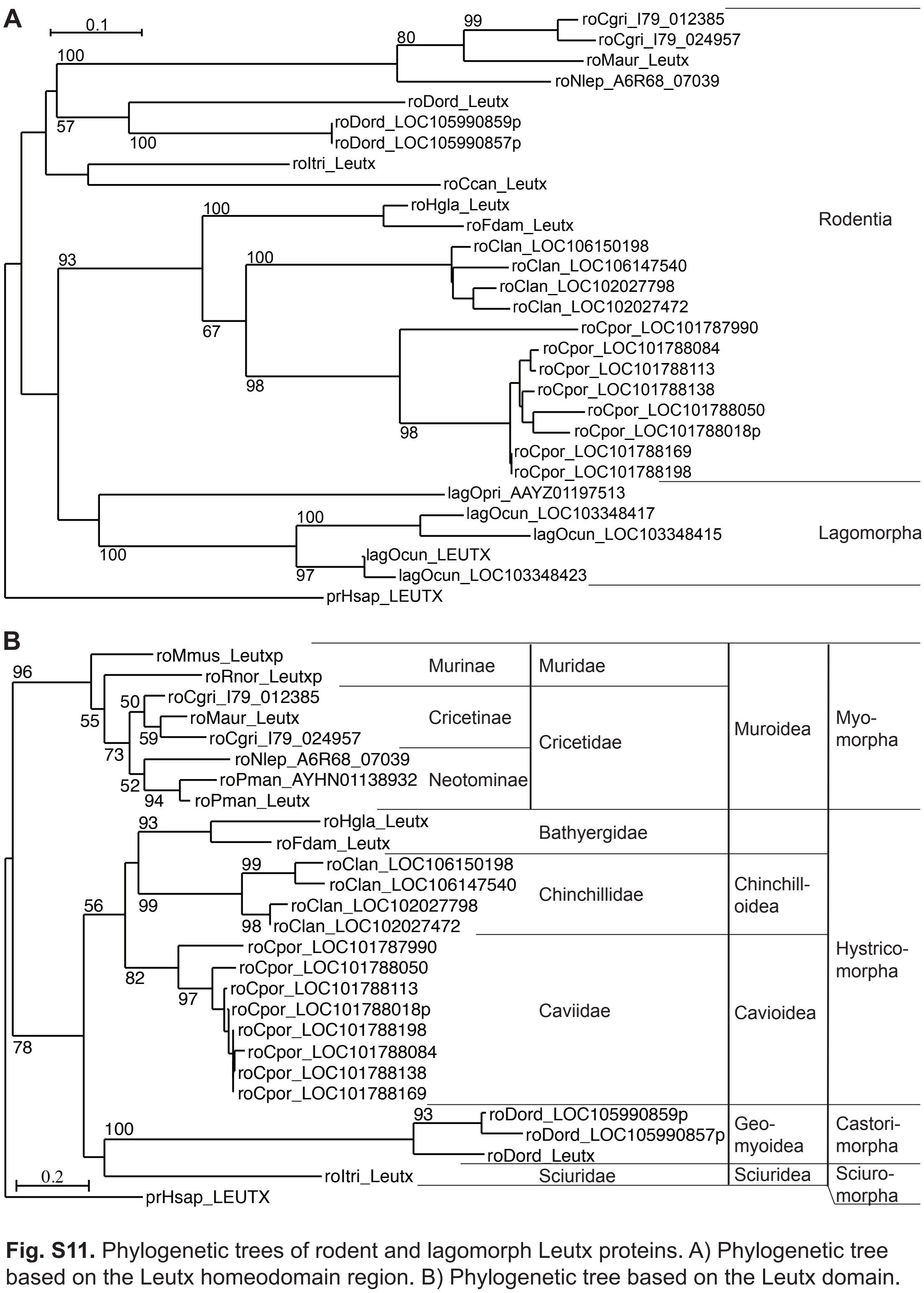

Fig. S11. Phylogenetic trees of rodent and lagomorph Leutx proteins. A) Phylogenetic tree based on the Leutx homeodomain region. B) Phylogenetic tree based on the Leutx domain. Taxonomic classification of the species in the tree is shown on the right. Lagomorpha were omitted due to their partial Leutx domain. In both trees, human Leutx was used as outgroup. BioNJ as implemented in SEAVIEW was used for tree generation. Bootstrap (100 replicates) values lower than 50 and nodes near leafs (for legibility) were removed. Phylogenetic tree analysis using PhyML resulted in trees with similar topology (data not shown).

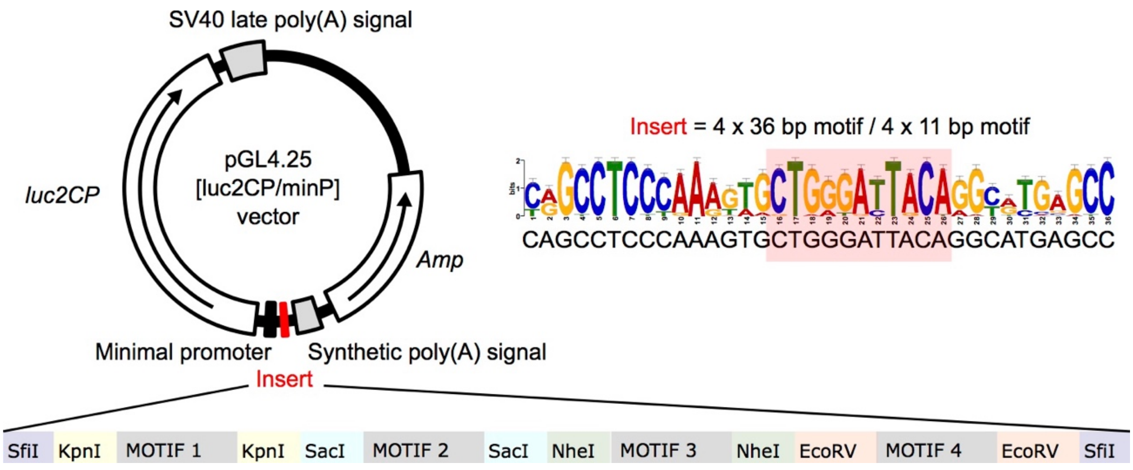

Fig. S12. Luciferase reporter vector with the insert structure containing four copies of the 11 bp core motif (marked in pink in the 36 bp EEA motif). The four repetitive motifs are surrounded by KnpI, SacI, NheI and EcoRV restriction sites, respectively.

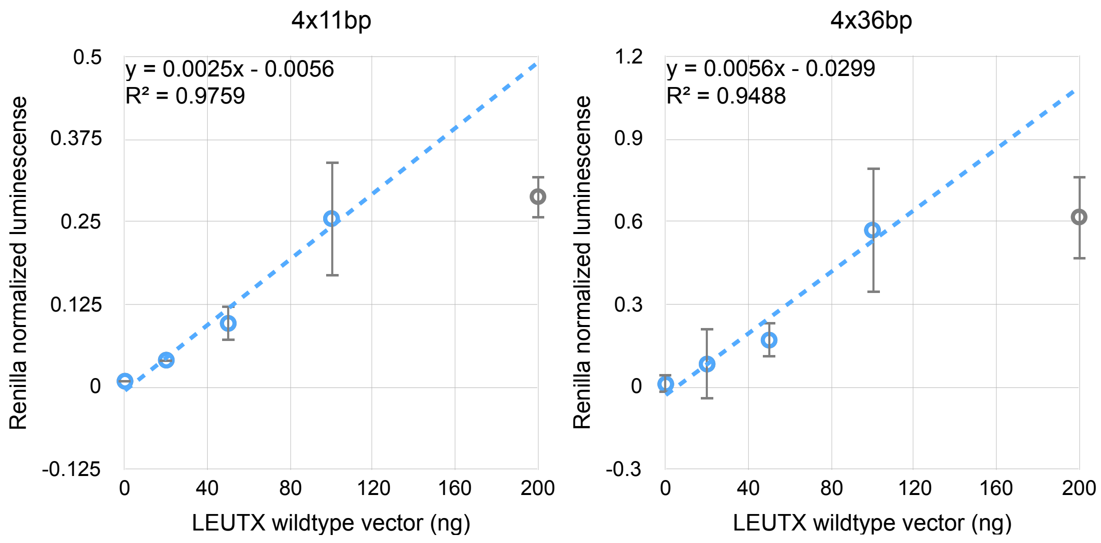

Fig. S13. Renilla normalized luminescence (RNL) with different vector concentrations and promoters. To estimate the most appropriate concentration of the TF expression vector, we measured RNLs at five different concentrations (0, 20, 50, 100 and 200 ng) of wild-type LEUTX using the two promoters. The values were significantly correlated up to 100 ng for both promoters (r=0.99 and 0.95; p=0.01 and 0.03, respectively). At 200 ng, the RNL values were saturated, because of accumulation of renilla luminescence levels. Based on these result, we used 100 ng of TF expression vector for all experiments in this study. The shown regression lines (blue dashed lines) use the lower four concentrations (blue points) only. Vertical bars at each point represent the S.E.M.

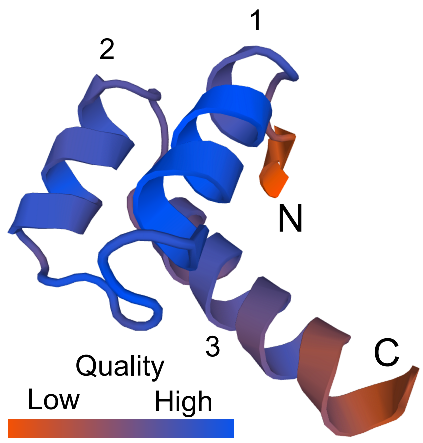

Fig. S14. Quality of the initial model (PDB: 2HDD, chain A). LEUTX shares 40% sequence identity with the template structure (2HDD), but residues recognizing DNA are nearly totally conserved. Due to missing structural data in the PDB entry 2HDD at the N-terminus, a structure of human distal-less homeobox protein 5 (4RDU, chain D) was used for modelling residues 1-4 of the LEUTX homeodomain. Coloring shows the local lDDT values describing the expected quality scores ranging from 0.42 to 0.95. The three helixes are numbered by the order.

Gene-Locus-name	Accession Nr	Gene family	Manual changes	RT	Seq. length	Sequence
prHsap_LEUTX	NP_001137304	Leutx			198	MFEGPRRYRRPRTRFLSKQLTALRELLEKTMHPSLATMGKLASKLQLDLSVVKIWFKNQRAKWKRQQRQQMQTRPSLGPANQTTSV<br>KKEETPSAITTANIRPVSPGISDANDHDLREPSGIKNPGGASASARVSSWDSQSYDIEQICLGASNPPWASTLFEIDEFVKIYDLP<br>GEDDTSSLNQYLFPVCLEYDQLQSSV
prPpan_LEUTX	XP_014198165	Leutx			198	MFEGPRRYRRPRTRFLSKQLTALRELLEKTMHPSLATMGKLASKLQLDLSVVKIWFKNQRAKWKRQQRQQMQTQPSLGPANQTISV<br>KKEETPSAITTANIRPVSPGISDANDHDLREPSGIKNPGGASASARVSSWDSQSYDIEQICLGASNPPWASTLFEIDEFVKIYDLP<br>GEDDTSSLNQYLFPVCLEYDQLQSSV
prGgor_LEUTX	XP_004060776	Leutx	yes		198	MFEGPRRYRRPRTRFLSKQLTALRELLEKTMHPSLATMGKLASKIQLDLSVVKIWFKNQRAKWKRQQRQQMQTQPSLGPANQTISV<br>KKEETPSAITTANIRPVSPGISDANDHDLREPSGIKNPGGASASARVSSWDSQSYDIEQICLGASNPPWASTLFGIDEFVKIYDLP<br>GEDDTSSLNQYLFPVCLEYDQLQSSV
prPabe_LEUTX	XP_002829268	Leutx	yes		197	MLEGPRRDRRPRTRFLPKQLTALRELLEKTMHPSLATMGKLASKLQLDLSIVKTWFKNQRAKWKRQWRQQMQPRPSLGPANQTISV<br>KEETPSAITTANIRPVSPGIFDANDHDLREPSGIKNPGGASASARDSSWDSQSYDIEQICLGASNPPWASTLYEIDEFVKIYDLPG<br>EDDTSSLNQYLFPVCLEYDQLQSSV
prNleu_LEUTX	XP_003270574	Leutx	yes		198	MLEGPRRGRRPRTRFLSKQLAALRELLEKTMHPSLATMGKLASKLQLDLCVVKTWFKNQRAKWNRQRRQQVKPWPPLGPANQTISV<br>KKEETPSAITTANIHPISPGISDANDRDLYEPSGIKNPGGAGASLRDSSWDSQSYDIEQLCLGASNPPWASTLYEIDEFVKIYDLP<br>GEDDTSSLNQYLFPVCLEYDQLQSSV
prMleu_LEUTX	XP_011839453	Leutx	yes		198	MLERPKQERRQRTRFLSKQLTALREVLAKTMHPSLVTMGKLASTLQLDLSVVKIWFKNQRAKWKRQQRQRMQPWPSLGPSNQTLSV<br>KKEETPSAITTANIRPVSPRISDANDHDLHEPSDIKNPGGAGASVRDSSWDSRAHDIEQICLGASNPPWASTVCEIDEFVKIYDLP<br>GEDDTSSLNQYLFPVCLEYDQLQSSA
prCaty_LEUTX	XP_011942061	Leutx	yes		198	MLERPKRERRQRTRFLSKQLTALREVLAKTMHPSLVTMGKLASTLQLDLSVVKIWFKNQRAKWKRQQRQRMQPWPSLGPSNQTLSV<br>KKEETASAITTANIRPISPRISDANDHDLHEPSDIKNPGGAGASVRDSSWDSRAHDIEQICLGASNPPWASTVCEIDEFVKIYDLP<br>GEDDTSSLNQYLFPVCLEYDQLQSSA
prPanu_LEUTX	XP_003915573	Leutx	yes		198	MLERPRGERRQRTRFLSKQLTALREVLAKTMHPSLVTMGKLASTLQLDLSVVKIWFKNQRAKWKKQQRQRMQPWLSLGPSNQTLSV<br>KEEETPSAITTANIRPVSPRISDANDHDLHEPSDIKNPGGAGASVRDSSWDSRAHDIEQICLGASNPPWASTICEIDEFVKIYDLP<br>GEDDTSSLNQYLFPVCLEYDQLQSSA
prMfas_EGM_09724	EHH59580	Leutx	yes		198	MLERPRRERRQRTRFLSKQLTALREVLAKTMHPSLVTMGKLASTLQLDLSVVKIWFKNQRAKWKRQQRQRMQPWPSLGPSNQTLSV<br>KEEETPSAITTANIHPVSPRISDANDHGLHEPSDIKNPGGAGASVRDSSWDSRAHDIEQICLGASNPPWASTVCEIDEFVKIYDLP<br>GEDDTSSLNQYLFPVCLEYDQLQSSA
prMmul_LOC699783	XP_001088235	Leutx	yes		198	MLERPRRERRQRTRFLSKQLTALREVLAKTMHPSLVTMGKLASTLQLDLSVVKIWFKNQRAKWKRQQRQRMQPWPSLGPSNQTLSV<br>KEEETPSAITTANIHPVSPRISDANDHGLHEPSDIKNPGGAGASVRDSSWDSRAHDIEQICLGASNPPWASTVCEIDEFVKIYDLP<br>GEDDTSSLNQYLFPVCLEYDQLQSSA
prCang_LEUTX	XP_011807838	Leutx	yes		198	MPERPRRERRQRTRFLSKQLTALREVLAKTMHPSLVTMGKLASALQLDLSVVKIWFKNQRAKWKRQQRQRMQPRPSLGPSNQTLSV<br>KEEESPSAITTANIHPVSPGISDANDHDLHEPSDIKNPGGASASVRDSSWDSRAHDIEQICLGASNPPWASTVCEIDEFVKIYDLP<br>GEDDTSSLNQYLFPVCLEYDQLQSSV
prCsab_LEUTX	XP_007994982	Leutx			201	MFGYMKRPRRERRQRTRFLSKQLTALREVLAKTMHPSLVTMGKLASMLQLDLSVVKIWFKNQRAKWKRQQRQRMQPWPSLGPSNQT<br>LSVKEEETPSAITTANIRPVSPGISDANDHDLHEPSDIKNPGGAGASVRDSSWDSQAHDVEQICLGVSNPPWASTVCEIDEFVKIY<br>DLPGEDDTSSLNQYLFPVCLEYDQLQSSA
prRrox_LEUTX	XP_010379193	Leutx	yes		198	MLESPRRKRRQRTRFLSKQLTALREVLAKTMHPSLVTMGKLASTLQLDLSVVKIWFKNQRAKWKRQQRQRMQPWPSLGPSNQTLSV<br>KEEETTSAITTANIHPVSPGISDANDHDLHEPSDIKNPGGAGTSVRDSSWDSQAHEIEQIYLGVSNPPWASTVDEIDEFVKIYDLP<br>GEDDTSSLNQYLFPVCLEYDQLQSSV
prAnan_LEUTX	XP_012316780	Leutx			193	MMPERPKRDRRRRTILLSQQLKALRKMFEETMYPSLAAMVTLASEQHLDLSVVKTWFKNQRAKWKKQQQQQMQPQLSLGASSQTI<br>SVKEETPSAVATADIHPISPRISDVNDHDLHEPSGIKNPGEAGASVRDSSWHSQSHDIEQICLGASNLPWASTPYEIDEFIKIYDL<br>SEEDGTSSLNEYLFPPSVSGV
prCjac_LEUTX	XP_003735618	Leutx	yes		193	MMPERPKHDRRHRTKLLSHQLEALRKTFEETMYPSLATMVKLASEQHLDLSVVKIWFKNQRAKWKKQQQQQMQPQLSLGASSRTI<br>SVKEETPSAVTTADIHPVRPRISDVNDHDLHEPSGIKNPGEASASVRDSSGHSQSHDIEQICLGASDLPWASAPYEIDEFVKIYDL<br>SEEDDTSSLNQYLFPPSVPGV
prSbol_LEUTX	XP_010329398	Leutx	yes		192	MMPERPKRVRRHRTTFLSQQLMALRKMFEKTTYPSLATMVTLASEQQLNLPVVQTWFKNQRAKRKKQQQKQMQPQLLLRASRQTIS<br>AKEETPSALTTADIRPISPRISDVNDHDLHEPSGIKNPGEAGASVRNSSWHSQSHDIEQICLGTSNLPWASTPYEIDEFVKIYDLS<br>EEDDTSSLNQYLFPPSVPGV
prMmur_LEUTXp	XP_012612322	Leutx	yes	RT	95	MSTIKSSKPKNTRRHCTNFSLEQLKILKEEFKKTMSPNWVTTEEQVSRLYLDESIIKTWFKNQCAKWKQQQQQQQTQASLSPGAS<br>DETISLIP*
euTchi_LEUTX	XP_006140461	Leutx	yes		183	MSVNPKRHRRCRTRFNPEQHRVLRDMFNKTMYPDWTTTEELASTLYLDESVVKIWFKNQRAKQKKQQQTQASLPPGAPDQMEGTPI<br>LRTAANTPPVSLGVSDANDHDPSEPSDIKSPEGAGASLRNSSRDSQPSDIEQICLEASDPLWVSIPYDMDEFVQLYALPGDEDPGS<br>LDQYLSPGYLG
euGvar_LEUTX	XP_008580451	Leutx	yes		192	MSGVVPARTRNRQTRTAFRPEQLRKLREMFKKTMFPDKITIQDLASVLHLNQSVIKTWFKNQRVRWKKQQQQTQPSFSPGISNQTF<br>SVKEEETPLPITSANTCPMSPEILNDCDHDSCEPSDIRQRGEADASVCNSSWDSQPYDIEQICLEASDPPWATIPYEIDLFVQLYA<br>LPWEDDPSSLDQYLRPGCLD
euGvar_LOC103599058p	XP_008581369	Leutx	yes	RT	181	MDTEGRLYCHTFKCQPVARTRDHQTCTTFRQEQLRKLREMFKKTTFPEKVTIQNLASVLHLNQLVVKTWFKNQHVKWKK*QQQTQP<br>GISNQTISVKEEKTALPITSVNTHPMSPEILIDHDHDLCDPSDIKQTGEAGASVCNSSLDSQPYDIQQICLEVSDPPWATIPYEID<br>LFVQLYVLP
laOros_LEUTX	XP_012416434	Leutx			186	MAENPRYARRGRTHFSVEQLQALKRVFEKTMYPDWATTEELISITHLDESVVKTWFKNQRVKRKKEQQPNGSNSSRESPNPTIPGK<br>EEGESLPVTSANSHLKSPSISDASDHELPQSPENDEPGPSRGNSSWDSQPSDLQERCLGDSDPPWASSPYDIDQFIQLYALPGDDD<br>PSSLDQYLPPKCRS
laLwed_LEUTX	XP_006743481	Leutx	yes		186	MAENSRYARRGRTHFSVEQLQALKRVFEETMYPDWATTEELISVTHLDESVIKTWFKNQRVKRKKEQQPNGSNSSRESPNPTVPGK<br>EEEESLPVTSANTHRKSPSISDASDHELAQFSENDEPDPSRGNSSWDSQPSDLQEICLGDSDPPWASSPYDIDQFIQLYALPGDDD<br>PSSLDQYLSPKCCS
laClup_AOCS01144808	AOCS01144808	Leutx	yes		195	MAENQRCARRGRTHFKKEQVQALKRVFEETMYPDWVTMEELISITHLDESVIKTWFKNQRVKRKKEQQQTGSNSSLESPNPNSSLE<br>SSNPTISGQEEEPSLTVTSANTHPRSPNFLDACDHELPQSSDNGQPGPSRWGSSWYSLPPDLQQICLGDSDPLWASSPYDIDQFME<br>LYALPGDDDPRSLDQYLSPTCLG
laFcat_LEUTX	XP_006941408	Leutx	yes		201	MAEEQRYARRRRTHFSAEQLQALKQVFEETMYPDWVTMMELISSTQLDESVIKTWFKNQRVKRKKEQQQTDSNSSLKSPNQTISIK<br>EEQPPLPVTSANTHPGSPSILDACDHELPQSPDSVQSAGAGPSKWNSSWDSPPYDLQQICLGDPDPPWASIPYDIDQLIQLYALPG<br>DDDLDQYLSPRCPSSGDMADIPTLIEKDL
laBtau_LEUTX	XP_010821986	Leutx	yes		208	MAEKQNSTRRFRTRFNGEQLGALRDVFERTRYPHCFLIRTLASTIHLDASVIKTWFKNQRVKRRREENQTQQNLSPGDPRRVVSVK<br>EEEMPLPGTSGSTHPTSLSLAGDSHHELPELSCAEQCEGAAATPCPSSCNFLTALSLRDTDLPWASSPYDMDQLIQLYDLPGDDDP<br>SSLDQYLFPECSSWGTVAGTDHHREDEHSYNPEKVP
laBmut_M91_07100	ELR60147	Leutx	yes		208	MAEKQNSTRRFRTRFNGEQLGALRDVFERTRYPHCFLIRTLASTIHLDASVIKTWFKNQRVKRRREENQTQQNLSPGDPRRVVSVK<br>EEEMPLPGTSGSAHPTSLSLAGDSHHELPELSCAEQCEGAAATPCPSSCNFLTALSLRDADLPWASSPYDMDQLIQLYDLPGDDDP<br>SSLDQYLFPECSSWGTVAGTDHHREDEHSYNPEKVP
laBbub_LEUTX	XP_006050146	Leutx	yes		208	MAEKRNSTQRFRTRFNGEQLGALRDVFERTRYPHWFLIRTLASTIHLDASVIKTWFKNQRVKRRREENQTRQNLSPGDPRQVVSVK<br>EEEMPLPGTSGSIHPTSLSLAGDSHHELPKLSCAEQCEGAAATPCPSSCNFLTALSLRDADPPWASSPYDMDQLIQLYHLPGDDDP<br>SSLDQYLFPECSSWGTVAGTDHHRDGEHSYNPEKVP
laChir_LEUTX	XP_013826545	Leutx	yes		212	MAEKRNSARRFRTRFNGEQLGALRDVFERTRYPHWFLIRTLASTTHLDESVIKTWFKNQRVKRRREENQTQQNLSPGDPCQVVSVE<br>EEEMSLPGTSGSTHPTSLSLADDSHHELPELSCAQQCEGAAATPCPSSCNFLTVHLHQISLGDSDPPWASSPYDMDQLIQLYDLPG<br>DDDPSSLDQYLFPECSSWGTVAGTDHHRDGEHSFNPEKVP
laPhod_LEUTX	XP_005963589	Leutx	yes		212	MAEKRNSAHRFRTRFNGEQLGTLRAVFERTRYPHWLLIRTLASTTHLEESVIKTWFKNQRVKRRREENQTRQNLSPGDPCQVVSVE<br>EEEMPLPGTSGSTHPTSLSLADDSHHELPELSRAQQCEGAAATPCPSSCNFLTVHLHQISLGDSDPPWASSPYDMDQLIQLYDLPG<br>EDDPSSLDQYLFPECSSWGTVAGTDHHRDGEHSFNPEKVP
laCfer_LEUTX	XP_006178018	Leutx	yes		188	MAEKPSTTRRFRTHFSRGQLRVLRDTFEKTRYPNWFIVNQLSSSIHLDESIIKTWFKNQRVKWRKQEQQTGENLSLEDLQQVSSVE<br>EEEASSPATSANSRPTGLSISDASDHEPPKSGIEQPQAAGATPCSSSCGLLPDDLQQINFRDFDLPWASTPYDMDQFIQLYALPGG<br>DGPWSLDQDLFPECSD
laVpac_LEUTX	XP_006215158	Leutx	yes		188	MAEKPSTTRRFRTHFSRGQLRVLRDTFEKTRYPNWFIVNQLSSSIHLDESIIKTWFKNQRVKWRKREQQTGENLSLEDPQQVSSVE<br>EEEASSPATSANSRPTDLSISDASDHEPPKPGIEQPQAAGATPCSSSCGLLPDDLQQINFRDFDLPWASTPYDLDQFIQLYALPGG<br>DGPWSLDHDLFPECSD
laSscr_LEUTX	XP_013844021	Leutx			195	MQEKPTSSRRSRTYFSREQLRVLTDTFEKTRYPNWFIVNTLSSNIHLDESVIKTWFKNQRVKRRKKERETQQNPPLEDTPQVSPVK<br>EEETLSPSASGNTRSMSLSISDASDPDSPQPSCAETCEGAAPCEGAAATPCDSSCDFLPEDLRQISFRDPDPPWASSPYDMDQLIQ<br>LYNLPGEDDPSSLDQYLLPLCST
laEcab_LEUTXp	XP_005614259	Leutx	yes	RT	180	MSTKSRCTWSHAIFILEQQTVLEDVFRKTMYPDWITLKEQVSILNFDDPVVKDWFKNHCMKWKKQEGKIKRWLLPIPPNQTVSVTE<br>EIPYPQLLRIFILSVLKFHVPRVLNHLRLLGIEQSGGASASFWNSSQDSQPYDAHQTHLGVFGTPEGLRSLL*QRPICTIIYLT*D<br>DDLSSLD*
laEcab_AAWR02030248	AAWR02030248	Leutx	yes		189	MMAEKPRSARRCRTVFTPEQLRALKDVFEKTMYPDWFTITELTSSIDLEESVIKTWFKNQRVKRKKQQQQIRPSPSPGGPNQTTSA<br>KEEEAPLPVTSAATHLTSPAVSDDCGHEPPKPSGIEQPGGAGASVWNSWDPQLQNLQQICLGVSDPPWASVPTDMDEFIQLYALPG<br>DDDPSSLDEYLFPKGPS
laEprz_LEUTXp	XP_008509435	Leutx		RT	178	MSTKSRCTWSHAIFILEQQRVLEDVFRKTMYPDWITL*EQVSILNFDDPVVKDWFKNHCMKWKKQE*KIKRWLLPIPPNQTVSVKE<br>EDSLPTAAANIHPVSSGISCAQGPESPEVSGIEQSGGASASFWNSSQDSQPYDAHQTHLVVFGTPEALVPCNIDQFAQLFTLPXDD<br>DLSSLD
laEasi_JREZ01000468	JREZ01000468	Leutx	yes		189	MMAEKPRSARRCRTVFTPEQLRALKDVFEKTMYPDWFTVTELTSSIDLEESVIKTWFKNQRVKRKKQQQQIRPSPSPGGPNQTTSA<br>KEEEAPLPVTSAATHLTSPAVSDDCGHEPPKPSGIEQPGGAGASVWNSWDPQLQDLQQICLGVSDPPWASVPTDMDEFIQLYALPG<br>DDDPSSLDEYLFPKGPS
laCsim_LOC101387510	XP_004441658	Leutx	yes		188	MAETPRSARRCRTAFTPEQLRALKDVFEETMYPDWATIMELTSMIHLEESVIKTWFKNQRVKRRKQQQQTRPSPSPEAPNQTTSVK<br>EQETLLPVTSANTDPTSPSISDDCEHEPPKPSGIQQPGGAGASVWNSWDSQSHDLQQICLGLSDPPWASVPYDMDEFIRLYTLPGD<br>DDPSSLDWYLFPKCPN
laPale_PAL_GLEAN10001506	ELK18207	Leutx	yes		186	MAKKPRSARRHRTTFSPKQLRILKETFEKTMYPHWFIITALTTVIHLDESVIKTWFKNQRIKRRKQQQLQTRPLLPLEAPNQTISV<br>KEEETPSPVASTDTHPVSEARDRDPPKAPGIEQPGGAGASAWNASWDSQPHDLQEMCLEASDIPWATMPYDIDQFIRFYALPGDDH<br>PNSLDQYLFPKQPG

Gene-Locus-name	Accession Nr	Gene family	Manual changes	RT	Seq. length	Sequence
laMluc_LEUTXp	XP_006086093	Leutx	yes	RT	186	MAERPRSAHWYCTTVSPEQ*LWKDTFEKTMYPY*VTMAELASATQLDELVKNNWFKNQHIKRRKQQLQTQPSLALEAPNQIITVNE<br>ETSLSVTSTNACPISPSVVNAYDHEPSKTSGFKQPGGAGCLCVECIMGFSASGSPLCLGVSDLPLASIPYDLDQFTHLYALPGDDH<br>PNSLDQCLFPKCPG
laMbra_LEUTXp	XP_005878172	Leutx	yes	RT	89	SAHWYCTTFSPEQLRILTDACGKTMYPHWDTMAVLTSATQLDESVIKSWFKIQCIKRRKWLLQCQPSLALEAPNQTIPTFVKEEEA<br>PLP
laMjav_LEUTX	XP_017529204	Leutx	yes		202	MEEKPRYARRSRTNFTLEQVRALKDIFEKTMYPSWITMNELISVTRLDESVIKTWFKNQRVKRRKQQRPTQPSPSLEAPYKTASVK<br>EEDSPSAEMSANTSPTSLSISDHCDRESPEPSGIEQPRRVGASGGNSSGDPQPSDLQQICLGVSDAPWASIPYDIDQFIQAYALPV<br>DDDPCGLDQYLLPKPPGWRGRAGTDLQCDG
afLafr_LEUTX	XP_010598698	Leutx	yes		190	MSAKPRLGHHCRRTIFTPQQRTALEDVFRTTMYPDWRTIETLTSTLNLDESVVKSWFKNHRVKWRKEQQQAQSRQAPEEPNHVAPV<br>KKEEMPLPITDANPGPVSARSLDASEPDPPEPPGAGQPGGAGACVWNSSWDSHTHDAQEINLDILDPPWATIPCDIDEFVELYALP<br>GDDDPSSLDQYLLPRCPC
afLafr_LEUTX2	XP_010598698	Leutx	yes		201	MSAKPKLGCHRRRTIFTPQQRTALEDVFRTTMYPDWRTIETLTSTLNLDESVVKSWFNNHRVKWRKEQQQAQSRQAPEEPNQVAPM<br>KKEEIPLPITDANPGPVSARSLDASEPDPPEPPGAGQPGGAGACVWNSSWDSHTHDAQEINLDILDPPWATIPCDIDEFVELYALP<br>GDDDPSSLDQYLFPKAHLLRESVQTINSS
afEedw_LOC102857301p	XP_006893173	Leutx	yes	RT	173	HDCSVRRKRTLFTDQSL*TMEYEFRKTMHPGANTRERLASTLGLDQNVVNIWFKNRRVKWRKEEREAQCQLSQRGPSEEAPGTGQE<br>TLLPINPGETSRMRAGHLYAPDPGQPGEALGFVDNSSGHQQVHGVHPITLGTTIPPWADCPYHIEAFVELYALPGDDDPSCMDQYL<br>N
xeDnov_LEUTX	XP_012376263	Leutx	yes		206	MSGKPPKYSRRCRTHFTTRQYTELEAMFSETKYPSYNAMEELASALHLDQYVVKTWFKNRRIRWKKQQGQTQPKVSQEAPTKTVSV<br>KEEETPLAITDAQSPPSPGISDPRNPETPEGSGSEHPKGAGASAENSSWNSRPCDLQDKCPPWASILCDVERFVELYDLPGEVDPS<br>ELDQYLFPERLRAGGCQHRSSGGLLTSTAPENDL
roCcan_Leutx	XP_020010762	Leutx			173	MSERPHHARRQRTRFSEGQLRVLRDVFNETKRPDWDTVQALAARLQLDVAVIKTWFKNNLVRWKKQQQEKEPNPKPGGNAPDTFSV<br>SSRGLDDAKQEPQGSSGDKGPGVTGDPALSSQCALEPCDIHEIHLEDVVPPWASMPYNMDQLVELYALPGDDDPDSLDRYLLPGCL<br>H
roItri_Leutx	XP_005336543	Leutx			181	MAGNFQHVRRHRTTFTAEQLEVLKSVFQKTQIPSFETVAELASTLDLDEAVVKTWFKNQRAKWKKQQQEMQPEVLPGPSTQNTAEN<br>EVVSPAPVNSKNGVPCDSATTHHQDDGVPSSSGLGQPVLDSERDCLPSDLKDICLRVSDSPWANTTLDMDQFIKMYHISGEEDPRS<br>LDRYLLPLA
roHgla_Leutx	XP_004875632	Leutx	yes		187	KEQYTRRPRTVFSQNQLAVLMDTFEKNTHPDTDAIHELASKLNLDEVVIKNWFKNQRMKQKKIRLKVQGDASPGTSTQQVLEEKEP<br>PLQNPATNASPIAGSSDDGNHDPQEPSESHITRRDGAPVLSSAVDTPHDNVQDTCMGDLDVPWAHTPGDISEFVELYALHEEDDPS<br>SFDVYLPTRVPSVRE
roFdam_Leutx	XP_010612841	Leutx	yes		184	MGEKEQYTRRPRTVFSQNQLAVLMDAFEKDTHPDTDAIHELQSKLNLEEAVIKIWFKNQRMKQKKIRLRVQQEASPGTSTQHVLEK<br>EEAALPNPKSNASPIAGTSEDGNHEPSESHSTRRHEAPAWRPAVDSQCDDIQHICLQDWDVPWAGTPGDINQFIELYALPEEDDPS<br>SLDIYLSPRCLQ
roDord_Leutx	XP_012891549	Leutx	yes		209	MADKAPAPRRRRTKFTEHQLSELRKFFSITQHPRWENIQALALQLQLDQSVVKAWFKNQVSKLKKQQQQQEEGRPVNPEAPQDQA<br>NNKMQVPWACALSPQQPSRIWVPQGPEQAQQAAASSFPPASQSVDDLEVSWTDPLPYSTEELADIYDVSGDEDPSSLDEYLPGEPA<br>LLGTNNPNVSPDSNISQAWERWTLHTHCWPSVPTKSK
roDord_LOC105990859p	XP_012878829	Leutx		RT	192	MAEQLRAHRKSRTRFTQHQLQELRAAFKHTQHPKWDEVQELASRLQLDEIEVKVWFKNQKAKLKKQQQAGKPPVCPSASCCQAAHG<br>VQVPGAALLSPQQPFRGEPHQGPEELQEFGAPTSPGLSFPLAAQPLESLEVSWTAPLPYSTEELVRVYGVSGYEDPSSLDKYLLPE<br>PELPGAHSSQPQDNISMLQP
roDord_LOC105990857p	XP_012878828	Leutx		RT	192	MAEQLRAHRKSRTRFTQHQLQELRAAFKHTQHPKWDEVQELASRLQLDEIEVKVWFKNQKAKLKKQQQAGKPPVCPSASCRQAAHG<br>AQVPGAALLSPQQPFRGEAHQGPEEPQEFPAPTSAGLSFPWAVQPLESLEVSWTAPLPYSTEELARVYGVSGDEDPSSLDKYLLGK<br>PALPDQHSPKPQDNISMLQL
roClan_LOC102027798	XP_005414154	Leutx			189	MSQVPDDLKYNRRPRTRFTHSQLSVLMDAFQNNNLPSLAVVKELAATLELEEVTIKVWFKNQRAKHKKKQLKMQLDSSPGTSMKHV<br>LEKEETPLTKPATNTSPSSAASDGHNLHPQEPSDTQITGRDGASAFPSSPHIQCHDIQEESLENCGTPCTHDFFDTCQLLELYDVP<br>GDDLSGLDIYLPPGCFQ
roClan_LOC106150198	XP_013377737	Leutx			190	MPHVPDDLKYNRRPRTRFTQSQLSVLMDAFQNNNLPSLAVMKELAATLELDEVSIKVWFKNQRAKQKKKQLKMQLDSSPGTSMQHV<br>FKKEEAPWPKPATNPAPSSAASDGQNPHPQEPSDAQITGRDGASAFPSSSHTQCHEIQEESLENWDKPCTHDLLDTCQLLELYDMP<br>GEDDLSALNLYLLPECIE
roClan_LOC106147540	XP_013365100	Leutx			190	MPQVPDDLKYNRRPRTRFTHSQLSVMMDAFQNNNLPNPAVVKELAATLELDEVSIKVWFKNQRAKQKKKQLKMQLDSSPGTSTRHV<br>FEKEEAPLPKTAANPAPSSTASDGQNPHPQEPSDTQITERDGASAFPSSSHPQCYDIQEESLENWDKPCTHDLLDTCELLELYDMP<br>GEDDLRALNLYLVPECFQ
roClan_LOC102027472	XP_005414153	Leutx	yes		190	MPQVPDDLKYNRRPRTTFTQSQLSALMDAFQNNNLPSLAVVKELAATLELEEVTIKVWFKNQRAKQKKKQLKMQLDSSPGTSTQHV<br>LEKEEAPLTKPATNPAPSSAASDGHNPHPQEPSDTQITGRDGASAFPSSPHIQCHDIQEESLENCGTPCTHDFFDTCQLLELYDVP<br>GEDDLSGLDIYLPPGCFQ
roCpor_LOC101788084	XP_005002641	Leutx			186	MSSKPDKIEYRRKPRTDFTPSQRSVLLSAFEKNNHPGHDVTKVLATKINVDETVIKIWFKNQRSKWKKDQLKLKLASLPGTSMQTS<br>SEKKEQVLQSPVASCSPISSISDDHVCDRAERFEKDISRRHRASVFGSSFQSQPDGTQGHVVYVEDFDIDDTDKLIEMYYLPGEDD<br>PSSLDIYLPPGCLQ
roCpor_LOC101788138	XP_005002643	Leutx			186	MSRKPDKIEYRHKPRTDFTPSQRSVLLSAFEKNNHPGHDVTKELATKINVDETVIKIWFKNQRSKRKKDQLKTKLASLPGTSTQTS<br>SEKKEHVLQSPVASCSPISSISDDHVCDRAERFEKDISRRHGASVFGSSFQSQPDGTQGHVVYVEDFDIDDTDKLIEMYYLPGEDD<br>PSSLDIYLPPGCLQ
roCpor_LOC101788113	XP_005002642	Leutx			184	MSSKPDKIEYRRKPRTDFTPSQRSVLLSAFEKNNHPGHDVTKVLATKINVDETVIKIWFKNQRSKRKKDQLKLKLASLPGTSTQTS<br>SEKKEHVLQSPVASCSPISSISDDHVCDRAERFEKDISRRHGASVFGSSFQSQPDGPQGQYVEDFDIDDTDKLIEMYYLPGEDDPS<br>SLDIYLPPGCLQ
roCpor_LOC101788050	XP_005002640	Leutx			184	MSSKPDKIEYRRKPRTDFTPSQRSVLLSAFEKNNHPGPDVTKELATKIKVDETVIKIWFKNQHSKRKKDELKTKLASLPGTSTQTS<br>SEKKEQVLQSPVASCSPISSISDDHVYDRAERFEKDISRRHGAFVFDSSFQTQPVEIQGQYVEDFDIDDTDKLVEMYYLPGEDDPS<br>SLDIYFPPGCLP
roCpor_LOC101787990	XP_005002638	Leutx	yes		182	MSGNPENIQYTHKMRTEFTPSQHLVLLSAFEKNKHPGHDVTKELAAKLNFSESVIKHWFKNQRMEWKKNQLKVKLASSPGTSTQPS<br>LEQEAPQNPVTSPSPISVTSNDHIHDPLELLESEITRREGTSVFGSSFDTQCDNIQGQYVEDFANLDTDKLVELYYLPGEDDPSSL<br>DIYLPPGCLQ
roCpor_LOC101788169	XP_005002644	Leutx			186	MSRKPDKIEYRRKPRTDFTPSQRSVLLSAFEKNNHPGHDVTKELATKINVDETVIKIWFKNQRSKQKKDQLKLKLASLPGTSTQTS<br>SEKKEHVLQSPVASCSPISSISDDHVCDRAERFEKDISRRHGASVFGSSFQSQPDGTQGHVVYVEDFDIDDTDKLIEMYYLPGEDD<br>PSSLDIYLPPGCLQ
roCpor_LOC101788198	XP_005002645	Leutx			186	MSSKPDKIEYRRKPRTDFTPSQRSVLLSAFEKNNHPGHDVTKELATKINVDETVIKIWFKNQRSKQKKDQLKLKLASLPGTSTQTS<br>SEKKEHVLQSPVASCSPISSISDDHVCDRAERFEKDISRRHGASVFGSSFQSQPDGTQGHVVYVEDFDIDDTDKLIEMYYLPGEDD<br>PSSLDIYLPPGCLQ
roCpor_LOC101788018p	XP_005002639	Leutx	yes		178	KIEYRRKPRTDFTPSQRSVLLSAFENNNHPGHDVTKELATKINVDETVIKIWFKKQCSKRKKDQLKMKLASLPGTSTQTSSEKKEQ<br>VLQSPVAICSSISSISDDHVCDRAERFEKDISRRHGASVFGSSFQSQPDGTQGQYVEDFDIDDTDKLIEMYYLPGEDDPSSLDIYL<br>PPGCLQ
roMaur_Leutx	XP_005086314	Leutx	yes		254	MSGQPLHDHQYCTIFSLEQIKVLQEAFNNNMCPSKEGIQELASRLQLKDVVVKMWFRNQRDKLQIQQQNHQWSSSLAASNQVKVVR<br>RASSKQATREMGASSSQAKXXXXVSSNQAPREMGASSSQSKRVRKASSNQAKKVRRASSKQVKSLKDDGILAPKADYAQLLSTGIS<br>DNSDQESWKNFGTMQPEESGASAGKYVEHSELLDIYQSSLGLAQPPWASMPFDIDTFVRIYDLPGDNNPQEFDKYLYPGCLD
roCgri_I79_012385	EGW06396	Leutx	yes		1046	MSGQTRHDLPYSTIFSPEQIGVLQEAFNSNMFPSKKESQELASRLQLKDTVVKMWFRNQRDKLQTQQQNHHGSSSLVESRWVKVVK<br>GVSSNQASREMGASSHQAKRLRKESSNQGSREMDASSNQGQRRMGPSSNEGPDGMIALCNQSPREMAASSNQPPREMDESSNQAKR<br>VRKASSNHIPREKGPPSNLALREMDASSNQPPRQMGASSNQPPREMDASSNQPPREMDASSNQAKRVRKASSNQAPREMDAPSNQN<br>KRVKKASSNQRPREMGPSSNQPPRQTGASSNQALREMGPSSNQPPREMDASSIQAKRVRKASSNHMPREKGPPTNQALTEMGVSSN<br>QLPREMIALFNQAPREMAASSKQGTREMDALFNHALREIGASSNHAKRQRNAPSNQPPRQMGASSNQPPREMDASSNQAKRVRKAS<br>SNHAPREKIPPSNQAPREIAASSNQGPREMDVLFNQALREMGASSNHAKRLRNETPSQPLRNMDALSNQPPRQMGASSNQPPREMD<br>ASSXXXREMDASSNQKPRVIATSSNQAKSEMGALSKQASREMWASPNQAKTVRKVSSNQAPREMGPSFNKAPREIRPKVSSSQGPR<br>ELCPSSNQPLSKIDASSSQALKDMEALLDQALRERGASSNQPSREIDALSKLASKEMDASTNPKPTEMGASSNQPAREMDALSKQA<br>SREMCASTNQPPREMCASPNQPPSEIDASSSQALREMEALLDQALKEIGETSNQASREMDASSNKAPREVHASSNQASRETDASSY<br>QVPREMAVSTSHALAELDELVNQLLREMNASSNQASREMDTSSNQAKEVRKLSSKQAKQVRSLSSTQDKRVKKASSNLAKIDRDAF<br>SSPAPREIGAPSNQAPRKKGASSNQGKTVRKQSSNDAKGVKRVSSKQAMKERDASSNQTPREMGAPSNQDKTVRGVASMQEKSLKD<br>NEVFAPKADSAQLLSTRGSDSNQESWKKIGAKQPEESGASVRKYVEHSELLDIYQSNLGPVQPPWASMPFDIDTLVNMYELPGDND<br>PQEFDKYLYPGCLD
roCgri_I79_024957	EGW10548	Leutx	yes		659	MSGQKFQELPYSTIFSPEQIGVLQEAFNSNMFPSKKESQELASRLQLKDTVVKMWFRNQRDKLQTQQQNHHGNSSLAESSRVKAVK<br>RVSSNQAYREMGASSQQAKRLRKASSNQVPRNMKTLSNQSSRQMHISANQLPRQMDALFKQASREMGASSNQGPRVMGPSFNQAPS<br>EMCPSSNQVPRDMDAVFNQEPREMGASSNQGPRVMGPSFNQAPSEMCPSSNQVPRDMDAVFNQEPREMGSSSNQPQRKIDAFFNQA<br>SKGMGASSNQGPREIYALSDKASREMGTSSNQAKRVRKASSNQAHREMGPSSNQAVSEMDALCRQALREMEALLNQTLREIGESSN<br>QASREMDSSSNQAPREVDASSNPAPREMDALSNQVPREMGVSSSQALTELDALLNQLLREMNESSNQSSREMGSLSNQDSREMGPS<br>ETSREIGALSNQAPREMDVSSNQALREMGSSSNKASSEMVAQSNQEPREKGALCNEASRDMCTLSNQALRTMDASSNQALREMDAS<br>WIQELRDMSVLLKDASKEMDASINQAETVRGAPSKQMISLKDDEVFAPKADYAQLLNTNGSDNSDQESWKNFGAKQPEESGASVGK<br>CMEYSELLDIYQSSLGLVQPPSESMPFDIDTLVKIYNLPGFDEPLVFDKYFYPGCID
roNlep_A6R68_07039	OBS64421	Leutx	yes		583	MSGQLQHDHQYCTIFTMDQIRALQDAFHSNMFPTKEEIQELASRLQLKEIVVKMWFKNQRDNLQTQQGNDQWSSSLVASGQIKLVR<br>RASSNQAMRQMGASSNQAMREMAASSNQAMREMGASSNQVMVGLASSYSQATIEMAASSNQAKRVRRASSNKAKRVRGASSNKAKR<br>VRGASSNKAKRVRGAPSKQAMREMTASSNQRTIEMGASPNHAVREMGASSNQAITELAASSNLATLEMAVSSNQAMREMGASSNQV<br>MVKLASSSSQATIEVAASSNKAKRVRGAPSKQAMREMTTSSNQRTIEMGASSNQAKKVRRALSNKAKRVRGAPSKQAMREMTASSN<br>QAIIELAASSNQVKKVRRASSNQRTIEMGASSNQETLEMDASSNQGTTEMGASSNQETLEMDASPNQGTIEMGASSNQETIEMDAL<br>SNQGTIEMDASSNQETLEMDASSNQATRDMGASSNQAKPIRGAVCKQVNFLKDDELLAPKTDYAQLLSTRGSDNSGQESQNFGPKQ<br>PEEAGASTVMSLEHSELLELYQTCLGLVQPPWASLPFDIDTLVQMYALPGDDDPESFDKYFCPGFFD
roPman_AYHN01138932p	AYHN01138932	Leutx	yes		361	RRAASNQAKTVRSTSSSQAMREMCASSNQAMREMNVTSKQVMIKLPSSSSQAPMEMAVSSNQRTTEMGESSNQAKTVRRAASNQVN<br>RVRRAASKQTIIE*AASSNQATLEVAASSNQAKRVRGASSKQSKRVGGASSNQETMEMAATSNQAKRVRGASSKHSKRVRGASSNQ<br>AKRVRGASSNQETMEMAATSNQGTMELAATSIQAKPVRGVPCKQVKSLKDGEXXXXXXXQGTMELAATSIQAKPVRGVPCKQVKSL<br>KDGEILAPKTDYAQLLSTRGSDNSRQESQKNFGPKQPEEVDISAVKSLEHSDLLEIYQTCLGLVQPPWASKDFDIDTLVKIYSLPG<br>DDDPKSFDKYLYPGLPD
roPman_Leutxp	XP_006998816	Leutx	yes		143	SKQGTMEMAASSNQGTMELAATSIQAKPVRGAPCKQVKSLKDGEILAPKTDYDQLLSTRGSDNSRQESQKNFGPKQPKEAGISAVK<br>SLEHSELLEIYQTCLGPVQPPWASKDFDTDTLVKMYALPGDDDPKSFDKYLYPGLPD

Gene-Locus-name	Accession Nr	Gene family	Manual changes	RT	Seq. length	Sequence
lagOpri_AAYZ01197513	AAYZ01197513	Leutx	yes		433	SGNAARRPRTGFKPQQLGVLKAAFEKTPCPDLDTLESLAMELNLEMSVVKTWFKNKRGNLSRHQQKQKQEGLPKRLAPVAWDTSSH<br>AMQEKSLSDSNLNPPKIVPFHLQPLPPQPQSPSQAPTATSPQAPPASGHNSQHGSRPSHLDHLVLDPSAPWAFLPEHSNMQSLLPQ<br>PQPHYQLPRYTPLDAPPASGNTSQQGSCPIQQELLCLDPLAPWASLPAHSHLQPLPEPPPQPHYQALTSTSQAPPASGHTSQHGST<br>PTHLEHLCLEAADSWASLSAHSHLQPLPPQPQPQPHYQAPTSTPLNAPPASGNTSQQGSWPSHLAHLCLDPLVPWASLPAHSHLQP<br>LPPQPQPQPPSQAPTSIPPQAPLPSRHNSQLGAWPSDLENLVLDPSPPWASLPDDLDQLVRMYGAPLDVDPNSLNVYLLHPDQLRH<br>CAT
lagOcun_LEUTX	XP_017197675	Leutx	yes		156	MSSEQVAEAAPRCARRSRTAFTPDQLGMLKAVFETTPCPDWEKIQELAAKLCLDECVIKTWFKNQRAQQRKLQRKGQPRQPGESPP<br>PPGSELSTPSQEDHGPESSQAHGPEAAEDPLPADLQNIHLDSSAPWASLPHDIGELVRMYGLFDDDEPDD
lagOcun_LOC103348417	XP_017197882	Leutx			180	MSSEQVAEAVPRSSRRSRTAFTPDQLGMLQAVLETSRCPDWSRVQELAPELHLDVYVIKTWFKNQRAQQRKLQPKGQPRQPSESTP<br>QPPPPPPLPPPLLLPLPPPPPPPPPTCSELSAPAEEEQGPGGSQAQGPEAAEDPLPADLHNIHLDSSAPWASLPHDIGELVRMYRL<br>FHDDEPDD
lagOcun_LOC103348423	XP_008255388	Leutx	yes		146	PRCARRSRMAFTPDQLGMLKAVFETTPCPDWEKIQELAAKLCLDECVIKTWFKNQRAQQRKLQPKGQPRQPGESPPQPGSELSAPS<br>QEDHSPESSQAHGPEAAEDPLPADLQNIHLDSSAPWASLPHDIGELVRMYRLFDDDEPDD
lagOcun_LOC103348415	XP_008255352	Leutx	yes		176	PRSSRRSRTAFTPDQLGMLKAVLETSRCPDWSQKEELSSELHLDAYGIKTWFKNQRAKQRKLQPTGQPRQPSESTPQPPPPPPPPP<br>PPPPPPPPPPPPTGSELRAPAQEEQGPGGSQAQGPEAAEDPLPADLHNIHLDSSAPWASLPHDVGELVRMYGLFQDDEPDDLAEYL<br>YPQA
lagOcun_LOC103347612	XP_008252344	Leutx			113	TWFKNQRAKQRKLQRKGQPRQPSESTPQPPPPPPLPPPLLLPLPPPPPPPTCSELSAPAEEEQGPGGSQAQGPEAAEDPLPADLHN<br>IHLDSSAPWASLPHDIGELVRMYPLVP
lagOcun_AAGW02026615	AAGW02026615	Leutx	yes		204	MEGTQLLEPSLLTPWVSINKKVKIEADQGLEPKHSHMGCCTKHLPKRFYLKYIHCSTTLFTNNVSSTSVQTATLYSLIRTYHNLPR<br>PLFIKILSPFFFIIKKLCEISLNIPWYLNFCQSPISRVKIKDSHTSLPLPCYTEKLVPLGRISHRILCLVRFYGCLRAHTPPWVSI<br>FYKIDQLMQLYDFPREDDPSNLLKYVSPKCPS
roMmus_Leutxp	XP_006544220	Leutx	yes		827	SHASDGAWFNQATPVNGASMNQAMPVNRALMNQATPVSRAWFNQTTPVSRAWFNQATPVIGTSSNQSTPVHGASMNQATPVNGALM<br>NQTTPVSRAWFNQATPVIGTSSNQSTPVHGASMNQATPVNRASMNQAMPVNGALMNQATPVNGALMNQATPVNGALMNQAMPGIGT<br>SSNQAMPGSEAWFNQAMPVREPLSNQATSVRRTLFNQAMPEIGTSSNQAMPVSEASINQAMPVSEASINQAMPVSGASINQAMPVS<br>EASSNQAMPVSGALINQAMPVSKASISQAMPVSGALINQAMPVSKASISQAMPVSGILINQAMPVSQASVNQVKLKRRASSNQAMP<br>VSKALINQAMPVSEASINQAMPVSEASINQAMPVSGALINQAMPVSEALINQAMPVSEASSNQAMPVSGALINQAMPVSEASINQA<br>MPVSEASSNQAMPVSGASINQAMPVSEASSNQAMPVSKASINQAMPVSGASINQVMPVSGALINQAMPVSGILINQAMPVSQASTN<br>QVELKRRASSNQNPPVRKATFNQAKQRRRASSNQAMPLSKALINQAMPVCEASINQAMPVSEASINQAMPVSEASSNQAMPVSEAS<br>INQAMPVSGALINQAMPVSEALINQAMPVSEASSNQAMPVSGALINQAMPVSEASINQAMPVSGASSNQAMPVSGSSSNQAMPVSG<br>ASSNPAKQRGRASSSKAKSRRRALSNKAMPARGAPSKQTQSLKDDQIPTPTADCAHLLNPKSSDNSVQESQKNFGAKKPEEAGTSD<br>SQLLEVDQICLGHSPPPWASMPFDIDTLLEDYALSGDEDSQSFDKYLYPGCLD
roRnor_AAHX01004437.1p	AAHX01004437.1	Leutx	yes		276	GTSSREAWFNQAMPVSGALINEVMPARGALINQDTPMSGALINQATTVSGASINQAMAVSGALINQDTPRSRGLINQAMPVSGALI<br>NEAMPVRGVSVNQAMAMIRALFNQDTPMRGALIQAMSVRGALINQAMPVNKALINEVMPRRKALPNHALPVREASSKKAKPRRRAL<br>SNKARPARGAPHKQNKSWKDDQILDTSSDSSDQESQTNFGAEKPEGAGISEPQLLDSHQSCLGRSQPPWASVPCSIDTFVDIYALP<br>GDDDPQSFDNYLYPGCLD
prHsap_CRX	AAH16664	Crx			299	MMAYMNPGPHYSVNALALSGPSVDLMHQAVPYPSAPRKQRRERTTFTRSQLEELEALFAKTQYPDVYAREEVALKINLPESRVQVW<br>FKNRRAKCRQQRQQQKQQQQPPGGQAKARPAKRKAGTSPRPSTDVCPDPLGISDSYSPPLPGPSGSPTTAVATVSIWSPASESPLP<br>EAQRAGLVASGPSLTSAPYAMTYAPASAFCSSPSAYGSPSSYFSGLDPYLSPMVPQLGGPALSPLSGPSVGPSLAQSPTSLSGQSY<br>GAYSPVDSLEFKDPTGTWKFTYNPMDPLDYKDQSAWKFQIL
chLcha_CRX	XP_005987868	Crx			290	MMSYIKQPHYAVNGLTLTAPGMDLLHSAVGYPTTPRKQRRERTTFTRAQLDILEALFAKTRYPDIFMREEVALKINLPESRVQVWF<br>KNRRAKCRQQQQNNGQSKPRPTKKKSSPARETASETSTNGQYSPPPTGNSVTPSSTTSATVSIWSPASISPIPDPLSASTTPCMQ<br>RSTAYPMTYTQAPGYTQSYAGSTSYFSGLDCGSYLSPMHPQLSAPGSTLSPITNPTMGNHLSQSPASLSTQGYGTAGLGFSSVDCL<br>DYKDQTASWKLNFNATDCLDYKDQNSWKFQVL
chCnil_AAQ08476	AAQ08476	Crx			271	GLTLAGPGMDLLHSAVGYPTTPRKQRRERTTFTRAQLDILEALFAKTRYPDIFMREEVALKINLPESRVQVWFKNRRAKCRQQQQ<br>STGQAKARPAKKKSSPAREPSAEPSAAGPYSPAGPGPAGTPSSAPGATVSIWSPASISPIPDPLASSAAPCLPRSAPYAMTYSQAP<br>GYGQSYAGSGSYFSGLDCSSYLSPMHPPLGAPGAALSPIAAPSMGNHLSQSPAALSSQGYGAAGLGFGPVDCLDYKDQTGSWKLNF<br>NAADCLDYKDQSS

Table S1. List of sequences retrieved from Genbank. A unique genelocus identifier, composed of species code with clade prefix (see Table S2) and locus information, identifies each sequence. Predicted pseudogenes have a "p" postfix. Accession numbers refer to the protein sequence retrieved, unless the protein ORF was predicted de novo from genomic sequence, in which case the accession number for the genomic sequence is given. The "Manual changes" column indicates which sequences needed manual correction/annotation. In the case of Loxodonta africana, the same accession number is present twice, due to the fact that the two LEUTX genes (named Leutx and Leutx2 here) were merged inappropriately into a single ORF, with omission of several critical coding exons. The RT column indicates that the predicted ORF did not have introns and is most likely the result of reverse transcription. Several pseudogenes are not shown in this list; we did not conduct exhaustive searches for pseudogenes. Not all sequences are complete, in some cases contigs did not extend far enough to identify adjacent exons, or there were sequence gaps within contigs. The three CRX sequences were used as outgroup.

ID	Scientific name	Common name	Clade	Gestation period (days)
**Anan**	Aotus nancymaae	Ma's night monkey	eu, pr	133
**Bbub**	Bubalus bubalis	water buffalo	la	300-340
**Bmut**	Bos mutus	wild yak	la	
**Btau**	Bos taurus	cattle	la	286
**Cang**	Colobus angolensis palliatus	Peters's Angola colobus	eu, pr	147-178
**Caty**	Cercocebus atys	sooty mangabey	eu, pr	167
**Ccan**	Castor canadensis	American beaver	eu, ro	128
**Cfer**	Camelus ferus	Wild Bactrian camel	la	390
**Cgri**	Cricetulus griseus	Chinese hamster	eu, ro	20
**Chir**	Capra hircus	goat	la	150
**Cjac**	Callithrix jacchus	common marmoset	eu, pr	152
**Clan**	Chinchilla lanigera	long-tailed chinchilla	eu, ro	110
**Clup**	Canis lupus familiaris	dog	la	61
**Cnil**	Crocodylus niloticus	Nile crocodile		
**Cpor**	Cavia porcellus	guinea pig	eu, ro	65
**Csab**	Chlorocebus sabaeus	green monkey	eu, pr	163-165
**Csim**	Ceratotherium simum simum	southern white rhinoceros	la	~500
**Dnov**	Dasypus novemcinctus	nine-banded armadillo	xe	122
**Dord**	Dipodomys ordii	Ord's kangaroo rat	eu, ro	28-32
**Easi**	Equus asinus	donkey	la	365
**Ecab**	Equus caballus	horse	la	336
**Eedw**	Elephantulus edwardii	Cape elephant shrew	af	45-60
**Eprz**	Equus przewalskii	Przewalski's horse	la	
**Fcat**	Felis catus	cat	la	64
**Fdam**	Fukomys damarensis	Damara mole-rat	eu, ro	78-92
**Ggor**	Gorilla gorilla gorilla	lowland gorilla	eu, pr	257
**Gvar**	Galeopterus variegatus	Sunda flying lemur	eu	60
**Hgla**	Heterocephalus glaber	naked mole-rat	eu, ro	70
**Hsap**	Homo sapiens	human	eu, pr	270
**Itri**	Ictidomys tridecemlineatus	thirteen-lined ground squirrel	eu, ro	28
**Lafr**	Loxodonta africana	African savanna elephant	af	~670
**Lcha**	Latimeria chalumnae	coelacanth		
**Lwed**	Leptonychotes weddellii	Weddell seal	la	~300
**Maur**	Mesocricetus auratus	golden hamster	eu, ro	16
**Mbra**	Myotis brandtii	Brandt's bat	la	~50
**Mfas**	Macaca fascicularis	crab-eating macaque, long-tailed macaque	eu, pr	165
**Mjav**	Manis javanica	Malayan pangolin	la	~130
**Mleu**	Mandrillus leucophaeus	drill	eu, pr	180
**Mluc**	Myotis lucifugus	little brown bat	la	50-60
**Mmul**	Macaca mulatta	Rhesus monkey	eu, pr	164
**Mmur**	Microcebus murinus	gray mouse lemur	eu, pr	60

**Mmus**	Mus musculus	house mouse	eu, ro	19
**Nlep**	Neotoma lepida	desert woodrat	eu, ro	33
**Nleu**	Nomascus leucogenys	northern white-cheeked gibbon	eu, pr	210
**Ocun**	Oryctolagus cuniculus	European rabbit	eu, lag	31
**Opri**	Ochotona princeps	American pika	eu, lag	30
**Oros**	Odobenus rosmarus divergens	Pacific walrus	la	456
**Pabe**	Pongo abelii	orangutan	eu, pr	~250
**Pale**	Pteropus alecto	black flying fox	la	189
**Panu**	Papio anubis	baboon	eu, pr	187
**Phod**	Pantholops hodgsonii	chiru, Tibetan antelope	la	
**Pman**	Peromyscus maniculatus bairdii	prairie deer mouse	eu, ro	22-25
**Ppan**	Pan paniscus	bonobo (pygmy chimpanzee)	eu, pr	~240
**Rnor**	Rattus norvegicus	Norway rat	eu, ro	22
**Rrox**	Rhinopithecus roxellana	golden snub-nosed monkey	eu, pr	210
**Sbol**	Saimiri boliviensis boliviensis	Bolivian squirrel monkey	eu, pr	145
**Sscr**	Sus scrofa	pig	la	113
**Tchi**	Tupaia chinensis	Chinese tree shrew	eu	45-50
**Vpac**	Vicugna pacos	alpaca	la	345

Table S2. Species abbreviations and phylogenetic clade prefix abbreviations used in figures to generate the species codes. Prefixes are: pr: primates, ro: rodents, lag: Lagomorpha, eu: Euarchontoglires (unless ro, pr, or lag), la: Laurasiatheria (moles, carnivores, ungulates, whales, etc.), af: Afrotheria (aardvarks, elephants, sea cows, etc.), xe: Xenarthra (armadillos, ant eaters, sloths, etc.). Gestation periods are shown for selected species; information was retrieved from animaldiversity.org (Myers, P., Espinosa, R., Parr, C.S., Jones, T., Hammand, G.S., and Dewey, T.A. (2018). The Animal Diversity Web (online), University Museum of Zoology, University of Michigan) as well as a few additional web sources.

	prHsap_LEUTX	prPpan_LEUTX	prGgor_LEUTX	prPabe_LEUTX	prNleu_LEUTX	prMleu_LEUTX	prCaty_LEUTX	prPanu_LEUTX	prMfas_EGM_09724	prMmul_LOC699783	prCang_LEUTX	prCsab_LEUTX	prRrox_LEUTX	prAnan_LEUTX
prHsap_LEUTX	***	100.0	98.3	91.7	90.0	90.0	90.0	88.3	90.0	90.0	90.0	90.0	90.0	68.3
prPpan_LEUTX	0.0	***	98.3	91.7	90.0	90.0	90.0	88.3	90.0	90.0	90.0	90.0	90.0	68.3
prGgor_LEUTX	1.7	1.7	***	90.0	88.3	88.3	88.3	86.7	88.3	88.3	88.3	88.3	88.3	68.3
prPabe_LEUTX	8.9	8.9	10.8	***	88.3	83.3	83.3	81.7	83.3	83.3	83.3	83.3	83.3	66.7
prNleu_LEUTX	10.8	10.8	12.7	12.7	***	81.7	81.7	80.0	81.7	81.7	81.7	81.7	81.7	65.0
prMleu_LEUTX	10.8	10.8	12.7	18.9	21.1	***	100.0	98.3	100.0	100.0	98.3	98.3	98.3	65.0
prCaty_LEUTX	10.8	10.8	12.7	18.9	21.1	0.0	***	98.3	100.0	100.0	98.3	98.3	98.3	65.0
prPanu_LEUTX	12.7	12.7	14.7	21.1	23.3	1.7	1.7	***	98.3	98.3	96.7	96.7	96.7	66.7
prMfas_EGM_09724	10.8	10.8	12.7	18.9	21.1	0.0	0.0	1.7	***	100.0	98.3	98.3	98.3	65.0
prMmul_LOC699783	10.8	10.8	12.7	18.9	21.1	0.0	0.0	1.7	0.0	***	98.3	98.3	98.3	65.0
prCang_LEUTX	10.8	10.8	12.7	18.9	21.1	1.7	1.7	3.4	1.7	1.7	***	98.3	96.7	65.0
prCsab_LEUTX	10.8	10.8	12.7	18.9	21.1	1.7	1.7	3.4	1.7	1.7	1.7	***	96.7	65.0
prRrox_LEUTX	10.8	10.8	12.7	18.9	21.1	1.7	1.7	3.4	1.7	1.7	3.4	3.4	***	65.0
prAnan_LEUTX	41.1	41.1	41.1	43.9	46.9	46.9	46.9	43.9	46.9	46.9	46.9	46.9	46.9	***
prCjac_LEUTX	33.0	33.0	33.0	41.1	43.9	38.3	38.3	35.6	38.3	38.3	38.3	38.3	38.3	14.7
prSbol_LEUTX	43.9	43.9	43.9	50.0	46.9	50.0	50.0	46.9	50.0	50.0	50.0	50.0	50.0	25.6
euTchi_LEUTX	56.6	56.6	60.0	63.7	71.4	53.2	53.2	50.0	53.2	53.2	56.6	56.6	53.2	63.7
euGvar_LEUTX	71.4	71.4	75.5	71.4	79.9	71.4	71.4	67.4	71.4	71.4	71.4	71.4	71.4	71.4
laOros_LEUTX	67.4	67.4	67.4	71.4	75.5	71.4	71.4	67.4	71.4	71.4	71.4	71.4	71.4	60.0
laLwed_LEUTX	75.5	75.5	75.5	79.9	84.4	79.9	79.9	75.5	79.9	79.9	79.9	79.9	79.9	60.0
laClup_AOCS01144808	79.9	79.9	79.9	84.4	89.2	75.5	75.5	71.4	75.5	75.5	75.5	75.5	75.5	63.7
laFcat_LEUTX	71.4	71.4	71.4	75.5	79.9	67.4	67.4	63.7	67.4	67.4	67.4	67.4	67.4	60.0
laBtau_LEUTX	79.9	79.9	75.5	79.9	79.9	75.5	75.5	79.9	75.5	75.5	79.9	79.9	75.5	71.4
laBmut_M91_07100	79.9	79.9	75.5	79.9	79.9	75.5	75.5	79.9	75.5	75.5	79.9	79.9	75.5	71.4
laBbub_LEUTX	84.4	84.4	79.9	84.4	84.4	79.9	79.9	84.4	79.9	79.9	84.4	84.4	79.9	75.5
laChir_LEUTX	79.9	79.9	79.9	79.9	79.9	75.5	75.5	79.9	75.5	75.5	79.9	79.9	75.5	71.4
laPhod_LEUTX	94.3	94.3	94.3	94.3	94.3	89.2	89.2	94.3	89.2	89.2	94.3	94.3	89.2	84.4
laCfer_LEUTX	89.2	89.2	84.4	79.9	89.2	94.3	94.3	89.2	94.3	94.3	94.3	94.3	94.3	75.5
laVpac_LEUTX	94.3	94.3	89.2	84.4	94.3	99.7	99.7	94.3	99.7	99.7	99.7	99.7	99.7	79.9
laSscr_LEUTX	99.7	99.7	94.3	99.7	99.7	105.4	105.4	99.7	105.4	105.4	105.4	105.4	105.4	79.9
laEcab_AAWR02030248	75.5	75.5	71.4	75.5	84.4	75.5	75.5	71.4	75.5	75.5	75.5	75.5	75.5	71.4
laEasi_JREZ01000468	75.5	75.5	71.4	75.5	84.4	75.5	75.5	71.4	75.5	75.5	75.5	75.5	75.5	71.4
laCsim_LOC101387510	79.9	79.9	75.5	79.9	84.4	84.4	84.4	79.9	84.4	84.4	84.4	79.9	84.4	63.7
laPale_PAL_GLEAN10001506	79.9	79.9	75.5	79.9	84.4	84.4	84.4	79.9	84.4	84.4	84.4	84.4	84.4	75.5
laMjav_LEUTX	71.4	71.4	71.4	75.5	75.5	75.5	75.5	71.4	75.5	75.5	75.5	75.5	75.5	67.4
afLafr_LEUTX	89.2	89.2	94.3	94.3	105.4	79.9	79.9	75.5	79.9	79.9	84.4	84.4	79.9	75.5
afLafr_LEUTX2	89.2	89.2	94.3	94.3	105.4	79.9	79.9	75.5	79.9	79.9	84.4	84.4	79.9	75.5
xeDnov_LEUTX	79.9	79.9	84.4	84.4	89.2	79.9	79.9	75.5	79.9	79.9	75.5	79.9	79.9	60.0
roItri_Leutx	63.7	63.7	67.4	67.4	67.4	56.6	56.6	53.2	56.6	56.6	60.0	60.0	56.6	67.4
roHgla_Leutx	79.9	79.9	84.4	84.4	75.5	94.3	94.3	89.2	94.3	94.3	94.3	94.3	94.3	94.3
roFdam_Leutx	84.4	84.4	89.2	94.3	84.4	99.7	99.7	94.3	99.7	99.7	99.7	99.7	99.7	105.4
roCcan_Leutx	84.4	84.4	89.2	89.2	89.2	75.5	75.5	71.4	75.5	75.5	75.5	75.5	75.5	89.2
roDord_Leutx	75.5	75.5	79.9	84.4	89.2	75.5	75.5	71.4	75.5	75.5	75.5	75.5	75.5	75.5
roClan_LOC102027798	84.4	84.4	89.2	89.2	94.3	89.2	89.2	84.4	89.2	89.2	94.3	94.3	89.2	94.3
roClan_LOC106150198	75.5	75.5	79.9	79.9	84.4	79.9	79.9	75.5	79.9	79.9	84.4	84.4	79.9	84.4
roClan_LOC106147540	94.3	94.3	99.7	99.7	105.4	99.7	99.7	94.3	99.7	99.7	105.4	105.4	99.7	105.4
roClan_LOC102027472	84.4	84.4	89.2	89.2	94.3	89.2	89.2	84.4	89.2	89.2	94.3	94.3	89.2	89.2
roCpor_LOC101788084	89.2	89.2	84.4	99.7	105.4	105.4	105.4	99.7	105.4	105.4	105.4	105.4	105.4	111.5
roCpor_LOC101788138	99.7	99.7	94.3	111.5	118.0	118.0	118.0	111.5	118.0	118.0	118.0	118.0	118.0	125.0
roCpor_LOC101788113	94.3	94.3	89.2	105.4	111.5	111.5	111.5	105.4	111.5	111.5	111.5	111.5	111.5	118.0
roCpor_LOC101788050	105.4	105.4	99.7	111.5	118.0	125.0	125.0	118.0	125.0	125.0	125.0	125.0	125.0	132.6
roCpor_LOC101787990	105.4	105.4	111.5	111.5	125.0	118.0	118.0	111.5	118.0	118.0	118.0	118.0	118.0	125.0
roCpor_LOC101788169	94.3	94.3	89.2	105.4	111.5	111.5	111.5	105.4	111.5	111.5	111.5	111.5	111.5	118.0
roCpor_LOC101788198	94.3	94.3	89.2	105.4	111.5	111.5	111.5	105.4	111.5	111.5	111.5	111.5	111.5	118.0
roMaur_Leutx	105.4	105.4	111.5	111.5	111.5	105.4	105.4	105.4	105.4	105.4	105.4	105.4	105.4	105.4
roCgri_I79_012385	105.4	105.4	111.5	105.4	111.5	105.4	105.4	105.4	105.4	105.4	105.4	105.4	105.4	111.5
roCgri_I79_024957	105.4	105.4	111.5	111.5	111.5	99.7	99.7	99.7	99.7	99.7	99.7	99.7	105.4	118.0
roNlep_A6R68_07039	111.5	111.5	118.0	118.0	118.0	111.5	111.5	111.5	111.5	111.5	111.5	111.5	111.5	111.5
lagOpri_AAYZ01197513	79.9	79.9	84.4	79.9	84.4	89.2	89.2	94.3	89.2	89.2	89.2	89.2	89.2	89.2
lagOcun_LEUTX	94.3	94.3	99.7	94.3	89.2	99.7	99.7	94.3	99.7	99.7	99.7	99.7	99.7	94.3
lagOcun_LOC103348423	99.7	99.7	105.4	99.7	94.3	105.4	105.4	99.7	105.4	105.4	105.4	105.4	105.4	99.7
lagOcun_LOC103348415	99.7	99.7	105.4	94.3	99.7	99.7	99.7	94.3	99.7	99.7	99.7	99.7	99.7	94.3
lagOcun_LOC103348417	99.7	99.7	105.4	99.7	99.7	99.7	99.7	94.3	99.7	99.7	99.7	99.7	99.7	94.3
prHsap_CRX	105.4	105.4	99.7	105.4	105.4	111.5	111.5	111.5	111.5	111.5	111.5	111.5	111.5	111.5
chLcha_CRX	99.7	99.7	94.3	105.4	111.5	105.4	105.4	105.4	105.4	105.4	105.4	105.4	105.4	111.5
chCnil_AAQ08476	99.7	99.7	94.3	105.4	111.5	105.4	105.4	105.4	105.4	105.4	105.4	105.4	105.4	111.5

	prCjac_LEUTX	prSbol_LEUTX	euTchi_LEUTX	euGvar_LEUTX	laOros_LEUTX	laLwed_LEUTX	laClup_AOCS01144808	laFcat_LEUTX	laBtau_LEUTX	laBmut_M91_07100	laBbub_LEUTX	laChir_LEUTX
prHsap_LEUTX	73.3	66.7	60.0	53.3	55.0	51.7	50.0	53.3	50.0	50.0	48.3	50.0
prPpan_LEUTX	73.3	66.7	60.0	53.3	55.0	51.7	50.0	53.3	50.0	50.0	48.3	50.0
prGgor_LEUTX	73.3	66.7	58.3	51.7	55.0	51.7	50.0	53.3	51.7	51.7	50.0	50.0
prPabe_LEUTX	68.3	63.3	56.7	53.3	53.3	50.0	48.3	51.7	50.0	50.0	48.3	50.0
prNleu_LEUTX	66.7	65.0	53.3	50.0	51.7	48.3	46.7	50.0	50.0	50.0	48.3	50.0
prMleu_LEUTX	70.0	63.3	61.7	53.3	53.3	50.0	51.7	55.0	51.7	51.7	50.0	51.7
prCaty_LEUTX	70.0	63.3	61.7	53.3	53.3	50.0	51.7	55.0	51.7	51.7	50.0	51.7
prPanu_LEUTX	71.7	65.0	63.3	55.0	55.0	51.7	53.3	56.7	50.0	50.0	48.3	50.0
prMfas_EGM_09724	70.0	63.3	61.7	53.3	53.3	50.0	51.7	55.0	51.7	51.7	50.0	51.7
prMmul_LOC699783	70.0	63.3	61.7	53.3	53.3	50.0	51.7	55.0	51.7	51.7	50.0	51.7
prCang_LEUTX	70.0	63.3	60.0	53.3	53.3	50.0	51.7	55.0	50.0	50.0	48.3	50.0
prCsab_LEUTX	70.0	63.3	60.0	53.3	53.3	50.0	51.7	55.0	50.0	50.0	48.3	50.0
prRrox_LEUTX	70.0	63.3	61.7	53.3	53.3	50.0	51.7	55.0	51.7	51.7	50.0	51.7
prAnan_LEUTX	86.7	78.3	56.7	53.3	58.3	58.3	56.7	58.3	53.3	53.3	51.7	53.3
prCjac_LEUTX	***	75.0	58.3	51.7	58.3	58.3	56.7	56.7	50.0	50.0	48.3	50.0
prSbol_LEUTX	30.4	***	55.0	51.7	56.7	53.3	51.7	55.0	50.0	50.0	48.3	50.0
euTchi_LEUTX	60.0	67.4	***	60.0	65.0	61.7	60.0	58.3	53.3	53.3	53.3	56.7
euGvar_LEUTX	75.5	75.5	56.6	***	55.0	56.7	53.3	53.3	50.0	50.0	48.3	50.0
laOros_LEUTX	60.0	63.7	46.9	67.4	***	95.0	88.3	83.3	58.3	58.3	58.3	65.0
laLwed_LEUTX	60.0	71.4	53.2	63.7	5.2	***	90.0	86.7	60.0	60.0	60.0	66.7
laClup_AOCS01144808	63.7	75.5	56.6	71.4	12.7	10.8	***	86.7	58.3	58.3	58.3	65.0
laFcat_LEUTX	63.7	67.4	60.0	71.4	18.9	14.7	14.7	***	58.3	58.3	58.3	65.0
laBtau_LEUTX	79.9	79.9	71.4	79.9	60.0	56.6	60.0	60.0	***	100.0	96.7	93.3
laBmut_M91_07100	79.9	79.9	71.4	79.9	60.0	56.6	60.0	60.0	0.0	***	96.7	93.3
laBbub_LEUTX	84.4	84.4	71.4	84.4	60.0	56.6	60.0	60.0	3.4	3.4	***	93.3
laChir_LEUTX	79.9	79.9	63.7	79.9	46.9	43.9	46.9	46.9	7.0	7.0	7.0	***
laPhod_LEUTX	94.3	89.2	75.5	84.4	56.6	53.2	56.6	56.6	16.8	16.8	14.7	8.9
laCfer_LEUTX	75.5	89.2	67.4	75.5	60.0	60.0	67.4	60.0	46.9	46.9	46.9	46.9
laVpac_LEUTX	79.9	94.3	71.4	79.9	60.0	60.0	67.4	60.0	46.9	46.9	46.9	46.9
laSscr_LEUTX	84.4	84.4	67.4	79.9	53.2	53.2	60.0	56.6	43.9	43.9	43.9	41.1
laEcab_AAWR02030248	71.4	67.4	43.9	53.2	33.0	33.0	35.6	30.4	53.2	53.2	53.2	46.9
laEasi_JREZ01000468	71.4	67.4	43.9	56.6	33.0	33.0	35.6	30.4	56.6	56.6	56.6	50.0
laCsim_LOC101387510	63.7	71.4	50.0	53.2	33.0	28.0	33.0	30.4	50.0	50.0	50.0	43.9
laPale_PAL_GLEAN10001506	71.4	75.5	67.4	63.7	50.0	46.9	56.6	53.2	60.0	60.0	60.0	53.2
laMjav_LEUTX	67.4	67.4	53.2	63.7	35.6	33.0	30.4	33.0	63.7	63.7	63.7	50.0
afLafr_LEUTX	89.2	99.7	56.6	84.4	60.0	63.7	63.7	63.7	75.5	75.5	71.4	67.4
afLafr_LEUTX2	89.2	99.7	60.0	89.2	60.0	63.7	63.7	63.7	75.5	75.5	71.4	67.4
xeDnov_LEUTX	71.4	79.9	67.4	75.5	75.5	75.5	71.4	79.9	105.4	105.4	111.5	105.4
roItri_Leutx	60.0	56.6	50.0	71.4	56.6	63.7	67.4	60.0	84.4	84.4	89.2	79.9
roHgla_Leutx	94.3	94.3	67.4	89.2	75.5	75.5	84.4	75.5	84.4	84.4	89.2	84.4
roFdam_Leutx	105.4	99.7	71.4	94.3	79.9	79.9	89.2	79.9	94.3	94.3	99.7	94.3
roCcan_Leutx	89.2	94.3	63.7	63.7	75.5	67.4	75.5	63.7	89.2	89.2	89.2	79.9
roDord_Leutx	71.4	84.4	75.5	79.9	89.2	94.3	99.7	84.4	99.7	99.7	99.7	94.3
roClan_LOC102027798	94.3	94.3	75.5	105.4	99.7	94.3	105.4	99.7	105.4	105.4	111.5	99.7
roClan_LOC106150198	84.4	89.2	67.4	105.4	94.3	89.2	94.3	89.2	99.7	99.7	105.4	94.3
roClan_LOC106147540	105.4	111.5	71.4	111.5	99.7	94.3	105.4	99.7	105.4	105.4	111.5	99.7
roClan_LOC102027472	89.2	84.4	79.9	105.4	94.3	89.2	99.7	94.3	105.4	105.4	111.5	99.7
roCpor_LOC101788084	105.4	118.0	84.4	105.4	99.7	99.7	105.4	105.4	118.0	118.0	125.0	118.0
roCpor_LOC101788138	118.0	118.0	84.4	118.0	94.3	94.3	99.7	99.7	118.0	118.0	118.0	118.0
roCpor_LOC101788113	111.5	111.5	84.4	111.5	94.3	94.3	99.7	99.7	111.5	111.5	118.0	111.5
roCpor_LOC101788050	125.0	125.0	89.2	125.0	99.7	99.7	105.4	105.4	111.5	111.5	118.0	111.5
roCpor_LOC101787990	125.0	132.6	84.4	99.7	105.4	105.4	111.5	111.5	132.6	132.6	132.6	132.6
roCpor_LOC101788169	111.5	118.0	75.5	111.5	94.3	94.3	99.7	99.7	118.0	118.0	125.0	118.0
roCpor_LOC101788198	111.5	118.0	75.5	111.5	94.3	94.3	99.7	99.7	118.0	118.0	125.0	118.0
roMaur_Leutx	118.0	125.0	94.3	94.3	118.0	125.0	132.6	118.0	140.9	140.9	140.9	140.9
roCgri_I79_012385	118.0	125.0	89.2	94.3	118.0	125.0	132.6	118.0	140.9	140.9	140.9	140.9
roCgri_I79_024957	125.0	125.0	89.2	94.3	118.0	125.0	132.6	118.0	140.9	140.9	140.9	140.9
roNlep_A6R68_07039	118.0	125.0	94.3	89.2	118.0	125.0	125.0	118.0	132.6	132.6	132.6	125.0
lagOpri_AAYZ01197513	94.3	84.4	79.9	89.2	75.5	84.4	84.4	89.2	99.7	99.7	105.4	94.3
lagOcun_LEUTX	99.7	99.7	71.4	75.5	67.4	63.7	67.4	63.7	79.9	79.9	79.9	67.4
lagOcun_LOC103348423	105.4	105.4	75.5	79.9	71.4	67.4	71.4	67.4	84.4	84.4	84.4	71.4
lagOcun_LOC103348415	99.7	105.4	79.9	94.3	71.4	67.4	71.4	75.5	79.9	79.9	79.9	79.9
lagOcun_LOC103348417	99.7	105.4	84.4	84.4	84.4	79.9	84.4	84.4	84.4	84.4	84.4	79.9
prHsap_CRX	111.5	99.7	89.2	132.6	99.7	111.5	118.0	118.0	118.0	118.0	125.0	118.0
chLcha_CRX	111.5	94.3	84.4	125.0	94.3	105.4	111.5	111.5	105.4	105.4	111.5	105.4
chCnil_AAQ08476	111.5	94.3	84.4	125.0	94.3	105.4	111.5	111.5	105.4	105.4	111.5	105.4

	laPhod_LEUTX	laCfer_LEUTX	laVpac_LEUTX	laSscr_LEUTX	laEcab_AAWR02030248	laEasi_JREZ01000468	laCsim_LOC101387510	laPale_PAL_GLEAN10001506	laMjav_LEUTX	afLafr_LEUTX	afLafr_LEUTX2	xeDnov_LEUTX	roItri_Leutx	roHgla_Leutx	roFdam_Leutx	roCcan_Leutx
prHsap_LEUTX	45.0	46.7	45.0	43.3	51.7	51.7	50.0	50.0	53.3	46.7	46.7	50.0	56.7	50.0	48.3	48.3
prPpan_LEUTX	45.0	46.7	45.0	43.3	51.7	51.7	50.0	50.0	53.3	46.7	46.7	50.0	56.7	50.0	48.3	48.3
prGgor_LEUTX	45.0	48.3	46.7	45.0	53.3	53.3	51.7	51.7	53.3	45.0	45.0	48.3	55.0	48.3	46.7	46.7
prPabe_LEUTX	45.0	50.0	48.3	43.3	51.7	51.7	50.0	50.0	51.7	45.0	45.0	48.3	55.0	48.3	45.0	46.7
prNleu_LEUTX	45.0	46.7	45.0	43.3	48.3	48.3	48.3	48.3	51.7	41.7	41.7	46.7	55.0	51.7	48.3	46.7
prMleu_LEUTX	46.7	45.0	43.3	41.7	51.7	51.7	48.3	48.3	51.7	50.0	50.0	50.0	60.0	45.0	43.3	51.7
prCaty_LEUTX	46.7	45.0	43.3	41.7	51.7	51.7	48.3	48.3	51.7	50.0	50.0	50.0	60.0	45.0	43.3	51.7
prPanu_LEUTX	45.0	46.7	45.0	43.3	53.3	53.3	50.0	50.0	53.3	51.7	51.7	51.7	61.7	46.7	45.0	53.3
prMfas_EGM_09724	46.7	45.0	43.3	41.7	51.7	51.7	48.3	48.3	51.7	50.0	50.0	50.0	60.0	45.0	43.3	51.7
prMmul_LOC699783	46.7	45.0	43.3	41.7	51.7	51.7	48.3	48.3	51.7	50.0	50.0	50.0	60.0	45.0	43.3	51.7
prCang_LEUTX	45.0	45.0	43.3	41.7	51.7	51.7	48.3	48.3	51.7	48.3	48.3	51.7	58.3	45.0	43.3	51.7
prCsab_LEUTX	45.0	45.0	43.3	41.7	51.7	51.7	50.0	48.3	51.7	48.3	48.3	50.0	58.3	45.0	43.3	51.7
prRrox_LEUTX	46.7	45.0	43.3	41.7	51.7	51.7	48.3	48.3	51.7	50.0	50.0	50.0	60.0	45.0	43.3	51.7
prAnan_LEUTX	48.3	51.7	50.0	50.0	53.3	53.3	56.7	51.7	55.0	51.7	51.7	58.3	55.0	45.0	41.7	46.7
prCjac_LEUTX	45.0	51.7	50.0	48.3	53.3	53.3	56.7	53.3	55.0	46.7	46.7	53.3	58.3	45.0	41.7	46.7
prSbol_LEUTX	46.7	46.7	45.0	48.3	55.0	55.0	53.3	51.7	55.0	43.3	43.3	50.0	60.0	45.0	43.3	45.0
euTchi_LEUTX	51.7	55.0	53.3	55.0	66.7	66.7	63.3	55.0	61.7	60.0	58.3	55.0	63.3	55.0	53.3	56.7
euGvar_LEUTX	48.3	51.7	50.0	50.0	61.7	60.0	61.7	56.7	56.7	48.3	46.7	51.7	53.3	46.7	45.0	56.7
laOros_LEUTX	60.0	58.3	58.3	61.7	73.3	73.3	73.3	63.3	71.7	58.3	58.3	51.7	60.0	51.7	50.0	51.7
laLwed_LEUTX	61.7	58.3	58.3	61.7	73.3	73.3	76.7	65.0	73.3	56.7	56.7	51.7	56.7	51.7	50.0	55.0
laClup_AOCS01144808	60.0	55.0	55.0	58.3	71.7	71.7	73.3	60.0	75.0	56.7	56.7	53.3	55.0	48.3	46.7	51.7
laFcat_LEUTX	60.0	58.3	58.3	60.0	75.0	75.0	75.0	61.7	73.3	56.7	56.7	50.0	58.3	51.7	50.0	56.7
laBtau_LEUTX	85.0	65.0	65.0	66.7	61.7	60.0	63.3	58.3	56.7	51.7	51.7	41.7	48.3	48.3	45.0	46.7
laBmut_M91_07100	85.0	65.0	65.0	66.7	61.7	60.0	63.3	58.3	56.7	51.7	51.7	41.7	48.3	48.3	45.0	46.7
laBbub_LEUTX	86.7	65.0	65.0	66.7	61.7	60.0	63.3	58.3	56.7	53.3	53.3	40.0	46.7	46.7	43.3	46.7
laChir_LEUTX	91.7	65.0	65.0	68.3	65.0	63.3	66.7	61.7	63.3	55.0	55.0	41.7	50.0	48.3	45.0	50.0
laPhod_LEUTX	***	58.3	58.3	61.7	60.0	58.3	63.3	56.7	56.7	51.7	51.7	40.0	46.7	43.3	43.3	45.0
laCfer_LEUTX	60.0	***	98.3	83.3	63.3	65.0	61.7	66.7	61.7	46.7	46.7	45.0	51.7	53.3	50.0	55.0
laVpac_LEUTX	60.0	1.7	***	83.3	61.7	63.3	60.0	65.0	60.0	46.7	46.7	43.3	50.0	53.3	50.0	53.3
laSscr_LEUTX	53.2	18.9	18.9	***	65.0	66.7	65.0	68.3	66.7	48.3	48.3	43.3	51.7	53.3	50.0	50.0
laEcab_AAWR02030248	56.6	50.0	53.2	46.9	***	98.3	88.3	73.3	75.0	60.0	60.0	48.3	61.7	55.0	56.7	55.0
laEasi_JREZ01000468	60.0	46.9	50.0	43.9	1.7	***	86.7	71.7	75.0	58.3	58.3	48.3	63.3	53.3	55.0	56.7
laCsim_LOC101387510	50.0	53.2	56.6	46.9	12.7	14.7	***	71.7	75.0	61.7	61.7	50.0	56.7	50.0	51.7	55.0
laPale_PAL_GLEAN10001506	63.7	43.9	46.9	41.1	33.0	35.6	35.6	***	66.7	50.0	50.0	45.0	53.3	50.0	46.7	50.0
laMjav_LEUTX	63.7	53.2	56.6	43.9	30.4	30.4	30.4	43.9	***	55.0	55.0	50.0	58.3	48.3	46.7	50.0
afLafr_LEUTX	75.5	89.2	89.2	84.4	56.6	60.0	53.2	79.9	67.4	***	95.0	48.3	50.0	43.3	41.7	45.0
afLafr_LEUTX2	75.5	89.2	89.2	84.4	56.6	60.0	53.2	79.9	67.4	5.2	***	48.3	50.0	43.3	41.7	45.0
xeDnov_LEUTX	111.5	94.3	99.7	99.7	84.4	84.4	79.9	94.3	79.9	84.4	84.4	***	55.0	43.3	40.0	50.0
roItri_Leutx	89.2	75.5	79.9	75.5	53.2	50.0	63.7	71.4	60.0	79.9	79.9	67.4	***	51.7	50.0	55.0
roHgla_Leutx	99.7	71.4	71.4	71.4	67.4	71.4	79.9	79.9	84.4	99.7	99.7	99.7	75.5	***	90.0	48.3
roFdam_Leutx	99.7	79.9	79.9	79.9	63.7	67.4	75.5	89.2	89.2	105.4	105.4	111.5	79.9	10.8	***	46.7
roCcan_Leutx	94.3	67.4	71.4	79.9	67.4	63.7	67.4	79.9	79.9	94.3	94.3	79.9	67.4	84.4	89.2	***
roDord_Leutx	105.4	99.7	105.4	105.4	84.4	89.2	89.2	84.4	94.3	94.3	94.3	79.9	71.4	94.3	105.4	71.4
roClan_LOC102027798	105.4	99.7	99.7	94.3	84.4	79.9	84.4	111.5	94.3	118.0	118.0	105.4	63.7	67.4	71.4	89.2
roClan_LOC106150198	111.5	99.7	99.7	94.3	89.2	89.2	89.2	105.4	84.4	111.5	111.5	94.3	63.7	56.6	67.4	89.2
roClan_LOC106147540	118.0	94.3	94.3	89.2	94.3	89.2	94.3	111.5	99.7	118.0	118.0	111.5	67.4	63.7	75.5	89.2
roClan_LOC102027472	111.5	105.4	105.4	99.7	79.9	75.5	79.9	105.4	89.2	111.5	111.5	105.4	63.7	63.7	67.4	99.7
roCpor_LOC101788084	132.6	99.7	99.7	99.7	89.2	89.2	99.7	89.2	99.7	105.4	111.5	118.0	84.4	67.4	71.4	111.5
roCpor_LOC101788138	118.0	111.5	111.5	99.7	84.4	84.4	94.3	89.2	94.3	105.4	111.5	125.0	89.2	67.4	71.4	125.0
roCpor_LOC101788113	125.0	105.4	105.4	94.3	84.4	84.4	94.3	84.4	94.3	111.5	118.0	125.0	89.2	67.4	71.4	118.0
roCpor_LOC101788050	125.0	105.4	105.4	94.3	89.2	89.2	99.7	94.3	99.7	132.6	140.9	132.6	94.3	71.4	75.5	125.0
roCpor_LOC101787990	125.0	111.5	111.5	118.0	94.3	94.3	105.4	111.5	105.4	105.4	111.5	111.5	89.2	67.4	71.4	105.4
roCpor_LOC101788169	132.6	105.4	105.4	99.7	84.4	84.4	94.3	89.2	94.3	111.5	118.0	118.0	84.4	60.0	63.7	118.0
roCpor_LOC101788198	132.6	105.4	105.4	99.7	84.4	84.4	94.3	89.2	94.3	111.5	118.0	118.0	84.4	60.0	63.7	118.0
roMaur_Leutx	132.6	149.9	159.9	140.9	118.0	125.0	118.0	125.0	111.5	118.0	118.0	125.0	94.3	105.4	118.0	118.0
roCgri_I79_012385	140.9	149.9	159.9	140.9	118.0	118.0	118.0	125.0	118.0	125.0	125.0	125.0	99.7	118.0	125.0	118.0
roCgri_I79_024957	140.9	149.9	159.9	140.9	118.0	118.0	118.0	125.0	118.0	125.0	125.0	125.0	99.7	118.0	125.0	118.0
roNlep_A6R68_07039	132.6	140.9	149.9	140.9	94.3	99.7	94.3	125.0	99.7	94.3	99.7	118.0	99.7	111.5	118.0	118.0
lagOpri_AAYZ01197513	89.2	111.5	111.5	105.4	75.5	75.5	79.9	89.2	94.3	94.3	94.3	99.7	84.4	89.2	84.4	89.2
lagOcun_LEUTX	71.4	89.2	89.2	79.9	56.6	60.0	50.0	63.7	63.7	79.9	79.9	89.2	63.7	71.4	79.9	71.4
lagOcun_LOC103348423	75.5	94.3	94.3	84.4	60.0	63.7	53.2	67.4	67.4	84.4	84.4	94.3	67.4	75.5	84.4	75.5
lagOcun_LOC103348415	84.4	79.9	79.9	71.4	71.4	71.4	60.0	79.9	75.5	89.2	89.2	84.4	79.9	89.2	94.3	105.4
lagOcun_LOC103348417	84.4	89.2	89.2	75.5	79.9	75.5	67.4	84.4	84.4	99.7	99.7	84.4	79.9	89.2	99.7	79.9
prHsap_CRX	118.0	99.7	105.4	105.4	94.3	94.3	94.3	99.7	105.4	105.4	105.4	89.2	84.4	99.7	105.4	149.9
chLcha_CRX	111.5	89.2	94.3	94.3	84.4	84.4	89.2	84.4	99.7	99.7	99.7	94.3	89.2	111.5	118.0	140.9
chCnil_AAQ08476	111.5	89.2	94.3	94.3	84.4	84.4	89.2	84.4	99.7	99.7	99.7	94.3	89.2	111.5	118.0	140.9

	roDord_Leutx	roClan_LOC102027798	roClan_LOC106150198	roClan_LOC106147540	roClan_LOC102027472	roCpor_LOC101788084	roCpor_LOC101788138	roCpor_LOC101788113	roCpor_LOC101788050	roCpor_LOC101787990	roCpor_LOC101788169	roCpor_LOC101788198	roMaur_Leutx
prHsap_LEUTX	51.7	48.3	51.7	45.0	48.3	46.7	43.3	45.0	41.7	41.7	45.0	45.0	41.7
prPpan_LEUTX	51.7	48.3	51.7	45.0	48.3	46.7	43.3	45.0	41.7	41.7	45.0	45.0	41.7
prGgor_LEUTX	50.0	46.7	50.0	43.3	46.7	48.3	45.0	46.7	43.3	40.0	46.7	46.7	40.0
prPabe_LEUTX	48.3	46.7	50.0	43.3	46.7	43.3	40.0	41.7	40.0	40.0	41.7	41.7	40.0
prNleu_LEUTX	46.7	45.0	48.3	41.7	45.0	41.7	38.3	40.0	38.3	36.7	40.0	40.0	40.0
prMleu_LEUTX	51.7	46.7	50.0	43.3	46.7	41.7	38.3	40.0	36.7	38.3	40.0	40.0	41.7
prCaty_LEUTX	51.7	46.7	50.0	43.3	46.7	41.7	38.3	40.0	36.7	38.3	40.0	40.0	41.7
prPanu_LEUTX	53.3	48.3	51.7	45.0	48.3	43.3	40.0	41.7	38.3	40.0	41.7	41.7	41.7
prMfas_EGM_09724	51.7	46.7	50.0	43.3	46.7	41.7	38.3	40.0	36.7	38.3	40.0	40.0	41.7
prMmul_LOC699783	51.7	46.7	50.0	43.3	46.7	41.7	38.3	40.0	36.7	38.3	40.0	40.0	41.7
prCang_LEUTX	51.7	45.0	48.3	41.7	45.0	41.7	38.3	40.0	36.7	38.3	40.0	40.0	41.7
prCsab_LEUTX	51.7	45.0	48.3	41.7	45.0	41.7	38.3	40.0	36.7	38.3	40.0	40.0	41.7
prRrox_LEUTX	51.7	46.7	50.0	43.3	46.7	41.7	38.3	40.0	36.7	38.3	40.0	40.0	41.7
prAnan_LEUTX	51.7	45.0	48.3	41.7	46.7	40.0	36.7	38.3	35.0	36.7	38.3	38.3	41.7
prCjac_LEUTX	53.3	45.0	48.3	41.7	46.7	41.7	38.3	40.0	36.7	36.7	40.0	40.0	38.3
prSbol_LEUTX	48.3	45.0	46.7	40.0	48.3	38.3	38.3	40.0	36.7	35.0	38.3	38.3	36.7
euTchi_LEUTX	51.7	51.7	55.0	53.3	50.0	48.3	48.3	48.3	46.7	48.3	51.7	51.7	45.0
euGvar_LEUTX	50.0	41.7	41.7	40.0	41.7	41.7	38.3	40.0	36.7	43.3	40.0	40.0	45.0
laOros_LEUTX	46.7	43.3	45.0	43.3	45.0	43.3	45.0	45.0	43.3	41.7	45.0	45.0	38.3
laLwed_LEUTX	45.0	45.0	46.7	45.0	46.7	43.3	45.0	45.0	43.3	41.7	45.0	45.0	36.7
laClup_AOCS01144808	43.3	41.7	45.0	41.7	43.3	41.7	43.3	43.3	41.7	40.0	43.3	43.3	35.0
laFcat_LEUTX	48.3	43.3	46.7	43.3	45.0	41.7	43.3	43.3	41.7	40.0	43.3	43.3	38.3
laBtau_LEUTX	43.3	41.7	43.3	41.7	41.7	38.3	38.3	40.0	40.0	35.0	38.3	38.3	33.3
laBmut_M91_07100	43.3	41.7	43.3	41.7	41.7	38.3	38.3	40.0	40.0	35.0	38.3	38.3	33.3
laBbub_LEUTX	43.3	40.0	41.7	40.0	40.0	36.7	38.3	38.3	38.3	35.0	36.7	36.7	33.3
laChir_LEUTX	45.0	43.3	45.0	43.3	43.3	38.3	38.3	40.0	40.0	35.0	38.3	38.3	33.3
laPhod_LEUTX	41.7	41.7	40.0	38.3	40.0	35.0	38.3	36.7	36.7	36.7	35.0	35.0	35.0
laCfer_LEUTX	43.3	43.3	43.3	45.0	41.7	43.3	40.0	41.7	41.7	40.0	41.7	41.7	31.7
laVpac_LEUTX	41.7	43.3	43.3	45.0	41.7	43.3	40.0	41.7	41.7	40.0	41.7	41.7	30.0
laSscr_LEUTX	41.7	45.0	45.0	46.7	43.3	43.3	43.3	45.0	45.0	38.3	43.3	43.3	33.3
laEcab_AAWR02030248	48.3	48.3	46.7	45.0	50.0	46.7	48.3	48.3	46.7	45.0	48.3	48.3	38.3
laEasi_JREZ01000468	46.7	50.0	46.7	46.7	51.7	46.7	48.3	48.3	46.7	45.0	48.3	48.3	36.7
laCsim_LOC101387510	46.7	48.3	46.7	45.0	50.0	43.3	45.0	45.0	43.3	41.7	45.0	45.0	38.3
laPale_PAL_GLEAN10001506	48.3	40.0	41.7	40.0	41.7	46.7	46.7	48.3	45.0	40.0	46.7	46.7	36.7
laMjav_LEUTX	45.0	45.0	48.3	43.3	46.7	43.3	45.0	45.0	43.3	41.7	45.0	45.0	40.0
afLafr_LEUTX	45.0	38.3	40.0	38.3	40.0	41.7	41.7	40.0	35.0	41.7	40.0	40.0	38.3
afLafr_LEUTX2	45.0	38.3	40.0	38.3	40.0	40.0	40.0	38.3	33.3	40.0	38.3	38.3	38.3
xeDnov_LEUTX	50.0	41.7	45.0	40.0	41.7	38.3	36.7	36.7	35.0	40.0	38.3	38.3	36.7
roItri_Leutx	53.3	56.7	56.7	55.0	56.7	48.3	46.7	46.7	45.0	46.7	48.3	48.3	45.0
roHgla_Leutx	45.0	55.0	60.0	56.7	56.7	55.0	55.0	55.0	53.3	55.0	58.3	58.3	41.7
roFdam_Leutx	41.7	53.3	55.0	51.7	55.0	53.3	53.3	53.3	51.7	53.3	56.7	56.7	38.3
roCcan_Leutx	53.3	46.7	46.7	46.7	43.3	40.0	36.7	38.3	36.7	41.7	38.3	38.3	38.3
roDord_Leutx	***	43.3	45.0	43.3	43.3	43.3	41.7	43.3	41.7	38.3	43.3	43.3	41.7
roClan_LOC102027798	99.7	***	91.7	90.0	93.3	55.0	55.0	55.0	53.3	51.7	56.7	56.7	41.7
roClan_LOC106150198	94.3	8.9	***	91.7	91.7	56.7	56.7	56.7	55.0	51.7	60.0	60.0	41.7
roClan_LOC106147540	99.7	10.8	8.9	***	86.7	55.0	55.0	55.0	55.0	50.0	58.3	58.3	38.3
roClan_LOC102027472	99.7	7.0	8.9	14.7	***	53.3	53.3	53.3	51.7	50.0	56.7	56.7	40.0
roCpor_LOC101788084	99.7	67.4	63.7	67.4	71.4	***	95.0	98.3	90.0	71.7	96.7	96.7	33.3
roCpor_LOC101788138	105.4	67.4	63.7	67.4	71.4	5.2	***	96.7	91.7	73.3	96.7	96.7	36.7
roCpor_LOC101788113	99.7	67.4	63.7	67.4	71.4	1.7	3.4	***	91.7	70.0	96.7	96.7	33.3
roCpor_LOC101788050	105.4	71.4	67.4	67.4	75.5	10.8	8.9	8.9	***	65.0	91.7	91.7	31.7
roCpor_LOC101787990	118.0	75.5	75.5	79.9	79.9	35.6	33.0	38.3	46.9	***	71.7	71.7	36.7
roCpor_LOC101788169	99.7	63.7	56.6	60.0	63.7	3.4	3.4	3.4	8.9	35.6	***	100.0	35.0
roCpor_LOC101788198	99.7	63.7	56.6	60.0	63.7	3.4	3.4	3.4	8.9	35.6	0.0	***	35.0
roMaur_Leutx	105.4	105.4	105.4	118.0	111.5	140.9	125.0	140.9	149.9	125.0	132.6	132.6	***
roCgri_I79_012385	118.0	118.0	118.0	132.6	125.0	125.0	118.0	125.0	132.6	125.0	118.0	118.0	23.3
roCgri_I79_024957	118.0	118.0	118.0	132.6	125.0	125.0	118.0	125.0	132.6	125.0	118.0	118.0	25.6
roNlep_A6R68_07039	99.7	111.5	111.5	118.0	105.4	132.6	118.0	132.6	140.9	111.5	125.0	125.0	33.0
lagOpri_AAYZ01197513	105.4	99.7	105.4	118.0	105.4	99.7	105.4	99.7	118.0	99.7	99.7	99.7	125.0
lagOcun_LEUTX	79.9	84.4	75.5	79.9	79.9	94.3	94.3	94.3	99.7	89.2	84.4	84.4	118.0
lagOcun_LOC103348423	84.4	89.2	79.9	84.4	84.4	99.7	99.7	99.7	105.4	94.3	89.2	89.2	125.0
lagOcun_LOC103348415	111.5	99.7	89.2	94.3	94.3	118.0	118.0	118.0	125.0	125.0	105.4	105.4	149.9
lagOcun_LOC103348417	99.7	94.3	89.2	89.2	89.2	111.5	111.5	111.5	118.0	111.5	99.7	99.7	132.6
prHsap_CRX	105.4	111.5	111.5	118.0	105.4	125.0	125.0	125.0	132.6	132.6	118.0	118.0	183.8
chLcha_CRX	111.5	111.5	111.5	118.0	105.4	125.0	125.0	125.0	140.9	132.6	118.0	118.0	171.1
chCnil_AAQ08476	111.5	111.5	111.5	118.0	105.4	125.0	125.0	125.0	140.9	132.6	118.0	118.0	171.1

	roCgri_I79_012385	roCgri_I79_024957	roNlep_A6R68_07039	lagOpri_AAYZ01197513	lagOcun_LEUTX	lagOcun_LOC103348423	lagOcun_LOC103348415	lagOcun_LOC103348417	prHsap_CRX	chLcha_CRX	chCnil_AAQ08476
prHsap_LEUTX	41.7	41.7	40.0	50.0	45.0	43.3	43.3	43.3	41.7	43.3	43.3
prPpan_LEUTX	41.7	41.7	40.0	50.0	45.0	43.3	43.3	43.3	41.7	43.3	43.3
prGgor_LEUTX	40.0	40.0	38.3	48.3	43.3	41.7	41.7	41.7	43.3	45.0	45.0
prPabe_LEUTX	41.7	40.0	38.3	50.0	45.0	43.3	45.0	43.3	41.7	41.7	41.7
prNleu_LEUTX	40.0	40.0	38.3	48.3	46.7	45.0	43.3	43.3	41.7	40.0	40.0
prMleu_LEUTX	41.7	43.3	40.0	46.7	43.3	41.7	43.3	43.3	40.0	41.7	41.7
prCaty_LEUTX	41.7	43.3	40.0	46.7	43.3	41.7	43.3	43.3	40.0	41.7	41.7
prPanu_LEUTX	41.7	43.3	40.0	45.0	45.0	43.3	45.0	45.0	40.0	41.7	41.7
prMfas_EGM_09724	41.7	43.3	40.0	46.7	43.3	41.7	43.3	43.3	40.0	41.7	41.7
prMmul_LOC699783	41.7	43.3	40.0	46.7	43.3	41.7	43.3	43.3	40.0	41.7	41.7
prCang_LEUTX	41.7	43.3	40.0	46.7	43.3	41.7	43.3	43.3	40.0	41.7	41.7
prCsab_LEUTX	41.7	43.3	40.0	46.7	43.3	41.7	43.3	43.3	40.0	41.7	41.7
prRrox_LEUTX	41.7	41.7	40.0	46.7	43.3	41.7	43.3	43.3	40.0	41.7	41.7
prAnan_LEUTX	40.0	38.3	40.0	46.7	45.0	43.3	45.0	45.0	40.0	40.0	40.0
prCjac_LEUTX	38.3	36.7	38.3	45.0	43.3	41.7	43.3	43.3	40.0	40.0	40.0
prSbol_LEUTX	36.7	36.7	36.7	48.3	43.3	41.7	41.7	41.7	43.3	45.0	45.0
euTchi_LEUTX	46.7	46.7	45.0	50.0	53.3	51.7	50.0	48.3	46.7	48.3	48.3
euGvar_LEUTX	45.0	45.0	46.7	46.7	51.7	50.0	45.0	48.3	35.0	36.7	36.7
laOros_LEUTX	38.3	38.3	38.3	51.7	55.0	53.3	53.3	48.3	43.3	45.0	45.0
laLwed_LEUTX	36.7	36.7	36.7	48.3	56.7	55.0	55.0	50.0	40.0	41.7	41.7
laClup_AOCS01144808	35.0	35.0	36.7	48.3	55.0	53.3	53.3	48.3	38.3	40.0	40.0
laFcat_LEUTX	38.3	38.3	38.3	46.7	56.7	55.0	51.7	48.3	38.3	40.0	40.0
laBtau_LEUTX	33.3	33.3	35.0	43.3	50.0	48.3	50.0	48.3	38.3	41.7	41.7
laBmut_M91_07100	33.3	33.3	35.0	43.3	50.0	48.3	50.0	48.3	38.3	41.7	41.7
laBbub_LEUTX	33.3	33.3	35.0	41.7	50.0	48.3	50.0	48.3	36.7	40.0	40.0
laChir_LEUTX	33.3	33.3	36.7	45.0	55.0	53.3	50.0	50.0	38.3	41.7	41.7
laPhod_LEUTX	33.3	33.3	35.0	46.7	53.3	51.7	48.3	48.3	38.3	40.0	40.0
laCfer_LEUTX	31.7	31.7	33.3	40.0	46.7	45.0	50.0	46.7	43.3	46.7	46.7
laVpac_LEUTX	30.0	30.0	31.7	40.0	46.7	45.0	50.0	46.7	41.7	45.0	45.0
laSscr_LEUTX	33.3	33.3	33.3	41.7	50.0	48.3	53.3	51.7	41.7	45.0	45.0
laEcab_AAWR02030248	38.3	38.3	45.0	51.7	60.0	58.3	53.3	50.0	45.0	48.3	48.3
laEasi_JREZ01000468	38.3	38.3	43.3	51.7	58.3	56.7	53.3	51.7	45.0	48.3	48.3
laCsim_LOC101387510	38.3	38.3	45.0	50.0	63.3	61.7	58.3	55.0	45.0	46.7	46.7
laPale_PAL_GLEAN10001506	36.7	36.7	36.7	46.7	56.7	55.0	50.0	48.3	43.3	48.3	48.3
laMjav_LEUTX	38.3	38.3	43.3	45.0	56.7	55.0	51.7	48.3	41.7	43.3	43.3
afLafr_LEUTX	36.7	36.7	45.0	45.0	50.0	48.3	46.7	43.3	41.7	43.3	43.3
afLafr_LEUTX2	36.7	36.7	43.3	45.0	50.0	48.3	46.7	43.3	41.7	43.3	43.3
xeDnov_LEUTX	36.7	36.7	38.3	43.3	46.7	45.0	48.3	48.3	46.7	45.0	45.0
roItri_Leutx	43.3	43.3	43.3	48.3	56.7	55.0	50.0	50.0	48.3	46.7	46.7
roHgla_Leutx	38.3	38.3	40.0	46.7	53.3	51.7	46.7	46.7	43.3	40.0	40.0
roFdam_Leutx	36.7	36.7	38.3	48.3	50.0	48.3	45.0	43.3	41.7	38.3	38.3
roCcan_Leutx	38.3	38.3	38.3	46.7	53.3	51.7	41.7	50.0	31.7	33.3	33.3
roDord_Leutx	38.3	38.3	43.3	41.7	50.0	48.3	40.0	43.3	41.7	40.0	40.0
roClan_LOC102027798	38.3	38.3	40.0	43.3	48.3	46.7	43.3	45.0	40.0	40.0	40.0
roClan_LOC106150198	38.3	38.3	40.0	41.7	51.7	50.0	46.7	46.7	40.0	40.0	40.0
roClan_LOC106147540	35.0	35.0	38.3	38.3	50.0	48.3	45.0	46.7	38.3	38.3	38.3
roClan_LOC102027472	36.7	36.7	41.7	41.7	50.0	48.3	45.0	46.7	41.7	41.7	41.7
roCpor_LOC101788084	36.7	36.7	35.0	43.3	45.0	43.3	38.3	40.0	36.7	36.7	36.7
roCpor_LOC101788138	38.3	38.3	38.3	41.7	45.0	43.3	38.3	40.0	36.7	36.7	36.7
roCpor_LOC101788113	36.7	36.7	35.0	43.3	45.0	43.3	38.3	40.0	36.7	36.7	36.7
roCpor_LOC101788050	35.0	35.0	33.3	38.3	43.3	41.7	36.7	38.3	35.0	33.3	33.3
roCpor_LOC101787990	36.7	36.7	40.0	43.3	46.7	45.0	36.7	40.0	35.0	35.0	35.0
roCpor_LOC101788169	38.3	38.3	36.7	43.3	48.3	46.7	41.7	43.3	38.3	38.3	38.3
roCpor_LOC101788198	38.3	38.3	36.7	43.3	48.3	46.7	41.7	43.3	38.3	38.3	38.3
roMaur_Leutx	80.0	78.3	73.3	36.7	38.3	36.7	31.7	35.0	26.7	28.3	28.3
roCgri_I79_012385	***	98.3	71.7	38.3	36.7	35.0	33.3	36.7	26.7	28.3	28.3
roCgri_I79_024957	1.7	***	70.0	38.3	36.7	35.0	33.3	36.7	26.7	28.3	28.3
roNlep_A6R68_07039	35.6	38.3	***	36.7	43.3	41.7	33.3	38.3	30.0	31.7	31.7
lagOpri_AAYZ01197513	118.0	118.0	125.0	***	55.0	53.3	48.3	48.3	41.7	43.3	43.3
lagOcun_LEUTX	125.0	125.0	99.7	67.4	***	98.3	73.3	78.3	43.3	45.0	45.0
lagOcun_LOC103348423	132.6	132.6	105.4	71.4	1.7	***	71.7	76.7	41.7	43.3	43.3
lagOcun_LOC103348415	140.9	140.9	140.9	84.4	33.0	35.6	***	85.0	38.3	41.7	41.7
lagOcun_LOC103348417	125.0	125.0	118.0	84.4	25.6	28.0	16.8	***	36.7	40.0	40.0
prHsap_CRX	183.8	183.8	159.9	105.4	99.7	105.4	118.0	125.0	***	86.7	86.7
chLcha_CRX	171.1	171.1	149.9	99.7	94.3	99.7	105.4	111.5	14.7	***	100.0
chCnil_AAQ08476	171.1	171.1	149.9	99.7	94.3	99.7	105.4	111.5	14.7	0.0	***

Table S3. Leutx homeodomain distance matrix. Sequence Distances were calculated (PAM250) with MegAlign. Percent identity in upper triangle; percent Divergence in lower triangle.

Chromosome (GRCh38/hg38)	Chromosomal position	Exon	Intron	cDNA position	CDS position	Protein position	Homeodo main position	Reference	Call	rsIDs	Codons	Amino acids	Maximum consequence	HGVSc	HGVSp	Max allele frequency	Gnomad: number of homozygotes	deCode: call quality	deCode: number of genotyped	deCode: total depth	deCode: allele frequency
chr19	39784519		1					C	A				splice_region_variant,intron_variant	c.8-8C>A		6.6E-06	0	NA	NA	NA	NA
chr19	39784527	2		46	8	3		A	G		gAa/gGa	E/G	missense_variant,splice_region_variant	c.8A>G	p.Glu3Gly	0.0000131	0	NA	NA	NA	NA
chr19	39784536	2		55	17	6		G	A	rs550435255	aGg/aAg	R/K	missense_variant	c.17G>A	p.Arg6Lys	0.003497	0	NA	NA	NA	NA
chr19	39784538	2		57	19	7		C	T	rs761890692	Cgt/Tgt	R/C	missense_variant	c.19C>T	p.Arg7Cys	0.000137	0	NA	NA	NA	NA
chr19	39784539	2		58	20	7		G	A	rs765232053	cGt/cAt	R/H	missense_variant	c.20G>A	p.Arg7His	0.000135	0	NA	NA	NA	NA
chr19	39784545	2		64	26	9	2	G	A	rs17709621	cGt/cAt	R/H	missense_variant	c.26G>A	p.Arg9His	0.287234	814	0	28074	934499	0.104554
chr19	39784548	2		67	29	10	3	G	A	rs758599556	cGg/cAg	R/Q	missense_variant	c.29G>A	p.Arg10Gln	0.000127	0	NA	NA	NA	NA
chr19	39784554	2		73	35	12	5	G	A	rs117937250	cGc/cAc	R/H	missense_variant	c.35G>A	p.Arg12His	0.001	0	NA	NA	NA	NA
chr19	39784574	2		93	55	19	12	C	A	rs547099889	Caa/Aaa	Q/K	missense_variant	c.55C>A	p.Gln19Lys	0.0008	0	NA	NA	NA	NA
chr19	39784577	2		96	58	20	13	C	T	rs755346374	Ctc/Ttc	L/F	missense_variant	c.58C>T	p.Leu20Phe	0.00165	0	NA	NA	NA	NA
chr19	39784578	2		97	59	20	13	T	C	rs781608781	cTc/cCc	L/P	missense_variant	c.59T>C	p.Leu20Pro	0.000254	0	NA	NA	NA	NA
chr19	39784597	2		116	78	26	19	G	T		ttG/ttT	L/F	missense_variant	c.78G>T	p.Leu26Phe	0	0	NA	NA	NA	NA
chr19	39784601	2		120	82	28	21	G	A		Gaa/Aaa	E/K	missense_variant	c.82G>A	p.Glu28Lys	0.003333	0	NA	NA	NA	NA
chr19	39784612	2		131	93	31	24	G	A		atG/atA	M/I	missense_variant	c.93G>A	p.Met31Ile	0.00000639	0	NA	NA	NA	NA
chr19	39784614	2		133	95	32	25	A	G	rs544666908	cAc/cGc	H/R	missense_variant	c.95A>G	p.His32Arg	0.004167	0	0	28074	942973	1.78129E-05
chr19	39784616	2		135	97	33	26	C	T	rs745474654	Cca/Tca	P/S	missense_variant	c.97C>T	p.Pro33Ser	0.000128	0	NA	NA	NA	NA
chr19	39784617	2		136	98	33	26	C	G	rs745425368	cCa/cGa	P/R	missense_variant	c.98C>G	p.Pro33Arg	0.000115	0	NA	NA	NA	NA
chr19	39784620	2		139	101	34	27	G	A		aGt/aAt	S/N	missense_variant	c.101G>A	p.Ser34Asn	0.00000639	0	NA	NA	NA	NA
chr19	39784623	2		142	104	35	28	T	C		tTg/tCg	L/S	missense_variant	c.104T>C	p.Leu35Ser	3.56214E-05	0	0	28074	957937	3.56214E-05
chr19	39784634	2		153	115	39	32	G	A	rs771812228	Ggg/Agg	G/R	missense_variant	c.115G>A	p.Gly39Arg	0.000126	0	NA	NA	NA	NA
chr19	39784644	2		163	125	42	35	C	A		gCt/gAt	A/D	missense_variant	c.125C>A	p.Ala42Asp	0.0000661	0	NA	NA	NA	NA
chr19	39784670	2		189	151	51	44	G	A	rs117228099	Gta/Ata	V/I	missense_variant	c.151G>A	p.Val51Ile	0.002439	0	NA	NA	NA	NA
chr19	39784670	2		189	151	51	44	G	T	rs117228099	Gta/Tta	V/L	missense_variant	c.151G>T	p.Val51Leu	0.000115	0	NA	NA	NA	NA
chr19	39784678	2		197	159	53	46	G	A	rs866067011	aaG/aaA	K	splice_region_variant,synonymous_variant	c.159G>A	c.159G>A(p.%3D)	0.0000661	0	NA	NA	NA	NA
chr19	39784679		2					G	A				splice_donor_variant	c.159+1G>A		0.0000064	0	NA	NA	NA	NA
chr19	39784679		2					G	T				splice_donor_variant	c.159+1G>T		0.0000064	0	NA	NA	NA	NA
chr19	39784685		2					T	A				splice_region_variant,intron_variant	c.159+7T>A		0.0000064	0	NA	NA	NA	NA
chr19	39784686		2					C	G				splice_region_variant,intron_variant	c.159+8C>G		0.00000641	0	NA	NA	NA	NA
chr19	39785690		2					C	T				splice_region_variant,intron_variant	c.160-8C>T		0.00000643	0	NA	NA	NA	NA
chr19	39785692		2					C	T				splice_region_variant,intron_variant	c.160-6C>T		0.00000637	0	NA	NA	NA	NA
chr19	39785694		2					T	A				splice_region_variant,intron_variant	c.160-4T>A		0.00000637	0	NA	NA	NA	NA
chr19	39785700	3		200	162	54	47	C	T		atC/atT	I	splice_region_variant,synonymous_variant	c.162C>T	c.162C>T(p.%3D)	0.00000637	0	NA	NA	NA	NA
chr19	39785716	3		216	178	60	53	C	A	rs577142991	Cgt/Agt	R/S	missense_variant	c.178C>A	p.Arg60Ser	0.001	0	NA	NA	NA	NA
chr19	39785716	3		216	178	60	53	C	T		Cgt/Tgt	R/C	missense_variant	c.178C>T	p.Arg60Cys	0.0000191	0	NA	NA	NA	NA
chr19	39785717	3		217	179	60	53	G	A		cGt/cAt	R/H	missense_variant	c.179G>A	p.Arg60His	0.00000636	0	NA	NA	NA	NA
chr19	39785720	3		220	182	61	54	C	T	rs541307025	gCc/gTc	A/V	missense_variant	c.182C>T	p.Ala61Val	0.001	0	NA	NA	NA	NA
chr19	39785725	3		225	187	63	56	T	A	rs745314487	Tgg/Agg	W/R	missense_variant	c.187T>A	p.Trp63Arg	0.000383	0	NA	NA	NA	NA
chr19	39785728	3		228	190	64	57	A	G		Aag/Gag	K/E	missense_variant	c.190A>G	p.Lys64Glu	0.00000636	0	NA	NA	NA	NA
chr19	39785740	3		240	202	68		C	T	rs376692805	Cgg/Tgg	R/W	missense_variant	c.202C>T	p.Arg68Trp	0.000127	0	NA	NA	NA	NA
chr19	39785741	3		241	203	68		G	A		cGg/cAg	R/Q	missense_variant	c.203G>A	p.Arg68Gln	0.0000127	0	NA	NA	NA	NA
chr19	39785741	3		241	203	68		G	T		cGg/cTg	R/L	missense_variant	c.203G>T	p.Arg68Leu	0.00000637	0	NA	NA	NA	NA
chr19	39785754	3		254	216	72		G	C		caG/caC	Q/H	missense_variant	c.216G>C	p.Gln72His	0.0000331	0	NA	NA	NA	NA
chr19	39785758	3		258	220	74		C	T	rs558962748	Cgg/Tgg	R/W	missense_variant	c.220C>T	p.Arg74Trp	0.003333	0	NA	NA	NA	NA
chr19	39785759	3		259	221	74		G	A		cGg/cAg	R/Q	missense_variant	c.221G>A	p.Arg74Gln	0.0000128	0	NA	NA	NA	NA
chr19	39785764	3		264	226	76		T	C		Tca/Cca	S/P	missense_variant	c.226T>C	p.Ser76Pro	0.00000638	0	NA	NA	NA	NA
chr19	39785767	3		267	229	77		C	A		Cta/Ata	L/I	missense_variant	c.229C>A	p.Leu77Ile	0.00000638	0	NA	NA	NA	NA
chr19	39785783	3		283	245	82		A	C	rs369685173	cAg/cCg	Q/P	missense_variant	c.245A>C	p.Gln82Pro	0.001391	0	NA	NA	NA	NA
chr19	39785786	3		287-289	249-251	83-84		CAAC	C	rs759722545	acAACt/act	TT/T	inframe_deletion	c.249_251delAAC	p.Thr84del	0.000113	0	NA	NA	NA	NA
chr19	39785789	3		289	251	84		C	T		aCt/aTt	T/I	missense_variant	c.251C>T	p.Thr84Ile	0.000033	0	NA	NA	NA	NA
chr19	39785792	3		292	254	85		C	T	rs768484764	tCa/tTa	S/L	missense_variant	c.254C>T	p.Ser85Leu	0.000126	0	NA	NA	NA	NA
chr19	39785801	3		301	263	88		A	G		aAg/aGg	K/R	missense_variant	c.263A>G	p.Lys88Arg	0.0000128	0	NA	NA	NA	NA
chr19	39785804	3		304	266	89		A	C	rs776270599	gAg/gCg	E/A	missense_variant	c.266A>C	p.Glu89Ala	0.000114	0	NA	NA	NA	NA
chr19	39785812	3		312	274	92		C	A	rs529686425	Ccc/Acc	P/T	missense_variant	c.274C>A	p.Pro92Thr	0.001608	0	NA	NA	NA	NA
chr19	39785813	3		313	275	92		C	T	rs773212414	cCc/cTc	P/L	missense_variant	c.275C>T	p.Pro92Leu	0.000466	0	NA	NA	NA	NA
chr19	39785815	3		315	277	93		T	C	rs766439043	Tca/Cca	S/P	missense_variant	c.277T>C	p.Ser93Pro	0.0129	0	0	28075	912145	5.61584E-05
chr19	39785821	3		321	283	95		A	G	rs774471747	Ata/Gta	I/V	missense_variant	c.283A>G	p.Ile95Val	0.000126	0	NA	NA	NA	NA
chr19	39785821	3		321	283	95		A	T		Ata/Tta	I/L	missense_variant	c.283A>T	p.Ile95Leu	0.0000161	0	NA	NA	NA	NA
chr19	39785827	3		327	289	97		A	G		Act/Gct	T/A	missense_variant	c.289A>G	p.Thr97Ala	0.00000639	0	NA	NA	NA	NA
chr19	39785839	3		339	301	101		C	G	rs547985951	Cgt/Ggt	R/G	missense_variant	c.301C>G	p.Arg101Gly	0.002451	0	NA	NA	NA	NA
chr19	39785839	3		339	301	101		C	T		Cgt/Tgt	R/C	missense_variant	c.301C>T	p.Arg101Cys	0.0000321	0	NA	NA	NA	NA
chr19	39785840	3		340	302	101		G	A	rs373629971	cGt/cAt	R/H	missense_variant	c.302G>A	p.Arg101His	0.0014	0	NA	NA	NA	NA
chr19	39785845	3		345	307	103		G	A	rs752965786	Gta/Ata	V/I	missense_variant	c.307G>A	p.Val103Ile	0.000114	0	NA	NA	NA	NA
chr19	39785865	3		365	327	109		T	G		gaT/gaG	D/E	missense_variant	c.327T>G	p.Asp109Glu	0.00000639	0	NA	NA	NA	NA
chr19	39785867	3		367	329	110		C	T		gCa/gTa	A/V	missense_variant	c.329C>T	p.Ala110Val	0.002688	0	NA	NA	NA	NA
chr19	39785870	3		370	332	111		A	T		aAt/aTt	N/I	missense_variant	c.332A>T	p.Asn111Ile	0.00000639	0	NA	NA	NA	NA
chr19	39785874	3		374	336	112		C	A		gaC/gaA	D/E	missense_variant	c.336C>A	p.Asp112Glu	0.000033	0	NA	NA	NA	NA
chr19	39785879	3		379	341	114		A	G	rs577998458	gAt/gGt	D/G	missense_variant	c.341A>G	p.Asp114Gly	0.001	0	NA	NA	NA	NA
chr19	39785884	3		384	346	116		C	T		Cgt/Tgt	R/C	missense_variant	c.346C>T	p.Arg116Cys	0.0000192	0	NA	NA	NA	NA

chr19	39785885	3	385	347	116	G	A		cGt/cAt	R/H	missense_variant	c.347G>A	p.Arg116His	0.5	0	NA	NA	NA	NA
chr19	39785902	3	402	364	122	A	G	rs141754227	Aag/Gag	K/E	missense_variant	c.364A>G	p.Lys122Glu	0.001	0	NA	NA	NA	NA
chr19	39785904	3	404	366	122	G	T	rs552374784	aaG/aaT	K/N	missense_variant	c.366G>T	p.Lys122Asn	0.0061	0	NA	NA	NA	NA
chr19	39785911	3	411	373	125	G	A		Gga/Aga	G/R	missense_variant	c.373G>A	p.Gly125Arg	0.0000107	0	NA	NA	NA	NA
chr19	39785918	3	418	380	127	C	T	rs757909677	gCc/gTc	A/V	missense_variant	c.380C>T	p.Ala127Val	0.000114	0	NA	NA	NA	NA
chr19	39785918	3	418	380	127	C	G		gCc/gGc	A/G	missense_variant	c.380C>G	p.Ala127Gly	0.0000331	0	NA	NA	NA	NA
chr19	39785922	3	422	384	128	C	G		agC/agG	S/R	missense_variant	c.384C>G	p.Ser128Arg	0.00000639	0	NA	NA	NA	NA
chr19	39785923	3	423	385	129	G	T	rs751166546	Gcc/Tcc	A/S	missense_variant	c.385G>T	p.Ala129Ser	0.000196348	0	0	28075	966396	0.000196348
chr19	39785923	3	423	385	129	G	A	rs751166546	Gcc/Acc	A/T	missense_variant	c.385G>A	p.Ala129Thr	0.000126	0	NA	NA	NA	NA
chr19	39785927	3	427	389	130	C	T		tCt/tTt	S/F	missense_variant	c.389C>T	p.Ser130Phe	0.0000128	0	NA	NA	NA	NA
chr19	39785930	3	430	392	131	C	T	rs754558994	gCg/gTg	A/V	missense_variant	c.392C>T	p.Ala131Val	0.000379	0	NA	NA	NA	NA
chr19	39785934	3	434	396	132	G	C	rs534950736	agG/agC	R/S	missense_variant	c.396G>C	p.Arg132Ser	0.007902	0	NA	NA	NA	NA
chr19	39785945	3	445	407	136	G	C		tGg/tCg	W/S	missense_variant	c.407G>C	p.Trp136Ser	0.000033	0	NA	NA	NA	NA
chr19	39785960	3	460	422	141	A	G		tAt/tGt	Y/C	missense_variant	c.422A>G	p.Tyr141Cys	0.00000639	0	NA	NA	NA	NA
chr19	39785962	3	462	424	142	G	A		Gac/Aac	D/N	missense_variant	c.424G>A	p.Asp142Asn	0.0000128	0	NA	NA	NA	NA
chr19	39785965	3	465	427	143	A	G		Att/Gtt	I/V	missense_variant	c.427A>G	p.Ile143Val	0.004878	0	NA	NA	NA	NA
chr19	39785976	3	476-477	438-439	146-147	A	AA				frameshift_variant,feature_elongation	c.438dupA	p.Cys147MetfsTer15	0.002331	0	NA	NA	NA	NA
chr19	39785981	3	481	443	148	T	A		cTg/cAg	L/Q	missense_variant	c.443T>A	p.Leu148Gln	0.0000192	0	NA	NA	NA	NA
chr19	39785987	3	488-490	450-452	150-151	CTTC	C	rs532813481	gcTTCa/gca	AS/A	inframe_deletion	c.450_452delTTC	p.Ser151del	0.001002	0	NA	NA	NA	NA
chr19	39785994	3	494	456	152	T	A		aaT/aaA	N/K	missense_variant	c.456T>A	p.Asn152Lys	0.000124678	0	0	28075	946849	0.000124678
chr19	39785995	3	495	457	153	C	A		Cct/Act	P/T	missense_variant	c.457C>A	p.Pro153Thr	0.000033	0	NA	NA	NA	NA
chr19	39786001	3	501	463	155	T	C		Tgg/Cgg	W/R	missense_variant	c.463T>C	p.Trp155Arg	0.00000639	0	NA	NA	NA	NA
chr19	39786002	3	502	464	155	G	A	rs780917699	tGg/tAg	W/*	stop_gained	c.464G>A	p.Trp155Ter	0.000115	0	NA	NA	NA	NA
chr19	39786011	3	511	473	158	C	A		aCt/aAt	T/N	missense_variant	c.473C>A	p.Thr158Asn	0.0000064	0	NA	NA	NA	NA
chr19	39786013	3	513	475	159	C	G		Ctc/Gtc	L/V	missense_variant	c.475C>G	p.Leu159Val	0.0000064	0	NA	NA	NA	NA
chr19	39786016	3	516	478	160	T	C		Ttt/Ctt	F/L	missense_variant	c.478T>C	p.Phe160Leu	0.0000128	0	NA	NA	NA	NA
chr19	39786023	3	523	485	162	T	G	rs750419185	aTa/aGa	I/R	missense_variant	c.485T>G	p.Ile162Arg	0.000929	0	NA	NA	NA	NA
chr19	39786023	3	523	485	162	T	C		aTa/aCa	I/T	missense_variant	c.485T>C	p.Ile162Thr	0	0	NA	NA	NA	NA
chr19	39786025	3	525	487	163	G	C		Gat/Cat	D/H	missense_variant	c.487G>C	p.Asp163His	0.0000331	0	NA	NA	NA	NA
chr19	39786028	3	528	490	164	G	A		Gaa/Aaa	E/K	missense_variant	c.490G>A	p.Glu164Lys	0.0000268	0	NA	NA	NA	NA
chr19	39786030	3	530	492	164	A	C		gaA/gaC	E/D	missense_variant	c.492A>C	p.Glu164Asp	0.00000641	0	NA	NA	NA	NA
chr19	39786050	3	550	512	171	T	A		tTg/tAg	L/*	stop_gained	c.512T>A	p.Leu171Ter	0.0000331	0	NA	NA	NA	NA
chr19	39786051	3	551	513	171	G	T		ttG/ttT	L/F	missense_variant	c.513G>T	p.Leu171Phe	0.0000129	0	NA	NA	NA	NA
chr19	39786056	3	556	518	173	G	A	rs777493010	gGg/gAg	G/E	missense_variant	c.518G>A	p.Gly173Glu	0.000119	0	0	28075	927262	3.56209E-05
chr19	39786064	3	564	526	176	G	A		Gac/Aac	D/N	missense_variant	c.526G>A	p.Asp176Asn	0.00000649	0	NA	NA	NA	NA
chr19	39786067	3	567	529	177	A	C	rs61995735	Acc/Ccc	T/P	missense_variant	c.529A>C	p.Thr177Pro	0.0325	8	NA	NA	NA	NA
chr19	39786077	3	578-579	540-541	180-181	TAA	T				frameshift_variant,feature_truncation	c.540_541delAA	p.Asn181SerfsTer9	0.0000331	0	NA	NA	NA	NA
chr19	39786080	3	580	542	181	A	G		aAt/aGt	N/S	missense_variant	c.542A>G	p.Asn181Ser	0.0000331	0	NA	NA	NA	NA
chr19	39786082	3	582	544	182	C	T		Caa/Taa	Q/*	stop_gained	c.544C>T	p.Gln182Ter	0.0000331	0	NA	NA	NA	NA
chr19	39786086	3	586	548	183	A	G	rs770903563	tAt/tGt	Y/C	missense_variant	c.548A>G	p.Tyr183Cys	0.002551	0	NA	NA	NA	NA
chr19	39786101	3	601	563	188	G	A	rs147692654	tGc/tAc	C/Y	missense_variant	c.563G>A	p.Cys188Tyr	0.003378	0	NA	NA	NA	NA
chr19	39786110	3	610	572	191	A	G	rs772138251	tAt/tGt	Y/C	missense_variant	c.572A>G	p.Tyr191Cys	0.000143	0	NA	NA	NA	NA
chr19	39786114	3	614	576	192	C	A	rs571718226	gaC/gaA	D/E	missense_variant	c.576C>A	p.Asp192Glu	0.000329	0	NA	NA	NA	NA
chr19	39786117	3	617	579	193	G	T		caG/caT	Q/H	missense_variant	c.579G>T	p.Gln193His	0.0000331	0	NA	NA	NA	NA
chr19	39786117	3	617	579	193	G	C		caG/caC	Q/H	missense_variant	c.579G>C	p.Gln193His	0.00000756	0	NA	NA	NA	NA
chr19	39786124	3	624	586	196	T	C		Tct/Cct	S/P	missense_variant	c.586T>C	p.Ser196Pro	0.00000792	0	NA	NA	NA	NA
chr19	39786131	3	631	593	198	T	C		gTg/gCg	V/A	missense_variant	c.593T>C	p.Val198Ala	0.00000831	0	NA	NA	NA	NA
chr19	39786131	3	631	593	198	T	G		gTg/gGg	V/G	missense_variant	c.593T>G	p.Val198Gly	0.00000831	0	NA	NA	NA	NA

Table S4. 116 known variants of human *Leutx*. Blue highlighted are four common amino-acid changing variants (max. allele frequency ≥1%). Yellow highlighted variants are potentially deleterious when homozygous, affecting conserved residues in known/presumed functional regions, or truncating the protein.

Gene name	NCBI accession number
ARGFX	LN901395, LN901396
CPHX1	LN651082, LN651083
CPHX2	LN651086, LN651087
DPRX	LN651088
LEUTX	LN651090
TPRX1	LN901411, LN901412
TPRX2	LN901405, LN901406
OTX2	NM_172337.2

Table S5. Accession numbers for the sequences of the PRD-LIKE TFs.

Name	Sequence
11_bp_Fw	ATATGGTCTCCGCTAGCTGTAATCCCAGGCTAGCGATATCTGTAATCCCAGGATATCCTAGCTTAGCTAGGCCTCGGCGGCC
11_bp_Rv	ATATGGTCTCCTAGCGAGCTCCTGGGATTACAGAGCTCGGTACCCTGGGATTACAAGGTACCGGCCAGTTAGGCCAGAGAAA
pGL4_Fw	GCTTTACCAACAGTACCGGAT
pGL4_Rv	TAGCAAAATAGGCTGTCCCC
LEUTX_mut1_F	TATCCGTAGTAAAGACCTGGTTCAAGAACCA
LEUTX_mut1_R	TGGTTCTTGAACCAGGTCTTTACTACGGATA
LEUTX_mut3_F	TCAAGAACCAGCGTGTCAAATGGAAGAGGCA
LEUTX_mut3_R	TGCCTCTTCCATTTGACACGCTGGTTCTTGA

Table S6. Primer sequences used.
